# Synthesis and Selective Anticancer Activity Evaluation of 2-phenylacrylonitrile Derivatives as Tubulin Inhibitors

**DOI:** 10.2174/0109298673263854231009063053

**Published:** 2024-02-26

**Authors:** Ye-Zhi Jin, Ya-Bing Xin, Yuan Li, Xin-Yuan Chen, De-Ao Man, Yu-Shun Tian

**Affiliations:** 1 Key Laboratory of Natural Medicines of the Changbai Mountain, Department of Medicinal Chemistry, Ministry of Education, College of Pharmacy, Yanbian University, Yanji, 133002, Jilin Province, P.R. China

**Keywords:** 2-Phenylacrylonitrile, selective toxic effect, cell cycle arrest, apoptosis, xenograft model, tubulin inhibitor

## Abstract

**Objective:**

This study aimed at synthesizing 13 series of novel derivatives with 2-phenylacrylonitrile, evaluating antitumor activity both *in vivo* and *in vitro*, and obtaining novel tubulin inhibitors.

**Methods:**

The 13 series of 2-phenylacrylonitrile derivatives were synthesized by Knoevenagel condensation and the anti-proliferative activities were determined by MTT assay. The cell cycle and apoptosis were analyzed by flow cytometer. Quantitative cell migration was performed using 24-well Boyden chambers. The proteins were detected by Western blotting. *In vitro* kinetics of microtubule assembly was measured using ELISA kit for Human β-tubulin (TUBB). Molecular docking was done by Discovery Studio (DS) 2017 Client online tool.

**Results:**

Among the derivatives, compound **1g2a** possessed strong inhibitory activity against HCT116 (IC_50_ = 5.9 nM) and BEL-7402 (IC_50_ = 7.8 nM) cells. Compound **1g2a** exhibited better selective antiproliferative activities and specificities than all the positive control drugs, including taxol. Compound **1g2a** inhibited proliferation of HCT116 and BEL-7402 cells by arresting them in the G2/M phase of the cell cycle, inhibited the migration of HCT116 and BEL-7402 cells and the formation of cell colonies. Compound **1g2a** showed excellent tubulin polymerization inhibitory activity on HCT116 and BEL-7402 cells. The results of molecular docking analyses showed that **1g2a** may inhibit tubulin to exert anticancer effects.

**Conclusion:**

Compound **1g2a** shows outstanding antitumor activity both *in vivo* and *in vitro* and has the potential to be further developed into a highly effective antitumor agent with little toxicity to normal tissues.

## 1 INTRODUCTION

In 2019, an estimated 1,335,100 new cancer cases and 3,97,583 cancer-related deaths occurred among adolescents and young adults worldwide [1]. Many chemotherapies have been developed, but there is no ideal anticancer drug that can kill cancer cells without threatening normal human tissues. It has become a huge challenge for modern science to find targeted, low-toxicity, high-efficiency antitumor drugs [2]. Natural products play a leading role in drug discovery and many of the new drugs approved for marketing were directly or indirectly derived from natural products [3]. Thus, the structural discovery of new drugs is crucial.

**Fig. (1):**
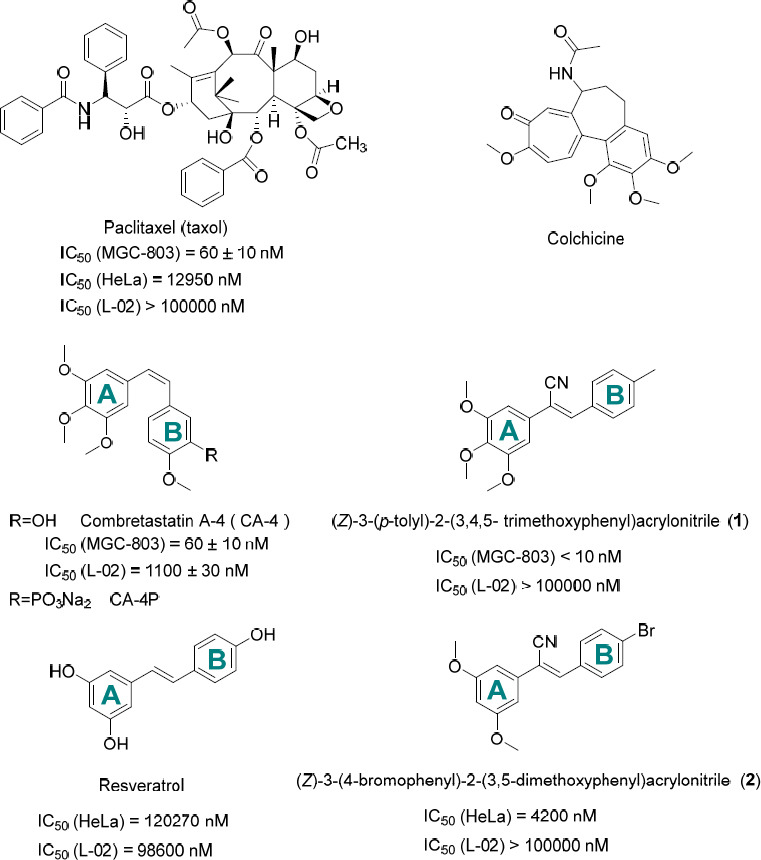
Structures of taxol, colchicine, CA-4, CA-4P, (Z)-3-(*p*-tolyl)-2-(3,4,5-trimethoxyphenyl)acrylonitrile (**1**), resveratrol and (*Z*)-3-(4- bromophenyl)-2-(3,5-dimethoxyphenyl)acrylonitrile (**2**).

**Fig. (2):**
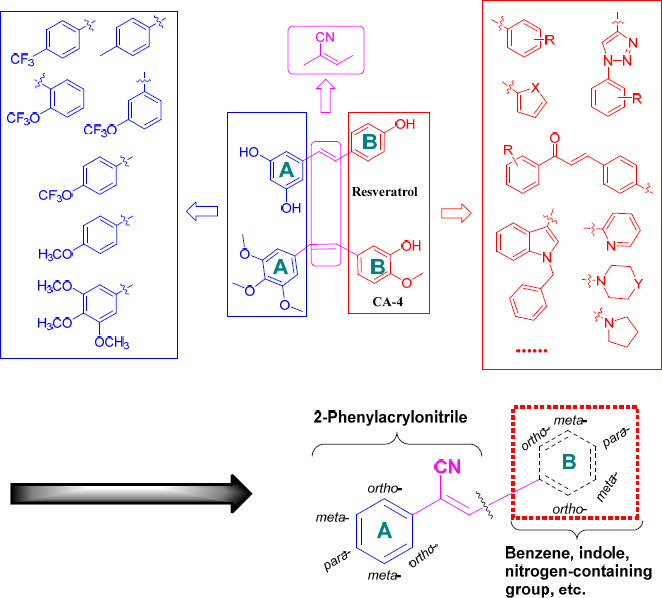
Design derivatives with 2-phenylacrylonitrile to achieve potential anti-proliferative activity.

Derivatives of natural products, such as combretastatin A-4 (CA-4) (Fig. **1**), resveratrol (Fig. **1**), and pterostilbene, are widely used as anticancer candidates [4]. These molecules all have a stilbene [also known as 1,2-diphenylethene] scaffold, which is a component of a number of biologically active natural and synthetic compounds [4, 5]. For example, CA-4 was proven to possess a wide variety of pharmacological activities, including anticancer effects [6, 7]. Recent studies have found that the 3,4,5-trimethoxyphenyl fragment of CA-4 derivatives has a prevention of tubulin polymerization effect by binding to the colchicine binding site [8]. Microtubules are a major component of the cytoskeleton consisting of α- and β-tubulin heterodimers, which play crucial roles in several cellular processes [9, 10]. It is one of the best targets for developing anti-cancer drugs. Various naturally occurring molecules are well known for their anti-tubulin effect, such as vinca (vinca binding site), paclitaxel (taxol binding site), combretastatin (colchicine binding site), colchicine (colchicine binding site) *etc.* Destroying the microtubule skeleton is effective in cancer chemotherapy [9, 11]. Unfortunately, *in vivo*, CA-4 showed a sharp decline in its anti-tubulin and cytotoxic activity due to the spontaneous isomerization of the ethylene bond from the *cis*-isomer to the more stable *trans*-isomer [12], limiting its application in clinical practice. To restrict the *cis*-orientation of the ethylene bond, some researchers chose to substitute the ethylene bond with rigid heterocyclic rings, such as pyridine, pyrrole, pyrazole, triazoles, imidazole, isoxazole, and azetidine [11, 13-18]. Through these attempts, compounds with good anticancer effects have been obtained, but they have not been developed into new drugs. Some studies have found that when a cyano group is introduced into the ethylene structure of stilbenes, the configuration can be fixed to *cis*- and some other compounds, which have a 3,4,5-trimethoxyphenyl fragment and show strong antiproliferative activity against cancer cells without toxicity to normal cells [19, 20]. The inhibitory activity of the representative compound **1** toward MGC-803 cancer cells was stronger than that of CA-4 and taxol (Fig. **1**). Compounds with a 3,5-dimethoxyphenyl fragment instead of a 3,4,5-trimethoxyphenyl fragment show comparable effects against cancer cells [2]. The selectivity index (SI) of compound **2** containing a 3,5-dimethoxyphenyl fragment for HeLa cancer cells and L-02 normal cells was higher than that of taxol and resveratrol (Fig. **1**). It can be inferred that a stilbene with a cyano group on the ethylene bond is likely to be a candidate for the basis of anticancer drugs with low toxicity and high efficiency. If the substituents on the A-benzene ring and B-benzene ring of the stilbene are changed, or the structure of the B-benzene ring is changed, the activity may be affected (Fig. **2**).

More than 3800 naturally occurring halogenated compounds have been found, and many of these have good biological activity [21]. Therefore, halogen-containing groups, including trifluoromethyl or trifluoromethoxy groups, are often introduced into compounds to obtain new drugs with enhanced anticancer activity [22-24]. 1,2,3-Triazole has a high dipole moment and can form a hydrogen bond with drug targets [16]. It is an important heterocyclic structural unit distributed in a large number of biologically active molecules [25]. In recent years, natural active products and the 1,2,3-triazole molecular fragment were combined to improve antitumor activity and drug-forming properties of natural products [26]. Furthermore, the anticancer activity of target compounds can be affected by addition of a pentabasic cyclic ring, hexatomic ring, or fused ring containing nitrogen, sulfur, or no heteroatom [27-32].

In this study, we selected CA-4 as a lead compound and introduced a cyano group into the ethylene bond to fix the stilbene configuration. The A benzene ring was substituted with -OCF_3_, -OCH_3_, -CF_3_, or -CH_3_, and the B benzene ring was substituted (*e.g.*, with amino, halogen, or alkoxy groups) or replaced (*e.g.*, with a triazole, chalcone, or piperidine fragment). Hence, we designed and synthesized multiple series of derivatives with 2-phenylacrylonitrile (a type of stilbene with a cyano group into the ethylene bond and lack of B benzene ring) (Fig. **2**). Subsequently, all 83 new compounds (Fig. **S1**) were screened against 11 different types of cancer cell lines. The toxicity of the compounds was also tested in normal human liver L-02 and breast MCF-10A cells. Additionally, the *in vivo* antitumor efficacy and mechanism of action of the most promising compound, **1g2a**, was investigated.

## 2 MATERIALS AND METHODS

All specific materials and pharmacological experimental methods can be found in the supplementary material.

## 3 RESULTS AND DISCUSSION

### 3.1 Chemistry

**Fig. (3):**
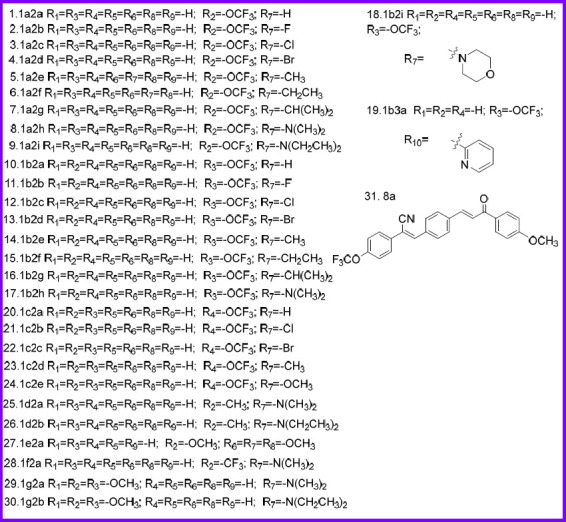
The compounds and their substituents were synthesized according to Scheme **1** and Scheme **2**.

**Scheme (1):**
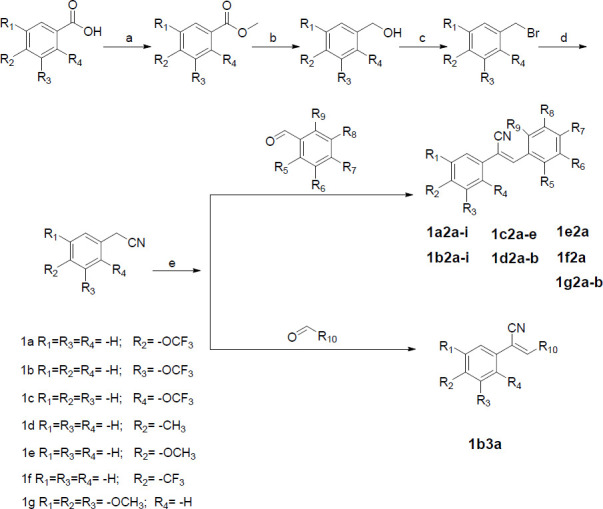
Reagents and conditions: (**a**) H_2_SO_4_, CH_3_OH, reflux, 4 h; (**b**) LiAlH_4_, THF, 0°C-rt, 4-6 h; (**c**) CH_2_Cl_2_, PBr_3_, 0°C-rt, 3-6 h; (**d**) CH_3_CN, TMSCN, TBAF, reflux, 4-6 h; (**e**) CH_3_OH, CH_3_ONa, aromatic aldehydes, 4-6 h.

**Scheme (2):**
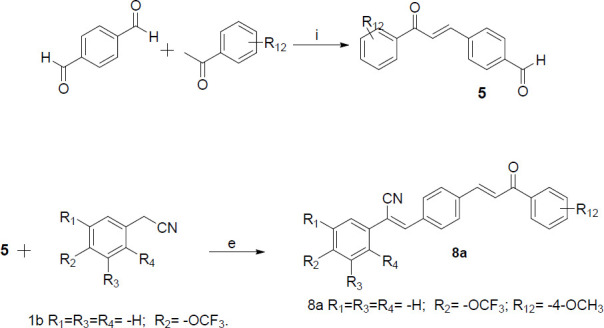
Reagents and conditions: (**e**) CH_3_OH, CH_3_ONa, aromatic aldehydes, 4-6 h; (**i**) KOH, dry ethanol, stirred at rt, 12 h.

According to Scheme **S1** and Scheme **S2** obtained 13 series of derivatives. The substituents of the target compounds synthesized by Scheme **1** and Scheme **2** are shown in Fig. (**3**). Before biological evaluation, the compounds (Fig. **3**) were confirmed by ^1^H NMR and ^13^C NMR spectrometry as well as high-resolution mass spectrometry (supplementary material), and the *Z* isomer was confirmed by NOE (nuclear overhauser effect) (Fig. **S2**).

### 3.2 Biological Evaluation

#### 3.2.1 *In Vitro* Anti-proliferative Activity and SAR Study

The anti-proliferation activities of the synthesized analogs were assessed by an *in vitro* MTT assay conducted on thirteen human cell lines. Their cytotoxic activities were evaluated by measuring the inhibition of net cell growth. The IC_50_ values are listed in Table **1** and Table **S1**. CA-4, CA-4P, taxol, colchicine, and resveratrol are used as positive controls.

Introduction of the -OCF_3_ group at the *p*-position of benzene ring A yielded **1a2a**-**1a2u**. When the *p*-position of the B-ring was substituted by -Cl (**1a2c**) or -Br (**1a2d**), the compounds showed antiproliferation activity against 10 tumor cell lines. Moreover, this series of compounds showed very weak inhibitory activities against both L-02 and MCF-10A normal cells. When the B-ring was substituted with an alkyl group, compound **1a2g** showed the strongest antiproliferation activity against AGS cells (IC_50_ = 8.7 µmol/L). The antiproliferative activity against AGS cells was related to the length of the alkyl substituent chain at the *p*-position on the B-ring. When the B-ring was replaced by an alkoxy group, only **1a2j** (*p*-OCH_3_) and **1a2m** (*p*-OCH_2_CH_2_CH_3_) showed weak inhibition of proliferation of a few tumor cell lines (Table **S1**). However, there was some enhancement in antiproliferative activity when the B-ring was substituted with a nitrogen-containing group. For instance, compound **1a2h** [*p*-N(CH_3_)_2_] showed strong antiproliferation activity against HCT116 cells (IC_50_ = 8.7 µmol/L). When the B-ring was substituted with a nitrogen-containing group, the antiproliferative activity decreased with the increase of carbon chain length connected to the nitrogen atom (**1a2h**-**i** and **1a2s**-**u**). When the B-ring was changed to a furan (**1a3a**), thiophene (**1a3b**), or naphthalene (**1a3c**) ring, only compound **1a3b** showed weak antiproliferative activity against a few kinds of cancer cells. However, when the B-ring was replaced with a chalcone fragment obtaining compounds **8a** and **8c**, they showed good antiproliferation activities against Raji and SU-DHL-10 cells, with IC_50_ values ~1 µmol/L. The antiproliferation effect of **8d** against these two types of cells was also quite obvious. However, there was almost no antiproliferation activity when the B-ring was replaced with triazole (**7a**-**7c**) or indole (**9**). In summary, we found that when the B-ring was replaced by a larger ring fragment or possessed a larger substituent (other than chalcone), the synthesized compounds showed decreased or no antiproliferative activity. On the basis of the above experimental results, we made further modifications.

Next, we introduced an -OCF_3_ group at the ortho (*o*)- or meta (*m*)- position of the A benzene ring. The compounds in which the *m*-position of the A-ring was substituted with -OCF_3_ and a halogen was introduced into the B-ring (**1b2b**-**1b2d**) generally showed good antiproliferative activity. Among them, the introduction of -F resulted in compound **1b2b**, which had potent activity against HCT116, MGC-803, BEL-7402, SGC-7901, and AGS cells with IC_50_ values of 1.1, 1.2, 3.0, 5.4, and 8.2 µmol/L, respectively. Introduction of a methyl group at the *p*-position of the B-ring yielded compound **1b2e**, which had good inhibitory activity of proliferation of AGS (IC_50_ = 4.5 µmol/L) and SGC-7901 (IC_50_ = 8.1 µmol/L) cells. The antiproliferative activities of compounds **1b2e**-**1b2g** decreased with the extension of the carbon chain when an alkyl group was introduced at the *p*-position of the B-ring. However, when introducing an alkoxy group at the *p*-position of the B-ring, the synthesized compounds (**1b2j**-**1b2k**) had little antiproliferative activity. When introducing a nitrogen-containing group at the *p*-position of the B-ring or replacing the B-ring with a heterocycle, the compounds (**1b2h**, **1b2i**, and **1b3a**) showed strong inhibitory effects on the proliferation of a variety of cancer cells. Among these compounds, **1b2h** [*p*-N(CH_3_)_2_] exerted potent antiproliferative activity against BEL-7402 (IC_50_ = 1.0 µmol/L), HCT116 (IC_50_ = 0.3 µmol/L), MGC-803 (IC_50_ = 3.7 µmol/L), and SGC-7901 (IC_50_ = 7.1 µmol/L) cells. When introducing -OCF_3_ at the *o*-position of the A-ring and changing the *p*-position substituent of the B-ring, the compounds produced (**1c2a**-**1c2e**) showed various degrees of antiproliferative activity against cancer cells. In conclusion, when the *p*-position of the B-ring was replaced by a nitrogen atom-containing group and the *m*-position of the A-ring was substituted by -OCF_3_, the compounds showed better inhibitory activity against tumor proliferation.

Introduction of -N(CH_3_)_2_, -N(CH_2_CH_3_)_2_, or an oxygen heteroatom substituent at the *p*-position of the B-ring and replacement of the A-ring with -CH_3_ or -OCH_3_ resulted in seven compounds (**1d2a**-**1d2b**, **1e2a**-**1e2c**, and **1g2a**-**1g2b**). When introducing -CH_3_ at the *p*-position of the A-ring, the resulting compounds **1d2a** [-N(CH_3_)_2_] and **1d2b** [-N(CH_2_CH_3_)_2_] showed good antiproliferative activity against some tumor cell lines, especially **1d2b**, whose IC_50_ value against HCT116 cells was 3.1 µmol/L. Replacing the A-ring with 3,4,5-OCH_3_ and introducing -N(CH_3_)_2_ or -N(CH_2_CH_3_)_2_ at the *p*-position of the B-ring yielded **1g2a** and **1g2b**, respectively, which exerted excellent antiproliferative activity against AGS, BEL-7402, HCT116, and other cancer cell lines. Compound **1g2a** displayed the most potent antiproliferative activity against AGS (IC_50_ = 0.08 µmol/L), BEL-7402 (IC_50_ = 7.8 nmol/L), HCT116 (IC_50_ = 5.9 nmol/L), Raji (IC_50_ = 0.03 µmol/L), and SU-DHL-10 (IC_50_ = 0.03 µmol/L) cells of all the compounds tested in this study. The inhibitory effect of compound **1g2a** on the proliferation of BEL-7402, HCT116, Raji, and SU-DHL-10 cells was stronger than that of the five positive control agents used in this study, including taxol. Moreover, **1g2a** demonstrated little cytotoxicity to L-02 cells. Compound **1g2b** also exerted strong inhibition of proliferation, but it was slightly weaker in effect than **1g2a**.

Introduction of a -CF_3_ group at the para (*p*)-position of benzene ring A resulted in compounds **1f2a**-**1f2w** and **1f3a**-**1f3d**. As shown in Table **S1**, most of the compounds exhibited low antiproliferative activity. Surprisingly, however, **1f2w** (containing -CF_3_ on the side of the B benzene ring) displayed strong antiproliferative activities against Raji (IC_50_ = 48 nmol/L) and SU-DHL-10 (IC_50_ = 22 nmol/L) cells, but no antiproliferative activity against other tested cells.

On the basis of the above results, we summarize various structure-activity relationships: 1. When the A-ring was replaced by -OCF_3_, the antiproliferative activity sequence of the compounds was *m*-OCF_3_ > *o*-OCF_3_ > *p*-OCF_3_. When the meta-position of A-ring was replaced by -OCF_3,_ the anti-proliferative activity sequence of B-ring substituents was *p*-heteroatom > *p*-halogen > *p*-alkyl. 2. When the *p*-position of the A-ring was substituted with -OCH_3_ and the *p*-position of the B-ring was substituted with nitrogen-containing groups, the antiproliferative activity sequence of the compounds was *p*-N(CH_3_)_2_ > *p*-N(CH_2_CH_3_)_2_. 3. When the A-ring was replaced by 3,4,5-OCH_3_ and the *p*-position of the B-ring was substituted with nitrogenous groups, the antiproliferative activity sequence of the compounds was *p*-N(CH_3_)_2_ > *p*-N(CH_2_CH_3_)_2_.

In summary, compound **1g2a**, in which the A-ring was replaced by 3,4,5-OCH_3_ and the *p*-position of the B-ring was substituted with -N(CH_3_)_2_, exerted the most potent antiproliferative effect against many kinds of cancer cell line, but little cytotoxicity toward L-02 cells, and thus showed good and selective anticancer activity.

As shown in Table **1** and **S2**, the selectivity of compound **1g2a** for BEL-7402, HCT116, Raji, and SU-DHL-10 cells was 12,820-, 16,949-, 2,941- and 2,941-fold that for L-02 cells, respectively, much higher than that of any of the positive control agents. Therefore, it was chosen for further biological studies.

#### 3.2.2 Compound 1g2a Induces Cell Cycle Arrest with a Change in the Expression of Cyclin A, Cyclin B1, Cyclin D1 and Cyclin E1

**Fig. (4):**
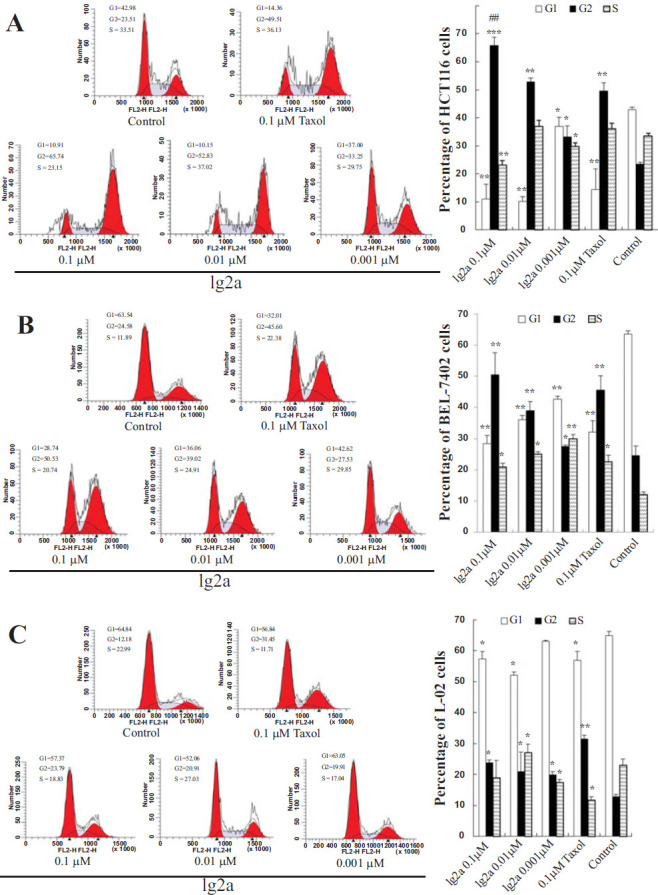
Compound **1g2a** induced G2/M arrest in HCT116 and BEL-7402 cancer cells. Cells were incubated with varying concentrations of **1g2a** (0.001, 0.01, 0.1 μM), 0.1% DMSO (control), 0.1 μM taxol and the percentage histograms of cell cycle distribution results in (**A**) HCT116 cells; (**B**) BEL-7402 cells; (**C**) L-02 cells. **p < 0.01 compared to control group; *p < 0.05 compared to control group; ^##^*p* < 0.01 compared to 0.1 μM taxol treatment group.

Cell cycle dysregulation, uncontrolled mitosis, is an important cause of proliferation of cancer cells [33]. Tubulin-destabilizing agents block the cell cycle in the G2/M phase due to microtubule depolymerization and disruption of the cytoskeleton [34]. As shown in Fig. (**4A**), **1g2a** increased the percentage of the HCT116 cell population in the G2/M phase of the cell cycle in a concentration-dependent manner from 33.25% to 65.74%, compared with cells incubated in DMSO as a vehicle control. At 0.1 μM, **1g2a** showed greater inhibition than taxol. The effects of compound **1g2a** on BEL-7402 cells were similar to those on HCT116 cells (Fig. **4B**). In L-02 normal cells, the population in the G2/M phase of the cell cycle was slightly increased in **1g2a**-treated cells compared with the vehicle group; however, from 0.001 to 0.1 μM **1g2a**, there was almost no effect on the cell cycle (Fig. **4C**). The above results showed that compound **1g2a** caused cell cycle arrest at the G2/M phase in HCT116 and BEL-7402 cells but had no significant effect on L-02 cells, which might be one of the possible mechanisms for its selective cytotoxicity.

**Fig. (5):**
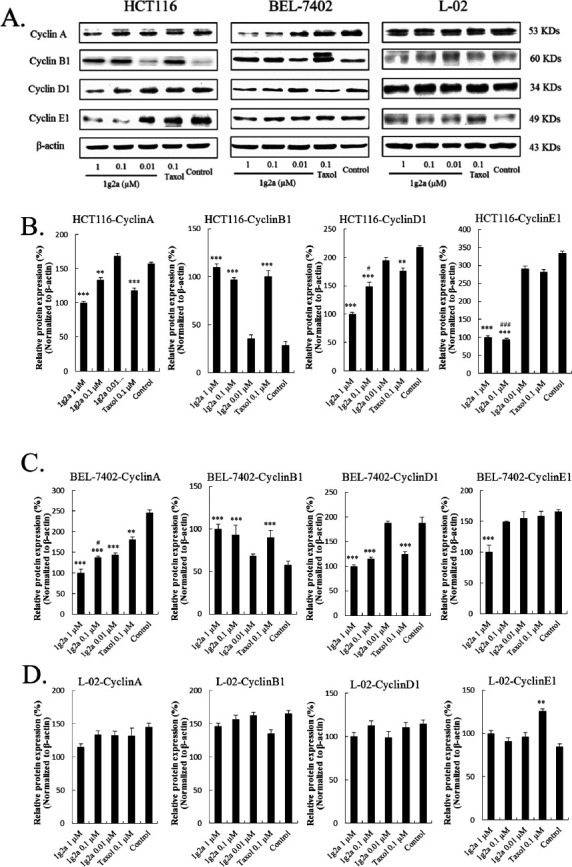
Compound **1g2a** induced G2/M arrest in HCT116 and BEL-7402 cancer cells. (**A**) Western blotting analysis on the effect of **1g2a** on the G2/M regulatory proteins. The histograms of the density ratio of cyclin A, B1, D1 and E1 to β-actin of (**B**) HCT116 cells; (**C**) BEL-7402 cells; (**D**) L-02 normal cells. Data represented as mean ± SD of independent experiments. *p < 0.05, **p < 0.01, and ***p < 0.001 compared with the control group; ^#^*p* < 0.05 and ^###^*p* < 0.001 compared to 0.1 μM taxol treatment group at same concentration treatment.

Entry to the S phase of the cell cycle requires the accumulation of cell-cycle activation-related cyclins [26, 35]. As shown in Fig. (**5A**-**C**), in HCT116 and BEL-7402 cells, the **1g2a** treatment groups had lower expression of cyclin A, cyclin D1, and cyclin E1 than the vehicle group. The reduced expression was dose-dependent. The effect on the expression of cyclin D1 and cyclin E1 by **1g2a** was much greater than that of taxol treatment at 0.1 μM; the cyclin A level by **1g2a** was similar to that of taxol treatment at 0.1 μM. Cyclin B1 plays an important role in the transition from interphase to the mitotic phase and governs cell cycle progression by enhancing cell-cycle distribution in the G2/M fraction [36]. It has been reported that expression of cyclin B1 is increased by exposure to anticancer agents in BEL-7402 cells [20]. In our experiment, **1g2a** treatment induced increased expression of cyclin B1 compared with treatment with vehicle, and cyclin B1 was upregulated in a concentration-dependent manner on **1g2a** treatment of HCT116 and BEL-7402 cells. The cyclin B1 level on **1g2a** treatment was similar to that in the taxol treatment group at 0.1 μM in both cancer cell lines. However, in the same conditions, **1g2a** hardly changed the expression of the four kinds of cyclin in normal L-02 cells (Fig. **5A** and **D**). These results suggest that the downregulation of cyclins A, D1, and E1 and the upregulation of cyclin B1 are related to the decrease in cell proliferation induced by **1g2a** treatment of HCT116 and BEL-7402 cancer cells.

#### 3.2.3 Compound 1g2a Induces Cancer Cell Apoptosis

**Fig. (6):**
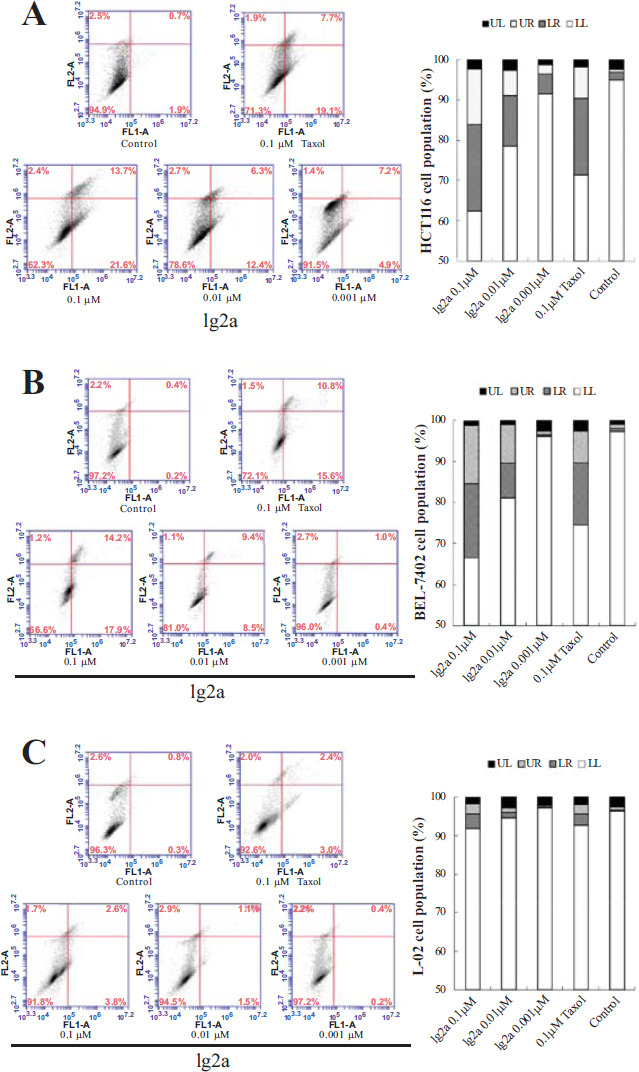
Apoptosis induction after treatment for 12 h with 0.1% DMSO (control), 0.1 μM taxol, 0.001 μM of **1g2a**, 0.01 μM of **1g2a**, 0.1 μM **1g2a**, and the total apoptosis results in (**A**) HCT116 cells; (**B**) BEL-7402 cells; (**C**) L-02 cells.

To determine whether compound **1g2a** could induce apoptosis, vehicle-, **1g2a**-, and taxol-treated HCT116 and BEL-7402 cancer cell lines and normal L-02 cells were stained with FITC-annexin V and PI. As shown in Fig. (**6A**), incubation with **1g2a** (0.001, 0.01, or 0.1 μM) for 12 h induced a concentration-dependent increase in the percentage of total apoptotic (early and late stage) HCT116 cells from 2.6% to 35.3%. Treatment with 0.1 μM **1g2a** resulted in a slightly increased number of total apoptotic cells (35.3%) compared with the taxol treatment group (26.8%), and the proportion in both was obviously higher than that in the vehicle treatment group (2.6%). The effect of compound **1g2a** on apoptosis of BEL-7402 cells was very similar to that on HCT116 cells (Fig. **6B**). However, compound **1g2a** and taxol induced almost no apoptosis in normal L-02 cells (Fig. **6C**).

#### 3.2.4 Compound 1g2a Inhibited HCT116 and BEL-7402 Cells Migration

**Fig. (7):**
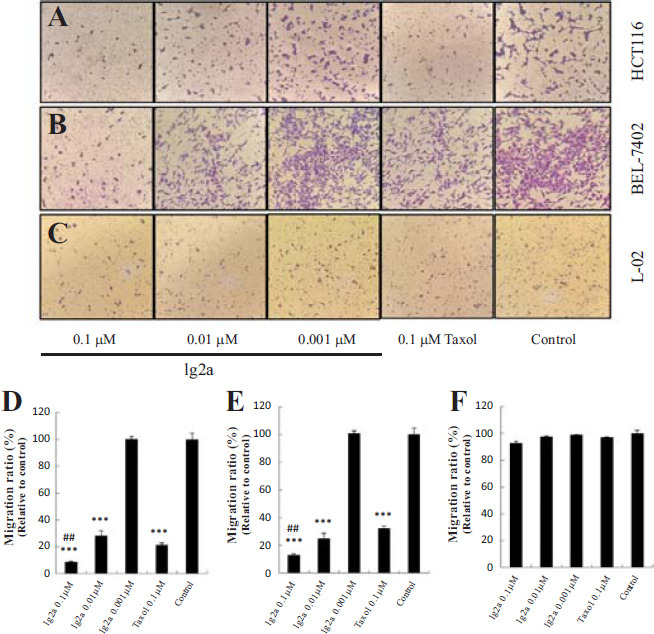
Transwell assay of compound **1g2a** showing inhibition of HCT116 and BEL-7402 cells migrations without effecting the migration of normal L-02 cells. The images of stained (**A**) HCT116, (**B**) BEL-7402, and (**C**) L-02 cells adhering in the lower layer of insert of transwell with phase-contrast microscopy (200× magnification). The relative migration ratio of (**D**) HCT116 cells; (**E**) BEL-7402 cells; (**F**) L-02 cells. Data expressed as mean±SD (n≥3). ***p < 0.001 compared to control group; ^##^*p* < 0.01 compared to 0.1 μM taxol treatment group.

Aggressive tumors have a strong ability to proliferate and migrate, and in many cases, cell mobility affects cell proliferation [2, 20]. As shown in Fig. (**7A**-**E**), compared with the control group, compound **1g2a** inhibited the migration of HCT116 and BEL-7402 cells in a concentration-dependent manner. At the same concentration of 0.1 μM, the inhibitory effect of **1g2a** on the migration of HCT116 and BEL-7402 cancer cells was significantly stronger than that of taxol. However, **1g2a** and taxol induced almost no migration in normal L-02 cells (Fig. **7C** and **F**).

#### 3.2.5 Compound 1g2a Inhibited HCT116 and BEL-7402 Cells Colony Formation

**Fig. (8):**
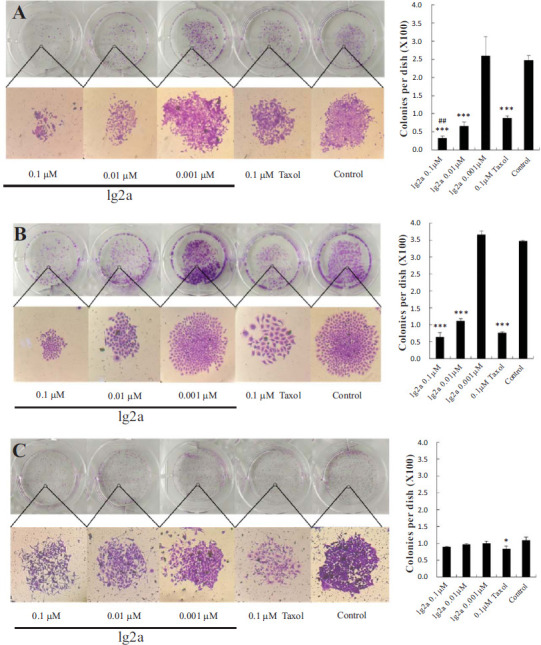
Compound **1g2a** inhibited the colony-forming ability of two cancer cells but had little effect on normal cells. (**A**) HCT116 cancer cells; (**B**) BEL-7402 cancer cells; (**C**) L-02 normal cells. Each part is divided into the images of stained colonies under phase-contrast microscopy and the statistical results of the number of colonies per dish. Values are represented as mean±SD (n≥3). *p < 0.05 and ***p < 0.001 compared to control group; ##*p* < 0.01 compared to 0.1 μM taxol treatment group.

The abnormal proliferation of cancer cells results in malignant tumor. Colony formation assay directly shows the proliferation ability of a single cancer cell to turn into a cell colony *in vitro* [20, 37]. As shown in Fig. (**8A**-**B**), compared with the control group, with the increase of **1g2a** incubation concentration (0.001-0.1 µM), the concentration dependence of HCT116 and BEL-7402 cells on colony formation decreased. At the same concentration of 0.1 µM, the colony-forming ability of **1g2a** on HCT116 cancer cells was significantly stronger than that of taxol. However, **1g2a** expressed little effect on the colony formation of normal L-02 cells (Fig. **8C**).

#### 3.2.6 Compound 1g2a Inhibited HCT116 and BEL-7402 Cells Tubulin Polymerization

Compound **1g2a** and taxol, as a positive control, were tested for their ability to inhibit tubulin polymerization at 1, 0.1 and 0.01 µM concentrations using ELISA assay for β-tubulin (Table **2**). Compound **1g2a** (56.6%; 68.38%) showed significant ability to inhibit tubulin polymerization compared to the taxol (29.58%; 46.04%) on BEL-7402 and HCT116 cells at 1 µM concentration, respectively, and also (49.86%) better than that of taxol (20.08%) at 0.1 µM concentration on BEL-7402 cells. The above results indicated that **1g2a** can selectively inhibit the colony formation of cancer cells.

#### 3.2.7 Compound 1g2a Tightly Bound to Tubulin

**Fig. (9):**
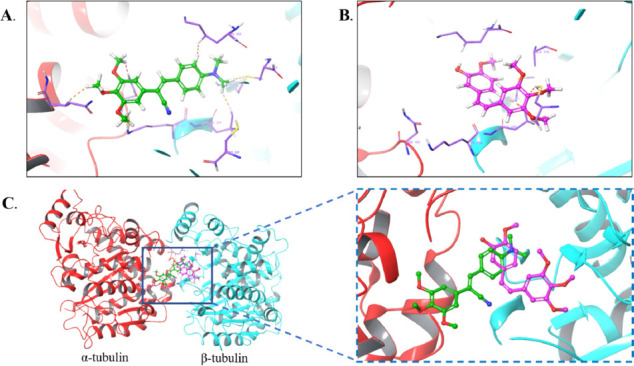
Molecular modeling of compounds in complex with tubulin (PDB: 1SA0). Shown is the proposed binding mode and interaction between tubulin and selected compounds. (**A**) **1g2a**, (**B**) CA-4. (**C**) Superposition of **1g2a** and CA-4 in the tubulin. The compounds are shown in a stick model with carbon atoms in green (**1g2a**) and pink-red (CA-4).

Since compound **1g2a** showed potent anticancer activity, we further investigated the binding pattern of **1g2a** to tubulin (PDB code: 1SA0) [38]. CA-4 was used as a positive control. The docking results are shown in Fig. (**9**). Compound **1g2a** interacted with several amino acid residues of the tubulin colchicine binding site to form abundant chemical bonds, including Glu183, Gly143, Gly144, Gln11, and Glu71 on the α chain of tubulin and Leu248 and Thr353 on the β chain of tubulin (Fig. **9A**). In the case of CA-4, the diagram effectively displays these structural features, highlighting several binding regions on the β subunit but not with the α subunit (Fig. **9B**-**C**). When the **1g2a** and CA-4 structures were simultaneously docked to the tubulin colchicine binding site, we found that the structures of **1g2a** were in the middle of the tubulin α chain and β chain. However, CA-4 showed different results (Fig. **9C**), which may account for why **1g2a** showed strong anticancer activity and a high selective index [19].

## CONCLUSION

In this study, 83 novel small molecules with 2- phenylacrylonitrile were designed, synthesized, and biologically evaluated. Many of them displayed significantly enhanced antiproliferative efficacy against a panel of cancer cell lines. Among these molecules, compound **1g2a**, with 3,4,5-OCH_3_ introduced on the A-ring and an -N(CH_3_)_2_ group at the *p*-position of the B-ring, exerted the most potent antiproliferative activity on many kinds of cancer cell line, but showed little cytotoxicity toward L-02 normal cells (*i.e.*, it showed selective anticancer activity). The SI of **1g2a** for BEL-7402, HCT116, Raji, and SU-DHL-10 cells compared with normal L-02 cells was 12,820, 16,949, 2,941, and 2,941, respectively, much higher than the values for CA-4, resveratrol, colchicine, and taxol. Compound **1g2a** arrested BEL-7402 and HCT116 cells in the G2/M phase of the cell cycle and induced apoptosis in a dose-dependent manner. Mechanistic studies suggested that the blockage in the G2/M phase was associated with the downregulation of cyclin A, D1, and E1 and the upregulation of cyclin B1. Molecular modeling suggested that the selectivity of **1g2a** to inhibit only cancer cell proliferation was achieved by penetrating deep into the hydrophobic pocket of tubulin and binding between the α- and β-subunits of tubulin. Compound **1g2a** inhibited the migration levels of BEL-7402 and HCT116 cells to reduce the colony formation abilities of BEL-7402 and HCT116 cells in a concentration-dependent manner and significantly inhibited β-tubulin polymerization of HCT116 and BEL-7402 cells.

Furthermore, *in vivo*, compound **1g2a** significantly suppressed tumor volume and reduced its weights by 66% (♀) and 78% (♂) at a dose of 25 mg/kg/day (iv) in an HCT116 colon cancer xenograft model in mice without affecting the mouse body weight (Fig. **S3**). Although taxol also inhibited the growth of tumors in the same treatment conditions, the body weight of the mice decreased significantly. Moreover, through a molecular ADMET forecast study, it was found that **1g2a** is water soluble, shows good intestinal absorption, has no inhibitory effect on CYP2D6, and has good PPB possibility (Table **S3**).

In summary, the novel tubulin inhibitor, compound **1g2a**, showed outstanding antitumor activity both *in vivo* and *in vitro* and has the potential to be developed into a promising effective anticancer agent with little toxicity to normal tissues.

## Figures and Tables

**Table 1:** *In vitro* anti-proliferative activity of the synthesized compounds, CA-4, CA-4P, colchicine, resveratrol and taxol against thirteen cell lines^a^ (IC_50_ (µM^b^)).

**IC_50 _(μM)**														
	**Agent**	**A549**	**AGS**	**BEL-7402**	**HCT-116**	**HeLa**	**HepG-2**	**L-02**	**MGC-803**	**MCF-7**	**MCF-10A**	**Raji**	**SGC-7901**	**SU-DHL-10**
1	**1a2a**	>100	>100	>100	>100	>100	>100	>100	>100	>100	>100	>100	>100	>100
2	**1a2b**	>100	13.2±3.3	15.0±2.7	23.5±4.6	35.9±.2.5	>100	>100	37.4±5.8	>100	>100	49.0±7.4	>100	38.8±3.7
3	**1a2c**	65.8±8.9	18.6±3.0	>100	20.2±1.7	72.3±2.4	20.7±1.4	>100	35.2±2.8	55.2±2.8	>100	55.1±3.2	56.4±9.7	>100
4	**1a2d**	>100	19.6±2.9	>100	25.4±6.8	87.3±4.1	24.3±3.4	>100	>100	>100	>100	64.9±1.5	>100	73.5±17.3
5	**1a2e**	>100	30.8±7.9	>100	>100	>100	>100	>100	>100	>100	>100	>100	>100	>100
6	**1a2f**	>100	14.6±5.9	>100	43.5±3.8	>100	>100	>100	56.7±11.0	59.9±2.3	>100	42.7±3.4	77.9±12.6	15.1±11.6
7	**1a2g**	71.6±24.7	8.7±2.1	>100	57.4±7.8	>100	>100	>100	>100	>100	>100	66.6±13.3	89.9±5.6	27.3±5.3
8	**1a2h**	>100	69.6±15.7	24.1±2.8	8.7±1.7	>100	>100	>100	16.8±0.7	>100	>100	67.4±31.7	19.7±1.5	>100
9	**1a2i**	>100	>100	>100	>100	>100	>100	>100	>100	>100	>100	45.1±15.6	>100	>100
10	**1b2a**	>100	>100	>100	>100	>100	>100	>100	>100	>100	>100	>100	>100	>100
11	**1b2b**	>100	8.2±2.6	3.0±1.0	1.1±0.3	>100	15.9±5.2	16.1±1.0	1.2±0.6	>100	11.3±2.3	17.0±4.3	5.4±0.2	60.4±0.7
12	**1b2c**	>100	>100	>100	>100	>100	>100	>100	>100	>100	>100	>100	>100	>100
13	**1b2d**	>100	13.9±4.2	14.6±4.6	15.1±2.9	66.8±9.8	34.9±3.2	50.4±0.8	10.1±0.9	>100	34.1±3.6	16.3±6.1	21.9±3.3	35.6±0.9
14	**1b2e**	>100	4.5±2.0	39.4±6.1	25.0±0.5	61.7±3.3	38.2±0.1	54.2±12.3	11.1±0.3	>100	>100	43.1±4.5	8.1±2.9	29.3±2.0
15	**1b2f**	>100	43.8±21.8	44.8±10.6	>100	>100	72.9±9.9	>100	49.3±0.3	>100	>100	>100	61.7±0.4	>100
16	**1b2g**	>100	>100	>100	>100	>100	>100	>100	>100	>100	>100	>100	>100	>100
17	**1b2h**	>100	>100	1.0±0.8	0.3±0.2	>100	42.3±18.2	52.0±8.4	3.7±1.3	>100	>100	20.1±2.3	7.1±2.4	>100
18	**1b2i**	>100	23.9±2.7	13.0±1.1	12.3±1.6	>100	23.5±4.3	28.5±0.8	10.8±2.9	>100	79.6±5.7	23.7±2.1	46.0±4.9	53.6±7.5
19	**1b3a**	32.6±1.1	15.4±0.5	>100	19.3±0.1	38.8±3.5	21.5±7.6	35.9±1.6	20.7±1.5	25.5±0.4	22.7±3.5	56.6±5.8	20.9±5.1	59.7±0.8
20	**1c2a**	>100	>100	>100	>100	>100	>100	>100	>100	>100	>100	>100	>100	>100
21	**1c2b**	>100	31.4±8.3	>100	>100	>100	70.5±25.4	>100	29.0±8.3	>100	>100	>100	>100	>100
22	**1c2c**	>100	16.7±2.3	>100	17.2±5.2	>100	27.9±2.4	42.2±11.5	19.6±6.7	>100	>100	42.4±1.7	>100	>100
23	**1c2d**	50.7±7.4	6.3±0.4	17.4±4.2	>100	>100	26.7±10.1	>100	13.1±3.6	>100	>100	60.5±19.1	50.1±5.9	>100
24	**1c2e**	>100	7.6±0.6	10.8±2.3	8.4±2.4	75.8±12.4	24.9±7.0	36.1±4.8	2.5±1.1	>100	>100	41.3±5.5	7.8±0.8	>100
25	**1d2a**	>100	23.0±1.0	>100	>100	>100	>100	>100	18.8±1.7	>100	>100	7.9±0.5	>100	19.9±0.7
26	**1d2b**	>100	24.1±0.2	65.4±2.5	3.1±0.5	>100	>100	>100	8.6±0.04	52.3±2.2	>100	11.8±0.4	>100	42.6±11.4
27	**1e2a**	>100	18.4±1.9	46.8±5.1	7.2±0.6	>100	>100	47.5±12.4	8.3±1.2	36.9±3.6	28.1±3.6	11.6±4.9	38.2±9.0	27.2±5.3
28	**1f2a**	>100	>100	>100	>100	>100	>100	>100	>100	>100	>100	>100	>100	>100
29	**1g2a**	>100	0.08±0.05	0.007±0.001	0.005±0.001	>100	>100	>100	14.0±8.4	0.27±0.04	0.02±0.001	0.03±0.001	>100	0.03±0.01
		-	> 1250^c^	> 12820^c^	> 16949^c^	-	-		> 7.1^c^	-		> 2941^c^	-	> 2941^c^
30	**1g2b**	>100	7.5±0.4	0.03±0.01	1.0±0.1	>100	>100	>100	0.12±0.03	0.45±0.06	0.05±0.001	0.37±0.06	>100	2.4±0.1
31	**8a**	>100	>100	13.2±2.7	56.3±8.4	>100	>100	>100	>100	17.6±6.5	11.3±2.3	1.2±0.5	>100	1.3±0.1
	CA-4	>100	3.4±0.01	2.0±0.03	0.16±0.001	>100	>100	1.1±0.03	0.06±0.02	0.2±0.1	3.2±1.2	0.18±0.001	0.30±0.001	1.1±0.1
		-	-	-	> 6.8^c^	-	-		> 18.3^c^	> 14.6^d^		> 5.8^c^	> 3.6^c^	-
	CA-4P	>100	4.7±0.2	5.4±2.0	0.5±0.2	>100	>100	2.6±0.6	0.03±0.02	0.4±0.2	14.5±1.3	0.42±0.01	0.1±0.03	0.88±0.07
		-	-	-	> 4.5^c^	-	-		> 89^c^	> 30.3^d^		> 6.2^c^	> 22.2^c^	> 3.0^c^
	Taxol	0.4±0.02	0.02±0.01	0.09±0.02	0.03±0.01	12.9±0.4	0.9±0.03	>100	0.06±0.01	0.08±0.01	0.18±0.02	0.26±0.02	>100	0.18±0.03
		> 227^c^	> 5000^c^	> 1111^c^	> 3333^c^	> 7.7^c^	> 106^c^		> 16666^c^	> 2.2^d^		> 384^c^	-	> 555^c^
	Colchicine	6.5±1.5	0.1±0.03	0.02±0.001	0.01±0.001	1.8±0.1	1.7±0.4	0.4±0.02	0.13±0.01	0.6±0.3	1.4±0.3	0.3±0.1	0.1±0.04	0.29±0.001
		-	-	> 16.7^c^	> 29.3^c^	-	-		> 3.6^c^	> 2.3^d^		> 1.3^c^	> 4.2^c^	> 1.6^c^
	Resveratrol	>100	>100	42.5±5.4	16.7±3.6	>100	>100	12.8±0.2	>100	72.4±9.9	>100	>100	>100	>100

**Note:**^a^Cytotoxicity, as IC_50_ for each cell line, refers to the concentration of compound which reduced by 50% the optical density of treated cells with respect to untreated cells using the MTT assay.

^b^Data represent the mean values of three independent determinations.

^c^SI (selectivity index), IC_50 (L-02) _*/ *IC_50 (cancer cell)_

^d^SI (selectivity index), IC_50 (MCF-10A) _*/ *IC_50 (MCF-7)_

-: not suitable for calculation

**Table 2:** Compound 1g2a inhibits the polymerization of β-microtubulin.

**Cpd. No.**	**IC_50_** **±SD (µM)**	**Tubulin % inhibition** ^a^	**IC_50_** **±SD (µM)**	**Tubulin % inhibition** ^a^
	**BEL-7402**		**HCT116**	
**1g2a **(1µM)	0.007±0.001	56.6±5.46***, ^##^	0.005±0.001	68.38±0.03***, ^##^
**1g2a **(0.1µM)		49.86±6.66***, ^##^		26.11±1.02***, ^#^
**1g2a **(0.01µM)		18.38±5.90*		26.02±0.29***
Taxol (1µM)	0.09±0.02	29.58±4.88**	0.03±0.01	46.04±4.89***
Taxol (0.1µM)		20.08±5.34**		36.45±6.07***
Taxol (0.01µM)		14.96±2.65**		19.07±6.27**
Vehicle		0		0

**Note: **^a ^tubulin inhibition of BEL-7402, HCT116 cells treated with compound **1g2a** and taxol compared to control untreated cells.

***p < 0.001 compared to control group; **p < 0.01 compared to control group; *p < 0.05 compared to control group.

^##^*p* < 0.01 compared to 1 μM taxol treatment group; ^#^*p* < 0.05 compared to 0.1 μM taxol treatment group.

## Data Availability

The data and supportive information are available within the article.

## LIST OF ABBREVIATIONS

DS: Discovery Studio
CA-4: Combret Astatin A-4
