# Supplementary material for: Synthesis and Selective Anticancer Activity Evaluation of 2-phenylacrylonitrile Derivatives as Tubulin Inhibitors
Source: Curr Med Chem. 2024 Feb 26;31(15):2090–106. doi: 10.2174/0109298673263854231009063053 (PMC11071649; doi:10.2174/0109298673263854231009063053)
Supplement: Supplementary file 1 — Supplementary material is available on the publisher’s website along with the published article. [file CMC-31-2090_SD1.pdf]

Supplementary Material

Synthesis and Selective Anticancer Activity Evaluation of 2-phenylacrylonitrile Derivatives as Tubulin Inhibitors

Ye-Zhi Jin[1,#], Ya-Bing Xin[1,#], Yuan Li[1,#], Xin-Yuan Chen[1,#], De-Ao Man[1,#] and Yu-Shun Tian[1,*]

[1]*Key Laboratory of Natural Medicines of the Changbai Mountain, Department of Medicinal Chemistry, Ministry of Education, College of Pharmacy, Yanbian University, Yanji, Jilin Province 133002, PR China*

1. EXPERIMENTAL SECTION

1.1. Reagents and instruments

All chemicals and spectral grade solvents were obtained commercially and were used without further purification. Reactions were monitored by thin-layer chromatography (TLC) and carried out on Merck Kieselgel 60 F254 plates, which could be visualized under UV light at 254 nm. ^{1}H NMR and ^{13}C NMR spectra were obtained on an AV-300 (Bruker, Switzerland), making a solution of samples in $CDCl_3$ and DMSO-d_6 solvent using tetramethyl silane (TMS) as the internal standard. The chemical shifts are given in ppm referenced to the respective solvent peak, and coupling constants (J) are reported in hertz (Hz). Multiplicity is denoted as s (singlet), d (doublet), t (triplet), q (quartet), m (multiplet) and br (broad). The NMR data was processed by software Mest Re-Nova. High-resolution mass spectra were measured on a MALDI-TOF/TOF mass spectrometer (Bruker Dartonik, Germany). Melting points of target compounds were determined in the open glass capillaries and were uncorrected.

1.2. General procedure for the synthesis of compounds 1a2a-u, 1b2a-k, 1c2a-e, 1d2a-b, 1e2a-c, 1f2a-w, 1g2a-b, 1a3a-c, 1b3a and 1f3a-d[1-2]

4-Trifluoromethylbenzoic acid, 4-trifluoromethoxybenzoic acid, 3-trifluoromethoxybenzoic acid, 2-trifluoromethoxybenzoic acid, 4-methylbenzoic acid, 4-methoxybenzoic acid and 3,4,5-trimethoxybenzoic acid were used as starting materials, respectively. The carboxyl group of each benzoic acid was esterified to form corresponding ester, and the corresponding bromide was produced through reduction and bromination. Then, a mixture of 10 mmol of each of the above bromide, 1.48 g (15.0 mmol) of TMSCN, 4.72 g (15.0 mmol) of TBAF, and 30 mL of acetonitrile was refluxed for 4-6 h with stirring. The reflux process was monitored by TLC. The reaction mixture was cooled to room temperature (RT) and then was added to 200 mL of ice water with severe stirring. The mixture was filtered, washed with 50% methanol, and purified by recrystallisation from 95% ethanol, yielding the intermediates **1a-g**.

A mixture of 1.0 mmol of a compound from **1a-g**, 1.0 mmol properly substituted benzaldehyde or aromatic heterocyclic aldehyde, and 10 mL of methanol was heated to 60°C with stirring. After 30 min, sodium methoxide (0.027 g, 0.50 mmol) was added to the mixture and the mixture was kept at 60°C for 4-6 h. The reaction was monitored by TLC. Once complete, the reaction mixture was cooled to RT, and the precipitate was separated by filtration, recrystallised from methanol, or purified by silica gel column chromatography to give the target compound (Scheme S1).

1.3. General procedure for the preparation of compounds 7a-c

According to the literature[3-4], substituted anilines (10 mmol) were diazotized by $NaNO_2$ (15 mmol) to form diazonium salts, which were subsequently converted into azides. Then, the azides were put directly in the next step without purification to prevent degradation. The synthesis of triazoles involved the 1,3-dipolar cycloaddition reaction between propargyl alcohol (1 mmol) and aromatic azides (1 mmol), which was catalyzed by $CuSO_4$ pentahydrate (0.05 mmol) and sodium ascorbate (0.1 mmol), guided the regioselectivity to produce 1,4-disubstituted 1,2,3-triazole alcohols. After purification in a flash column, 1,2,3-triazole alcohol (20 mmol) was further oxidized to aldehyde using IBX (22 mmol) in DMSO (25 mL). Then, H_2O (20 mL) was added to precipitate IBX crystals, and these crystals were decanted. The mother liquor was extracted with ethyl acetate, washed with $NaHCO_3$ solution, and dried over anhydrous $MgSO_4$ to obtain series **4** as white or light-yellow crystals in yields ranging from 85% to 100% (Scheme S2). And then reacted with 2-(4-(trifluoromethoxy)phenyl)acetonitrile to obtain the final target compounds **7a-c** using the procedure as described in **1.2** (Scheme S1).

1.4. General procedure for the preparation of compounds 8a-d

Chalcones were synthesized, adopting a reference reported method[5] and described as follows: Formylbenzoic aldehyde (10.0 mmol) and the appropriate aryl ketone (10.3 mmol) were successively added to EtOH (60 mL) upon stirring at RT. KOH (1 M, 20 mL) was added and stirring was continued. The progress of the reaction was followed by TLC. After completion, the

pH of the solution was adjusted to 2 with HCl solution (1 M), and the precipitate was subsequently collected by suction filtration and washed with water, dried to yield the desired pure compound series **5** (Scheme S2). Then reacted with 2-(4-(trifluoromethoxy)phenyl)acetonitrile to obtain the final target compounds **8a-d** using the procedure as described in **1.2** (Scheme S1).

1.5. Procedure for the preparation of compound 9

Compound 1*H*-indole (35 mmol) in DMF (40 mL) was added to a stirred and completely mixed solution of $POCl_3$ (20 mL) in DMF (40 mL) at 0°C. After 2 h reaction, the mixture was poured into 300 mL ice water and mixed completely. Then adjust the pH value to 9 with NaOH and extracted with ethyl acetate and dry to obtain crude compounds 1*H*-indole-3-carbaldehyde. NaH (62.5 mmol) in THF (15 mL) was added slowly to a stirred solution of 1*H*-indole-3-carbaldehyde (25 mmol) in THF (15 mL) at 0°C, then after 15 min stirring, benzyl chloride (33 mmol) was added. The reaction mixture was moved to RT and allowed to continue, after which it was deemed essentially complete by TLC. Then the solution was removed and the mixture was extracted by ethyl acetate to obtain crude compound **6** (Scheme S2) and then, reacted with 2-(4-(trifluoromethoxy)phenyl)acetonitrile to obtain the final target compound **9** using the procedure as described in **1.2** (Scheme S1).

1.6. Cell lines and cell culture

MTT was purchased from Sigma-Aldrich Co. (St. Louis, MO, USA). The PI and Annexin V-FITC apoptosis detection kit were purchased from Invitrogen (Eugene, OR, USA). All human cell lines were used in this study. MGC-803 late stage differentiation gastric cancer cell, A549 lung cancer cell, HepG2 hepatoma cell, AGS gastric cancer cell, BEL-7402 liver cancer cell, HCT116 colectal cancer cell, HeLa cervical cancer cell, SGC-7901 early differentiation gastric cancer cell, L-02 normal liver cell, MCF-7 breast cancer cell, MCF-10A normal breast cell, Raji B lymphoblastoid *cell* and diffuse large B-cell lymphoma SU-DHL-10 cell were initially purchased from American Type Culture Collection (ATCC, Manassas, Virginia). RPMI-1640 media, DMEM, and FBS were provided from Gibco Company. The cells were maintained in DMEM or RPMI-1640, supplemented with 10% FBS, 100 IU/mL penicillin and 100 mg/mL streptomycin, and at 37°C in a humidified atmosphere containing 5% CO_2.

1.7. Cell growth inhibition assay (MTT assay)

The experiment was carried out according to the method of reference[2]. Briefly, cells were plated in 96-well plates at a density of 30-40% for 1 day, and the adherent cells were treated with concentrations of compounds (0.001~100 μM) that do not have solubility problem. Taxol, colchicine, resveratrol, CA-4 and CA-4P were used as positive controls. After 48 h incubation, the anti-proliferative activities were determined by MTT assay.

1.8. Analysis for cell cycle by flow cytometry

According to the reference[1, 6], using HCT116, BEL-7402 and L-02 cells to did the analysis. Briefly, after synchronization at the G0 phase by serum starvation for 24 h, the cells were incubated with 0.001, 0.01, 0.1 μM of **1g2a** and 0.1 μM of taxol, respectively. Vehicle group was treated with 0.1% DMSO. After 12 h, the cell detached, fixed, and analyzed the cell cycle by flow cytometry using a FACS Calibur flow cytometer with Cell Quest software (Becton-Dickinson, Franklin Lakes, NJ).

1.9. Analysis of apoptosis by flow cytometry

Apoptosis was detected using an apoptosis detection kit. HCT116, BEL-7402 and L-02 cells were plated in 6-well plates (5.0×10^5 cells/well) and incubated at 37°C overnight. Exponentially growing cells were then incubated with **1g2a** at either 0.001, 0.01, 0.1 μM, or taxol at 0.1 μM. Vehicle group was treated with 0.1% DMSO. Following 12 h of incubation, cells were analyzed for apoptosis as previously described method[2,6], using a FACS Calibur flow cytometer with Cell Quest software (Becton-Dickinson, Franklin Lakes, NJ).

1.10. Cell migration assay

Quantitative cell migration was performed using 24-well Boyden chambers (Corning, NY, USA) as described[2]. Briefly, transwells with 8 μm pore size filters were inserted into 24-well plates. Medium (500 μL) containing 10% FBS was added to the lower chamber, and 100 μL of a serum-free cell suspension (1×10^5 cells) was placed in the upper chamber. The HCT116, BEL-7402 and L-02 cells were pre-treated with indicated concentrations of **1g2a**, taxol, or DMSO, and after 24 h, the results of migration were obtained.

1.11. Colony formation assay

As previously described colony formation assay[2], 1×10^4 HCT116, BEL-7402 and L-02 cells/well were seeded into 6-well plates and then stimulated with the indicated concentration of **1g2a**, taxol, or DMSO. The plates were removed from incubation when colonies were large enough to count (>50 cells). Colonies were then fixed and stained with 1×Giemsa solution, rinsed, allowed to dry, and counted. All the experiments were repeated independently at least three times.

1.12. Western blotting

According to the reference, using HCT116, BEL-7402 and L-02 cells to did immunoblotting assay[7-8]. Protein concentration was determined by BCA Protein Assay Kit (Beyotime, China) at 570 nm. The proteins, which transferred to a PVDF Hybond-P membrane (Millipore, Billerica, MS, USA), were detected by electrochemiluminescence (Bio-Rad, CA, USA).

1.13. Tubulin polymerization inhibitory activity

In vitro kinetics of microtubule assembly was measured using ELISA kit for Human β-tubulin (TUBB) on BEL-7402, HCT-116 cell lines[9]. Compound **1g2a** (0.01, 0.1, 1 μM) and taxol (0.01, 0.1, 1 μM) were used for the test.

Put about 500,000 cells in a 6 cm culture dish and left the cells to grow to 70%-80%. Aspirated the medium, added 1.5 mL of medium containing 1.5 μL of drug and then were incubated at 37°C for 24 h. Aspirated away the medium, washed with PBS, trypsinized the cells and centrifuged, diluted the cell suspension with PBS (PH 7.2-7.4), counted the 1 million cells, centrifuged and removed the supernatant. Cell pellets were lysed in 0.15 mL of ice-cold lysis buffer. Centrifuged for about 15 minutes (3000 rpm). The supernatant was carefully collected. If precipitate forms during storage, centrifuge again.

On the enzyme coated plate, added 50 μL of standard to the enzyme plate. Added 40 μL of sample dilution to the sample wells, then added 10 μL of the sample to be measured. Added 100 μL of horseradish peroxidase (HRP) enzyme reagent to each well, except for blank wells. Incubated at 37°C for 60 minutes. Five washings were done, then added 50 μL of detection reagent A and then 50 μL of detection reagent B was added, and incubation was continued at 37°C for 15 min. Stop solution was added in 50 μL. Optical density (O.D.) was measured at 450 nm.

1.14. *In vivo* antitumor activity

All surgical procedures and application on the animals were in accordance with IACUC guidelines. *In vivo* antitumor activity was performed as described[10]. The male and female athymic Nu/Nu nude mice (24$\pm$2 g, Vital River, Beijing, China) were housed under pathogen-free conditions. HCT116 cells suspension (6×10^6 cells in 0.1 mL saline) were injected into the right back of mice. Two weeks after injection, the mice bearing tumors (an average size of 40 mm^3) were distributed into 5 groups (n = 6 mice per group, half female and half male) at random. The groups with 12.5, 25.0, and 50.0 mg/kg of **1g2a**, 25.0 mg/kg of taxol were administered intraperitoneal (i.p.) injection in a vehicle of 1% DMSO/10% glycerol/10% absolute ethanol/79% saline, respectively. The negative control group was treated with the same vehicle. At the same time, toxicity test by **1g2a** (50.0 mg/kg) was carried out on six mice (half female and half male, not inoculated with tumor cells). Treatment was done at a frequency of i.p. injection one dose per day for a total twenty consecutive days. On day 21, the solid tumors were harvested from sacrificed tumor-bearing mice, and the tumors were excised, weighted, and calculated the sizes and volumes.

1.15. Molecular modeling

The molecular model and protein structure were processed using the Computer-Assisted Drug Design software Discovery studio 2017Server. The ligand and protein were prepared, hydrogen was added and water molecules were deleted by DS Server[1-2]. In this study, the structures of tubulin (PDB code: 1SA0) downloaded from protein database. The 3D structures of compounds were provided using Chem 3D, and Dock Ligand (LibDock) was used to perform the docking. The output poses of the ligands generated were analyzed based on the LibDockScore function.

1.16. Prediction of drug metabolism and toxicity

The molecular ADMET forecast study was performed using Discovery Studio (DS) 2017 Client. In this study, the compound structure and the ADMET of the commercially available comparator in the body were simulated by computer. The 3D structures of compounds were provided using Chem 3D. The analysis command is executed on DS2017, and the ADMET analysis result is generated after the operation.

1.17. Statistical analysis

Each experimental data point represents the mean±SD of at least three independent experiments. Statistical analyses were performed with a Student's *t*-test. A p value < 0.05 was considered statistically significant. The p value < 0.01 and < 0.001 were considered extremely significant.

1.18. In vivo, compound 1g2a inhibited xenograft tumor growth without affecting mouse body weight.

To evaluate the antitumor activity of compound **1g2a** *in vivo*, a colon cancer xenograft model was established by subcutaneous inoculation of HCT116 cells into SPF Nu/Nu nude mice. The mice were randomly assigned to treatment groups (three male and three female animals per group): one control group; 12.5, 25, and 50 mg/kg of **1g2a**; 25 mg/kg taxol; and a toxicity test group (no inoculation of tumor cells, animals treated with 50 mg/kg of **1g2a**). As shown Figure S3A and B, from day 10 after beginning drug treatment, the average volume and weight of the tumor in the three groups treated with **1g2a** (12.5, 25, and 50 mg/kg) and the group treated with taxol were significantly lower than those in the control group. Compound **1g2a** inhibited the growth of the tumor in a concentration-dependent manner (Figure S3A–F). The tumor growth inhibiton rate in female mice

treated with 12.5, 25, and 50 mg/kg of **1g2a** and 25 mg/kg taxol was 36%, 66%, 75%, and 82%, and in male mice the growth inhibiton rate was 66%, 78%, 85%, and 89%, respectively (Figure S3E-F). At the same drug concentration, the inhibitory effect of **1g2a** on tumor weight was greater in male mice than in female mice. No significant difference was found in the body weight of **1g2a-**treated mice compared with controls, although from the eighth day of taxol treatment, the body weight of the male mice was significantly reduced compared with the control group (Figure S3G and H). There was no change in body weight in the toxicity test group compared with the control group, indicating that the dose of **1g2a** used in the experiment was within the safe range for mice (Figure S3G and H). On the basis of the above data, the *in vivo* antitumor activity of **1g2a** was significantly greater than that of vehicle and slightly less than that of the positive control taxol. However, the toxicity of taxol to the animals was significantly higher than that of **1g2a**.

1.19. Compound 1g2a had good ADMET prediction results.

Since pharmacokinetics and drug toxicity is major reasons of drug attrition, predicting those parameters is useful to reduce the cost of drug discovery. We performed a pharmacokinetic simulation for some of the synthesized compounds. Taxol, CA-4, colchicine and resveratrol were used as the positive controls (Table S3). The results indicated that **1g2a** had good ADMET (absorption, distribution, metabolism, excretion, and toxicity) prediction results, such as water soluble, low central toxicity, good intestinal absorption, no inhibitory effect on CYP2D6 enzyme, and acceptable level of drug plasma protein binding (PPB) ability.

IDENTIFICATION DATA OF COMPOUNDS

2.1. (Z)-3-phenyl-2-(4-(trifluoromethoxy)phenyl)acrylonitrile (1a2a)

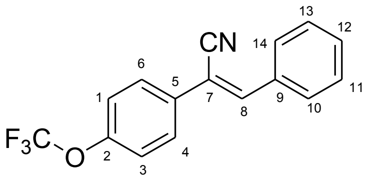

White powder; yield 31%. M.p.98-100°C. ^{1}H NMR (500 MHz, CDCl$_3$) δ 7.89 (d, *J* = 6.8 Hz, 2H, C4, C6-H), 7.71 (d, *J* = 7.2 Hz, 2H, C10, C14-H), 7.52 (s, 2H, C11, C13-H), 7.49 (s, 1H, C8-H), 7.48 (s, 1H, C12-H), 7.30 (d, *J* = 8.1 Hz, 2H, C1, C3-H). ^{13}C NMR (75 MHz, CDCl$_3$) δ 149.71 (s, C2), 143.06 (s, C8), 134.46 (s, C12), 133.39 (s, C9), 133.16 (s, C5), 130.90 (s, C1, C3), 129.34 (s, C11, C13), 129.06 (s, C10, C14), 127.56(s, C4, C6, 121.42 (s, C2-OCF$_3$), 117.67(s, C7-CN), 110.32 (s, C7). HRMS (ESI) m/z calcd for $C_{16}H_{10}F_3NO$ ($[M]^-$) 289.07090, found 289.07013.

2.2. (Z)-3-(4-fluorophenyl)-2-(4-(trifluoromethoxy)phenyl)acrylonitrile (1a2b)

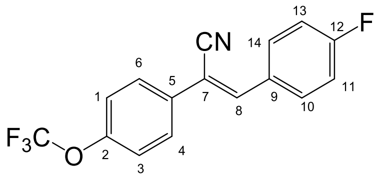

White powder; yield 31%. M.p.98-100°C. ^{1}H NMR (300 MHz, CDCl$_3$) δ 7.93 (dd, *J* = 8.8, 5.3 Hz, 2H, C10, C14-H), 7.77 – 7.67 (m, 2H, C11, C13-H), 7.50 (s, 1H, C8-H), 7.32 (d, *J* = 8.6 Hz, 2H, C4, C6-H), 7.20 (t, *J* = 8.6 Hz, 2H, C1, C3-H). ^{13}C NMR (75 MHz, CDCl$_3$) δ 146.56 (s, C2), 141.65 (s, C8), 136.23 (s, C12), 132.97 (s, C9), 131.55 (s, C10, C14), 131.43 (s, C5), 127.51 (s, C11, C13), 121.46 (s, C4, C6), 117.58 (s, C1, C3), 116.45 (s, C2-OCF$_3$), 116.17 (s, C7-CN), 110.08 (s, C7). HRMS (ESI) m/z calcd for $C_{16}H_9F_4NO$ ($[M+H]^+$) 308.06930, found 308.06891.

2.3. (Z)-3-(4-chlorophenyl)-2-(4-(trifluoromethoxy)phenyl)acrylonitrile (1a2c)

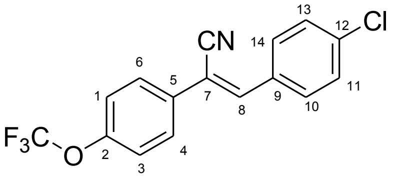

White powder; yield 36%. M.p.78-80°C. ^{1}H NMR (300 MHz, CDCl$_3$) δ 7.85 (d, *J* = 8.6 Hz, 2H, C10, C14-H), 7.78 – 7.70 (m, 2H, C11, C13-H), 7.49 (d, *J* = 1.9 Hz, 2H, C4, C6-H), 7.46 (s, 1H, C8-H), 7.33 (d, *J* = 8.8 Hz, 2H, C1, C3-H). ^{13}C NMR (75 MHz, CDCl$_3$) δ 149.92 (s, C2), 141.39 (s, C8), 136.90 (s, C12), 132.87 (s, C9), 131.86 (s, C10, C14), 130.67 (s, C5),

130.52 (s, C11, C13), 129.36 (s, C4, C6), 127.58 (s, C1, C3), 121.42 (s, C2-OCF_3), 117.35 (s, C7-CN), 110.99 (s, C7). HRMS (ESI) m/z calcd for $C_{16}H_{10}ClF_3NO$ ([M+H]$^+$) 324.03975, found 324.03995.

2.4. (Z)-3-(4-bromophenyl)-2-(4-(trifluoromethoxy)phenyl)acrylonitrile (1a2d)

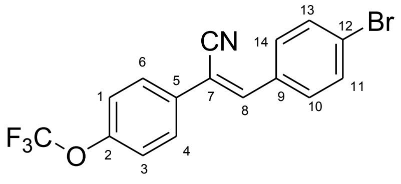

White powder; yield 46%. M.p.75-77°C. ^{1}H NMR (300 MHz, $CDCl_3$) δ 7.76 (s, 2H, C10, C14-H), 7.72 (d, J = 8.8 Hz, 2H, C11, C13-H), 7.65 (s, 2H, C4, C6-H), 7.47 (s, 1H, C8-H), 7.33 (d, J = 8.6 Hz, 2H, C1, C3-H). ^{13}C NMR (75 MHz, $CDCl_3$) δ 149.85 (s, C2), 141.34 (s, C8), 132.75 (s, C9), 132.23 (s, C10, C14), 130.56 (s, C11, C13), 127.48 (s, C5), 125.18 (s, C4, C6), 122.02 (s, C12), 121.32 (s, C1, C3), 118.60 (s, C2-OCF_3), 117.23 (s, C7-CN), 111.02 (s, C7). HRMS (ESI) m/z calcd for $C_{16}H_9BrF_3NO$ ([M]$^-$) 366.98141, found 366.98013.

2.5. (Z)-3-p-tolyl-2-(4-(trifluoromethoxy)phenyl)acrylonitrile (1a2e)

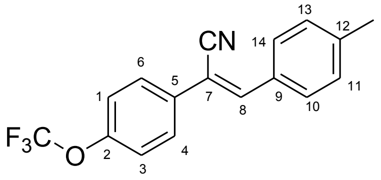

White needle crystal; yield 51%. M.p.78-80°C. ^{1}H NMR (300 MHz, $CDCl_3$) δ 7.82 (d, J = 8.2 Hz, 2H, C10, C14-H), 7.77 – 7.68 (m, 2H, C4, C6-H), 7.50 (s, 1H, C8-H), 7.32 (s, 2H), 7.29 (s, 2H, C1, C3-H), 2.44 (s, 3H, C12-CH_3). ^{13}C NMR (75 MHz, $CDCl_3$) δ 149.61 (s, C2), 143.04 (s, C8), 141.58 (s, C12), 133.42 (s, C9), 130.75 (s, C10, C14), 129.76 (s, C5), 129.40 (s, C11, C13), 127.43 (s, C4, C6), 122.17 (s, C2-OCF_3) 121.35 (s, C1, C3), 117.85 (s, C7-CN), 109.08 (s, C7), 21.52 (s, C12-CH_3). HRMS (ESI) m/z calcd for $C_{17}H_{12}F_3NO$ ([M−H]$^-$) 302.07982, found 302.07944.

2.6. (Z)-3-(4-ethylphenyl)-2-(4-(trifluoromethoxy)phenyl)acrylonitrile (1a2f)

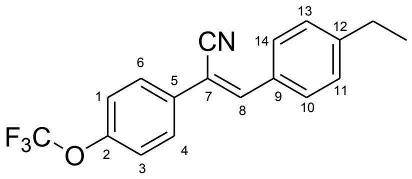

White needle crystal; yield 61%. M.p.82-85°C. ^{1}H NMR (300 MHz, $CDCl_3$) δ 7.85 (d, J = 8.2 Hz, 2H, C4, C6-H), 7.76 – 7.67 (m, 2H, C10, C14-H), 7.51 (s, 1H, C8-H), 7.35 (s, 2H, C11, C13-H), 7.30 (s, 2H, C1, C3-H), 2.74 (q, J = 7.6 Hz, 2H, C12-CH_2CH_3), 1.30 (t, J = 7.6 Hz, 3H, C12-CH_2CH_3). ^{13}C NMR (75 MHz, $CDCl_3$) δ 149.57 (s, C12), 147.85 (s, C2), 143.11 (s, C8), 133.39 (s, C9), 130.91 (s, C5), 129.50 (s, C10, C14), 128.59 (s, C11, C13), 127.44 (s, C4, C6), 121.75 (s, C1, C3), 121.40 (s, C2-OCF_3), 117.92 (s, C7-CN), 109.05 (s, C7), 28.90 (s, C12-$CH(CH_3)_2$), 15.23 (s, C12-$CH(CH_3)_2$). HRMS (ESI) m/z calcd for $C_{18}H_{14}F_3NO$ ([M+Na]$^+$) 340.09197, found 340.09198.

2.7. (Z)-3-(4-isopropylphenyl)-2-(4-(trifluoromethoxy)phenyl)acrylonitrile (1a2g)

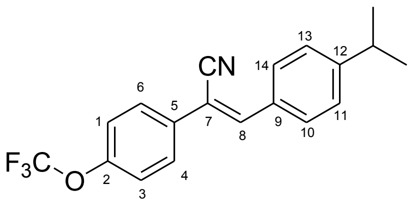

White needle crystal; yield 61%. M.p.82-85°C. ^{1}H NMR (300 MHz, $CDCl_3$) δ 7.85 (d, J = 8.2 Hz, 2H, C10, C14-H), 7.71 (d, J = 8.9 Hz, 2H, C4, C6-H), 7.51 (s, 1H, C8-H), 7.36 (d, J = 8.3 Hz, 2H, C11, C13-H), 7.31 (d, J = 8.2 Hz, 2H, C1, C3-H), 2.99 (dt, J = 13.7, 6.8 Hz, 1H, C12-$CH(CH_3)_2$), 1.30 (d, J = 6.9 Hz, 6H, C12-$CH(CH_3)_2$). ^{13}C NMR (75 MHz, $CDCl_3$) δ 152.42 (s, C12), 149.62 (s, C2), 143.05 (s, C8), 133.45 (s, C9), 131.09 (s, C5), 129.54 (s, C10, C14), 127.45 (s, C11, C13), 127.17 (s, C4, C6), 122.16 (s, C1, C3), 121.37 (s, C2-OCF_3), 117.87 (s, C7-CN), 109.14 (s, C7), 34.20 (s, C12-$CH(CH_3)_2$), 23.66 (s, C12-$CH(CH_3)_2$). HRMS (ESI) m/z calcd for $C_{19}H_{17}F_3NO$ ([M+H]$^+$) 332.12568, found 332.12543.

2.8. (Z)-3-(4-(dimethylamino)phenyl)-2-(4-(trifluoromethoxy)phenyl)acrylonitrile (1a2h)

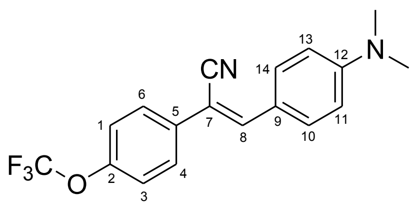

Yellow needle crystal; yield 70%. M.p.88-90°C. ^{1}H NMR (500 MHz, $CDCl_3$) δ 7.86 (d, *J* = 8.8 Hz, 2H, C10, C14-H), 7.64 (d, *J* = 8.8 Hz, 2H, C4, C6-H), 7.37 (s, 1H, C8-H), 7.23 (d, *J* = 14.0 Hz, 2H, C11, C13-H), 6.74 (d, *J* = 8.8 Hz, 2H, C1, C3-H), 3.07 (s, 6H, C12-N(CH_3)$_2$). ^{13}C NMR (75 MHz, $CDCl_3$) δ 151.96 (s, C12), 145.30 (s, C2), 143.25 (s, C8), 134.45 (s, C10, C14), 131.79 (s, C5), 131.44 (s, C4, C6), 130.67 (s, C1, C3), 126.84 (s, C9), 121.34 (s, C2-OCF_3), 121.28 (s, C7-CN), 111.67 (s, C11, C13), 111.34 (s, C7), 39.94 (d, *J* = 6.2 Hz, C12-N(CH_3)$_2$). HRMS (ESI) m/z calcd for $C_{18}H_{16}F_3N_2O$ ([M+H]$^+$) 333.12092, found 333.12045.

2.9. (Z)-3-(4-(diethylamino)phenyl)-2-(4-(trifluoromethoxy)phenyl)acrylonitrile (1a2i)

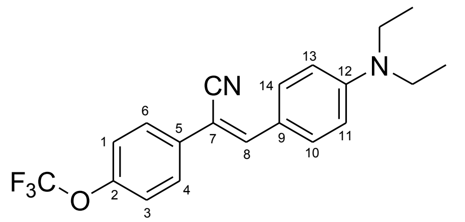

Yellow needle crystal; yield 73%. M.p.80-82°C. ^{1}H NMR (300 MHz, $CDCl_3$) δ 7.86 (d, *J* = 9.0 Hz, 2H, C10, C14-H), 7.66 (d, *J* = 8.8 Hz, 2H, C4, C6-H), 7.37 (s, 1H, C8-H), 7.27 (d, *J* = 8.8 Hz, 2H, C11, C13-H), 6.71 (d, *J* = 9.0 Hz, 2H, C1, C3-H), 3.50–3.40 (m, 4H, C12-N(CH_2)$_2$(CH_3)$_2$), 1.24 (t, *J*= 7.1 Hz, 6H, C12-N(CH_2)$_2$(CH_3)$_2$). ^{13}C NMR (75 MHz, $CDCl_3$) δ 148.76 (s, C12), 143.04 (s, C2), 134.40 (s, C8), 132.14 (s, C5), 131.74 (s, C10, C14), 130.68 (s, C4, C6), 126.81 (s, C1, C3), 122.15 (s, C9), 121.34 (d, *J* = 4.3 Hz, C11, C13), 119.20 (s, C2-OCF_3), 118.74 (s, C7-CN), 111.55 (d, *J* =10.4 Hz, C7), 47.10–41.32 (m, C12-N(CH_2CH_3)$_2$), 12.45 (s, C12-N(CH_2CH_3)$_2$). HRMS (ESI) m/z calcd for $C_{20}H_{19}F_3N_2O$ ([M−H]$^-$) 359.13767, found 359.13678.

2.10. (Z)-3-phenyl-2-(3-(trifluoromethoxy)phenyl)acrylonitrile (1b2a)

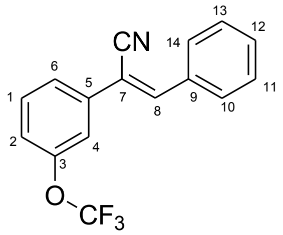

White powder; yield 54%. M.p.64-66°C. ^{1}H NMR (300 MHz, $CDCl_3$) δ 7.95 – 7.92 (m, 2H, C10, C14-H), 7.66 (d, *J* = 7.4 Hz, 1H, C8-H), 7.58 (s, 1H, C1-H), 7.53 (d, *J* = 5.7 Hz, 2H, C11, C13-H), 7.50 (dd, *J* = 5.0, 3.1 Hz, 2H, C6, C12-H), 7.30 (s, 2H, C2, C4-H). ^{13}C NMR (75 MHz, $CDCl_3$) δ 149.82 (s, C3), 143.63 (s, C8), 136.61 (s, C5), 133.23 (s, C12), 131.08 (s, C9), 130.54 (s, C1), 129.46 (s, C10, C14), 129.08 (s, C11, C13) 124.47 (s, C2), 121.43 (s, C3-C6), 118.61 (s, OCF_3), 117.50 (s, C7-CN), 115.81 (s, C4)110.29 (s, C7). HRMS (ESI) m/z calcd for $C_{16}H_{10}F_3NO$ ([M]$^-$) 289.07200, found 289.07104.

2.11. (Z)-3-(4-fluorophenyl)-2-(3-(trifluoromethoxy)phenyl)acrylonitrile (1b2b)

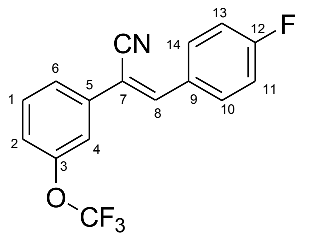

Yellow powder; yield 73%. M.p.80-82°C. ^{1}H NMR (300 MHz, $CDCl_3$) δ 7.95 (s, 2H, C14-H), 7.92 (s, 1H, C10-H), 7.62 (d, *J* = 7.7 Hz, 1H, C8-H), 7.55 – 7.45 (m, 3H, C1, C11, C13-H) 7.25 (d, *J* = 8.3 Hz, 1H, C6-H), 7.03 (s, 1H, C4-H), 7.00 (s, 1H,

C2-H). ^{13}C NMR (75 MHz, $CDCl_3$) δ 161.91 (s, C12), 149.86 (s, C3), 143.14 (s, C8), 137.10 (s, C5), 131.47 (s, C10, C14), 130.41 (s, C1), 126.07 (s, C9), 124.20 (s, C6, C2), 120.86 (m, C3-OCF_3), 118.39 (s, C7-CN), 118.16 (s, C4), 114.54 (s, C11, C13), 107.22 (s, C7). HRMS (ESI) m/z calcd for $C_{16}H_9F_4NO$ ([M−H]$^-$) 306.05475, found 306.05429.

2.12. (Z)-3-(4-chlorophenyl)-2-(3-(trifluoromethoxy)phenyl)acrylonitrile (1b2c)

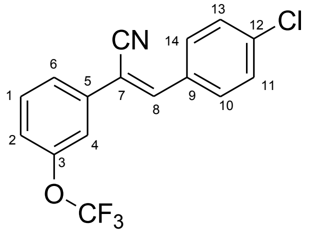

White powder; yield 43%. M.p.86-88□. ^{1}H NMR (300 MHz, $CDCl_3$) δ 7.88 (s, 1H, C10-H), 7.86 (s, 1H, C14-H), 7.64 (d, J = 8.6 Hz, 1H, C8-H), 7.53 (d, J = 6.9 Hz, 3H, C11, C13, C1-H), 7.51 – 7.46 (m, 2H, C4, C6-H), 7.31 (s, 1H, C2-H). ^{13}C NMR (75 MHz, $CDCl_3$) δ 149.84 (s, C12), 142.05 (s, C3), 137.08 (s, C8), 136.29 (s, C5), 131.65 (s, C1), 130.66 (s, C10, C14), 130.62 (s, C9), 129.41 (s, C6), 124.49 (m, C2), 122.13 (s, C3-OCF_3)121.66 (s, C7-CN), 118.60 (s, C4), 117.26 (s, C11, C13), 110.86 (s, C7). HRMS (ESI) m/z calcd for $C_{16}H_9ClF_3NO$ ([M+H]$^+$) 324.03975, found 324.03973.

2.13. (Z)-3-(4-bromophenyl)-2-(3-(trifluoromethoxy)phenyl)acrylonitrile (1b2d)

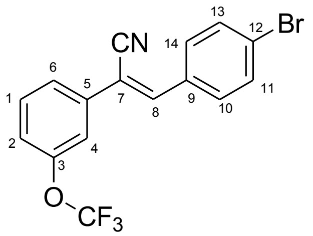

Tan powder; yield 43%. M.p.86-88°C. ^{1}H NMR (300 MHz, $CDCl_3$) δ 7.78 (d, J = 8.7 Hz, 2H, C10, C14-H), 7.64 (d, J = 8.6 Hz, 2H, C11, C13-H), 7.60 (s, 1H, C8-H), 7.51 – 7.45 (m, 1H, C1-H), 7.43 (d, J = 1.4 Hz, 1H, C6-H), 7.38 (s, 1H, C4-H), 7.35 (s, 1H, C2-H). ^{13}C NMR (75 MHz, $CDCl_3$) δ 149.89 (s, C3), 142.05 (s, C8), 136.32 (s, C5), 132.36 (s, C9), 132.12 (s, C10, C14), 130.78(s, C11, C13), 130.61 (s, C1), 125.50 (s, C6), 124.46 (s, C2), 122.17 (s, C12), 121.61 (s, C3-OCF_3), 118.56 (s, C7-CN), 117.18 (s, C4), 111.05 (s, C7). HRMS (ESI) m/z calcd for $C_{16}H_{10}BrF_3NO$ ([M+H]$^+$) 369.98924, found 369.38141.

2.14. (Z)-3-p-tolyl-2-(3-(trifluoromethoxy)phenyl)acrylonitrile (1b2e)

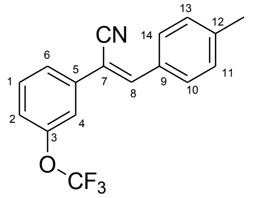

White needle crystal; yield 47%. M.p.78-80°C. ^{1}H NMR (300 MHz, $CDCl_3$) δ 7.82 (d, J = 7.6 Hz, 2H, C10, C14-H), 7.57 (d, J = 7.1 Hz, 1H, C8-H), 7.46 – 7.41 (m, 1H, C1-H), 7.39 (s, 1H, C6-H), 7.37 (s, 1H, C4-H), 7.34 – 7.27 (m, 2H, C11, C13-H), 2.45 (d, J = 6.5 Hz, 3H, C12-CH_3). ^{13}C NMR (75 MHz, $CDCl_3$) δ 149.79 (s, C3), 143.53 (s, C8), 141.74 (s, C5), 136.83 (s, C12), 130.54 (s, C9), 130.39 (s, C10, C14), 129.73 (s, C1), 129.48 (s, C11, C13) 124.27 (s, C2), 122.15 (s, C6), 121.05 (s, C3-OCF_3), 118.41 (s, C7-CN), 117.62 (s, C4), 108.96 (s, C7), 21.48 (s, C12-CH_3). HRMS (ESI) m/z calcd for $C_{17}H_{12}F_3NO$ ([M+NH_4]$^+$) 321.12092, found 321.12030.

2.15. (Z)-3-(4-ethylphenyl)-2-(3-(trifluoromethoxy)phenyl)acrylonitrile (1b2f)

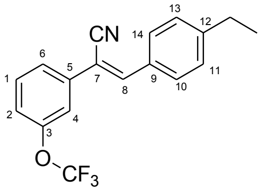

White powder; yield 39%. M.p. > 240°C. ^{1}H NMR (300 MHz, $CDCl_3$) δ 7.86 (d, *J* = 8.3 Hz, 2H), 7.64 (d, *J* = 7.9 Hz, 1H), 7.54 (d, *J* = 6.8 Hz, 2H), 7.49 (d, *J* = 7.9 Hz, 1H), 7.34 (d, *J* = 8.2 Hz, 2H), 7.26 (s, 1H), 2.74 (dd, *J* = 15.2, 7.6 Hz, 2H), 1.28 (d, *J* = 7.5 Hz, 3H). ^{13}C NMR (75 MHz, $CDCl_3$) δ 149.84 (s, C3), 148.04 (s, C12), 143.62 (s, C8), 136.91 (s, C5), 130.81 (s, C9), 130.45 (s, C1), 129.63 (s, C10, C14), 128.60 (s, C11, C13), 124.36 (s, C2), 122.19 (s, C6), 121.13 (s, C3-OCF_3), 118.49 (s, C7-CN), 117.69(s, C4)109.09 (s, C7), 29.70 (s, C12-CH_2CH_3), 15.12 (s, C12-CH_2CH_3). HRMS (ESI) m/z calcd for $C_{18}H_{14}F_3NO$ ([M+NH_4]$^+$) 335.13657, found 335.13785.

2.16. (Z)-3-(4-isopropylphenyl)-2-(3-(trifluoromethoxy)phenyl)acrylonitrile (1b2g)

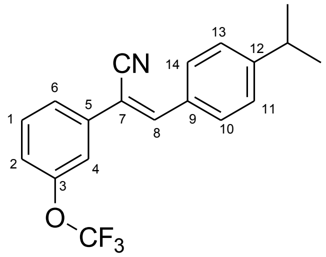

White powder; yield 20%. M.p. > 240°C. ^{1}H NMR (300 MHz, $CDCl_3$) δ 7.87 (d, *J* = 8.3 Hz, 2H, C10, C14-H), 7.64 (d, *J* = 8.0 Hz, 1H, C8-H), 7.55 (s, 1H, C1-H), 7.51 (d, *J* = 8.1 Hz, 2H, C11, C13-H), 7.37 (d, *J* = 8.2 Hz, 2H, C6, C4-H), 7.26 (s, 1H, C2-H), 3.00 (dd, *J* = 13.8, 7.1 Hz, 1H, C12-$CH(CH_3)_2$), 1.28 (d, *J* = 6.5 Hz, 7H, C12-$CH(CH_3)_2$). ^{13}C NMR (75 MHz, $CDCl_3$) δ 152.62 (s, C3), 149.87 (s, C12), 143.60 (s, C8), 136.91 (s, C5), 130.93 (s, C9), 130.45 (s, C1), 129.67 (s, C10, C14), 127.19 (s, C6), 124.36 (s, C11, C13), 122.19 (s, C2), 121.13 (s, C3-OCF_3), 118.49 (s, C7-CN), 117.70 (s, C4), 109.11 (s, C7), 34.21 (s, C12-$CH(CH_3)_2$), 23.66 (s, C12-$CH(CH_3)_2$). HRMS (ESI) m/z calcd for $C_{19}H_{16}F_3NO$ ([M+H]$^+$) 330.11112, found 330.10953.

2.17. (Z)-3-(4-(dimethylamino)phenyl)-2-(3-(trifluoromethoxy)phenyl)acrylonitrile (1b2h)

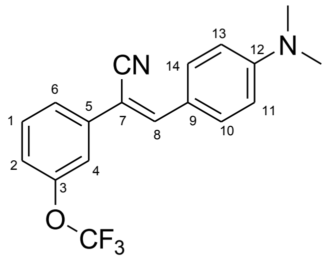

Yellow powder; yield 41%. M.p.89-90°C. ^{1}H NMR (300 MHz, $CDCl_3$) δ 7.89 (d, *J* = 9.0 Hz, 2H, C10, C14-H), 7.59 (d, *J* = 8.0 Hz, 1H, C8-H), 7.48 (s, 1H, C1-H), 7.43 (s, 1H, C6-H), 7.19 (d, *J* = 7.2 Hz, 1H, C4-H), 7.11 (d, *J* = 8.5 Hz, 1H, C2-H), 6.75 (d, *J* = 9.0 Hz, 2H, C11, C13-H), 3.10 (s, 6H, C12-$N(CH_3)_2$). ^{13}C NMR (75 MHz, $CDCl_3$) δ 152.08 (s, C3), 149.83 (s, C12), 143.68 (s, C8), 137.91 (s, C5), 131.64 (s, C10, C14), 130.47 (s, C1), 126.32 (s, C6), 123.80 (s, C2), 121.24 (d, *J* = 15.6 Hz, C9), 120.84 (s, C3-OCF_3), 119.96 (s, C7-CN), 117.91 (s, C4), 111.88 (d, *J* = 36.6 Hz, C11, C13), 111.64 – 111.44 (m, C7), 40.02 (d, *J* = 12.2 Hz, C12-$N(CH_3)_2$). HRMS (ESI) m/z calcd for $C_{18}H_{15}F_3N_2O$ ([M+H]$^+$) 332.11420, found 332.11258.

2.18. (Z)-3-(4-morpholinophenyl)-2-(3-(trifluoromethoxy)phenyl)acrylonitrile (1b2i)

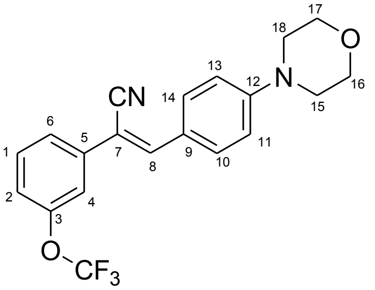

Yellow powder; yield 27%. M.p.78-80°C. ^{1}H NMR (300 MHz, $CDCl_3$) δ 7.91 (d, *J* = 8.4 Hz, 2H, C10, C14-H), 7.61 (d, *J* = 7.8 Hz, 1H, C8-H), 7.48 (d, *J* = 7.6 Hz, 2H, C11, C13-H), 7.45 (s, 1H, C1-H), 7.22 (d, *J* = 8.2 Hz, 1H, C6-H), 6.97 (s, 1H, C4-H), 6.94 (s, 1H, C2-H), 3.90 (d, *J* = 4.2 Hz, 4H, C16, C17-H), 3.35 (d, *J* = 4.2 Hz, 4H, C15, C18-H). ^{13}C NMR (75 MHz, $CDCl_3$) δ 152.63 (s, C3), 149.75 (s, C12), 143.07 (s, C8), 137.33 (s, C5), 131.28 (s, C10, C14), 130.22 (s, C1), 123.94 (s, C9), 122.09 (s, C6), 120.39 (s, C2), 118.32 (s, C3-OCF_3), 118.02 (s, C7-CN), 114.11 (s, C4), 111.55 (s, C11, C13), 105.23 (s, C7), 66.47 (s, C16, C17), 47.57 (s, C15, C18). HRMS (ESI) m/z calcd for $C_{20}H_{18}F_3N_2O_2$ ([M+H]$^+$) 375.13149, found 375.13120.

2.19. (Z)-3-(pyridin-2-yl)-2-(3-(trifluoromethoxy)phenyl)acrylonitrile (1b3a)

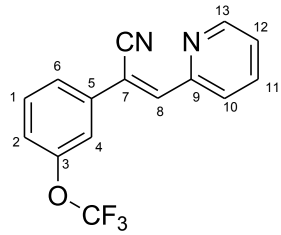

Brown powder; yield 30%. M.p.70-72°C. ^{1}H NMR (300 MHz, $CDCl_3$) δ 8.80 (d, *J* = 4.3 Hz, 1H, C13-H), 7.98 (d, *J* = 7.9 Hz, 1H, C10-H), 7.87 (dd, *J* = 7.7, 1.7 Hz, 1H, C12-H), 7.72 (d, *J* = 8.7 Hz, 1H, C11-H), 7.70 (s, 1H, C8-H), 7.61 (s, 1H, C1-H), 7.53 (t, *J* = 8.0 Hz, 1H, C6-H), 7.39 (dd, *J* = 7.4, 4.8 Hz, 1H, C5-H), 7.32 (d, *J* = 8.2 Hz, 1H, C2-H). ^{13}C NMR (75 MHz, $CDCl_3$) δ 151.73 (s, C3), 150.12 (s, C9), 149.85 (s, C13), 142.33 (s, C8), 136.84 (s, C5), 136.21 (s, C11), 130.58 (s, C1), 126.37 (s, C6), 124.72 (d, *J* = 14.0 Hz, C2), 124.35 (s, C10, C12), 121.95 (s, C7-CN), 118.93 (s, C3-OCF_3), 116.86 (s, C4), 113.58 (s, C7). HRMS (ESI) m/z calcd for $C_{15}H_9F_3N_2O$ ($[M+H]^+$) 291.07397, found 291.07309.

2.20. (Z)-3-phenyl-2-(2-(trifluoromethoxy)phenyl)acrylonitrile (1c2a)

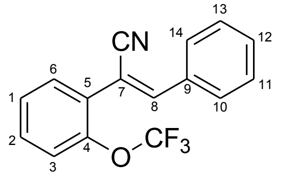

Brown oily liquid; yield: 50.61 %. ^{1}H NMR (300 MHz, $CDCl_3$) δ 7.91 (dd, *J* = 6.5, 2.9 Hz, 2H, C10, C14-H), 7.60 – 7.56 (m, 1H, C8-H), 7.51 (dd, *J* = 4.9, 1.9 Hz, 3H, C6, C11, C13-H), 7.49 – 7.45 (m, 1H, C12-H), 7.43 (m, 1H, C2-H) 7.39 (dd, *J* = 9.0, 2.4 Hz, 2H, C1, C3-H). ^{13}C NMR (75 MHz, $CDCl_3$) δ 148.10 (s, C4), 146.43 (s, C8), 133.35 (s, C5), 131.04 (s, C12), 130.62 (s, C9), 130.57 (s, C6), 129.35 (s, C10, C14), 129.02 (s, C11, C13), 127.37 (s, C2), 125.30 (s, C1), 121.36 (s, C3), 118.73 (s, C4-OCF_3), 117.34 (s, C7-CN), 106.69 (s, C7). HRMS (ESI) m/z calcd for $C_{16}H_{10}F_3NO$ ($[M-H]^-$) 288.06417, found 288.06369 .

2.21. (Z)-3-(4-chlorophenyl)-2-(2-(trifluoromethoxy)phenyl)acrylonitrile (1c2b)

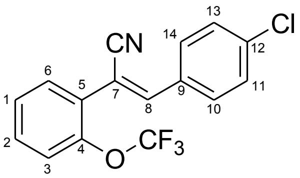

White powder; yield 16%. M.p.58-60°C. ^{1}H NMR (300 MHz, $CDCl_3$) δ 7.85 (d, *J* = 8.5 Hz, 2H, C10, C14-H), 7.60 – 7.55 (m, 1H, C8-H), 7.50 (d, *J* = 5.5 Hz, 2H, C11, C13-H), 7.47 (s, 1H, C6-H), 7.43 (s, 1H, C2-H), 7.39 (d, *J* = 12.1 Hz, 2H, C1, C3-H). ^{13}C NMR (75 MHz, $CDCl_3$) δ 147.85 (s, C4), 146.37 (s, C8), 136.96 (s, C5), 131.70 (s, C12), 130.61 (s, C9), 130.44(s, C10, C14) 129.22 (s, C11, C13), 128.56 (s, C6), 127.27 (s, C2), 122.06 (s,) C1, 121.27 (s, C3), 118.63(s, C4-OCF_3), 116.93 (s, C7-CN), 107.27 (s, C7). HRMS (ESI) m/z calcd for $C_{16}H_9ClF_3NO$ ($[M-H]^-$) 322.02520, found 322.02417.

2.22. (Z)-3-(4-bromophenyl)-2-(2-(trifluoromethoxy)phenyl)acrylonitrile (1c2c)

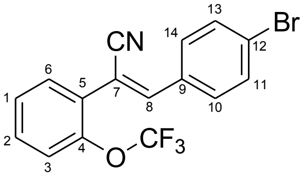

White powder; yield 30%. M.p.60-62°C. ^{1}H NMR (300 MHz, $CDCl_3$) δ 7.79 (d, *J* = 8.5 Hz, 2H, C10, C14-H), 7.66 (s, 2H, C11, C13-H), 7.63 (s, 1H, C8-H), 7.54 (s, 1H, C6-H), 7.52 (s, 1H, C2-H), 7.50 (d, *J* = 4.0 Hz, 1H, C1-H), 7.31 (s, 1H, C3-H). ^{13}C NMR (75 MHz, $CDCl_3$) δ 146.56 (s, C4), 132.31 (s, C8), 132.23 (s, C5), 130.74 (s, C9), 130,68 (s, C10, C14), 130.54 (s, C11, C13), 128.65 (s, C6), 127.39 (s, C2), 125.46 (s, C1), 122.17 (s, C3), 121.38 (s, C12), 118.73 (s, C4-OCF_3), 117.02 (s, C7-CN), 107.52 (s, C7). HRMS (ESI) m/z calcd for $C_{16}H_9BrF_3NO$ ($[M]^+$) 366.98141, found 366.98050.

2.23. (Z)-3-p-tolyl-2-(2-(trifluoromethoxy)phenyl)acrylonitrile (1c2d)

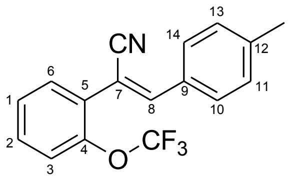

Light yellow oily liquid; yield 30%. M.p.60-62°C. ^{1}H NMR (300 MHz, $CDCl_3$) δ 7.84 (d, *J* = 8.2 Hz, 2H, C10, C14-H), 7.64 (d, *J* = 7.9 Hz, 1H, C8-H), 7.53 (d, *J* = 4.5 Hz, 2H, C11, C13-H), 7.48 (d, *J* = 8.0 Hz, 1H, C6-H), 7.33 (s, 1H, C2-H), 7.30 (s, 1H, C1-H), 7.26 (s, 1H, C3-H), 2.45 (s, 3H, C12-CH_3). ^{13}C NMR (75 MHz, $CDCl_3$) δ 148.05 (s, C4), 146.45 (s, C8), 141.70 (s, C12), 130.73 (s, C5), 130.61 (s, C9), 130.33 (s, C6), 129.71 (s, C10, C14), 129.40 (s, C11, C13), 127.30 (s, C2), 122.20 (s, C1), 121.32 (s, C3), 118.77 (s, C4-OCF_3), 117.53 (s, C7-CN), 105.45 (s, C7), 21.55 (s, C12-CH_3). HRMS (ESI) m/z calcd for $C_{17}H_{13}F_3NO$ ($[M+H]^+$) 304.09438, found 304.09445.

2.24. (Z)-3-(4-methoxyphenyl)-2-(2-(trifluoromethoxy)phenyl)acrylonitrile (1c2e)

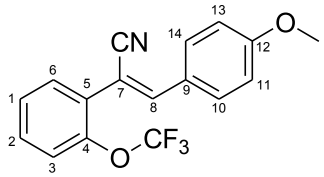

Light yellow oily liquid; yield 30%. M.p.60-62°C. ^{1}H NMR (300 MHz, $CDCl_3$) δ 7.91 (d, *J* = 8.9 Hz, 2H, C10, C14-H), 7.59 – 7.52 (m, 1H, C8-H), 7.44 – 7.37 (m, 2H, C11, C13-H), 7.34 (dd, *J* = 12.2, 3.8 Hz, 2H, C6, C2-H), 7.02 (s, 1H, C1-H), 6.99 (s, 1H, C3-H), 3.90 (s, 3H, C12-OCH_3). ^{13}C NMR (75 MHz, $CDCl_3$) δ 161.88 (s, C12), 147.54 (s, C4), 131.34 (s, C8), 130.57 (s, C10, C14),130.15 (s, C5), 130.06 (s, C6), 129.40 (s, C2), 127.28 (s, C1), 126.22 (s, C9), 121.29 (s, C3), 118.78 (s, C4-OCF_3), 117.85 (s, C7-CN), 114.46 (s, C11, C13), 103.58 (s, C7), 55.37 (d, *J* = 12.3 Hz, C12-OCH_3). HRMS (ESI) m/z calcd for $C_{17}H_{13}F_3NO_2$ ($[M+H]^+$) 320.08929, found 320.08932.

2.25. (Z)-3-(4-(dimethylamino)phenyl)-2-p-tolylacrylonitrile (1d2a)

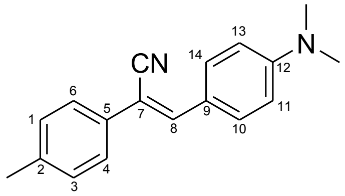

Yellow powder; yield 53%. M.p.68-70°C. ^{1}H NMR (300 MHz, $CDCl_3$) δ 7.86 (d, *J* = 8.5 Hz, 2H, C10, C14-H), 7.54 (d, *J* = 7.5 Hz, 2H, C4, C6-H), 7.38 (s, 1H, C8-H), 7.30 – 7.18 (m, 2H, C11, C13-H), 6.73 (d, *J* = 8.3 Hz, 2H, C1, C3-H), 3.07 (s, 6H, C12-N$(CH_3)_2$), 2.39 (s, 3H, C2-CH_3). ^{13}C NMR (75 MHz, $CDCl_3$) δ 151.62 (s, C12), 141.63 (s, C8), 138.00 (s, C2), 132.82 (s, C5), 131.14 (s, C10, C14), 129.58 (s, C1, C3), 125.68 – 125.38 (s, C4, C6), 121.88 (s, C9), 119.48 (s, C7-CN), 111.70 (s, C11, C13), 104.81 (s, C7), 40.03 (s, C12-N$(CH_3)_2$), 21.12 (s, C2-CH_3). HRMS (ESI) m/z calcd for $C_{18}H_{18}N_2$ ($[M]^+$) 262.14700, found 262.14679.

2.26. (Z)-3-(4-(diethylamino)phenyl)-2-p-tolylacrylonitrile (1d2b)

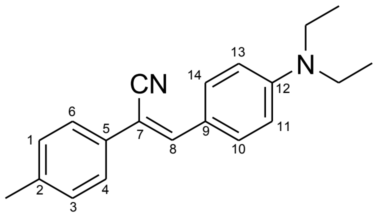

Yellow powder; yield 81%. M.p.142-144°C. ^{1}H NMR (300 MHz, $CDCl_3$) δ 7.85 (d, *J* = 8.6 Hz, 2H, C10, C14-H), 7.53 (d, *J* = 8.2 Hz, 2H, C4, C6-H), 7.37 (s, 1H, C8-H), 7.29 – 7.18 (m, 2H, C11, C13-H), 6.73 (s, 2H, C1, C3-H), 3.44 (d, *J* = 7.0 Hz, 4H, C12-N$(CH_2CH_3)_2$), 2.39 (s, 3H, C2-CH_3), 1.23 (t, *J* = 7.0 Hz, 6H, C12-N$(CH_2CH_3)_2$). ^{13}C NMR (75 MHz, $CDCl_3$) δ 141.61 (s, C12), 137.85 (s, C8), 132.94 (s, C2), 132.05 (s, C10, C14), 131.45 (s, C5), 129.56 (s, C1, C3), 128.86 (s, C4, C6), 125.33 (s, C9), 121.15 (s, C7-CN), 119.62 (s, C11, C13), 111.23 (s, C7), 44.53 (s, C12-N$(CH_2CH_3)_2$), 21.10 (s, C12-N$(CH_2CH_3)_2$), 12.62 (s, C2-CH_3). HRMS (ESI) m/z calcd for $C_{20}H_{22}N_2$ ($[M]^+$) 307.18159, found 307.18057.

2.27. (Z)-3-(3,4,5-trimethoxyphenyl)-2-(4-methoxyphenyl)acrylonitrile (1e2a)

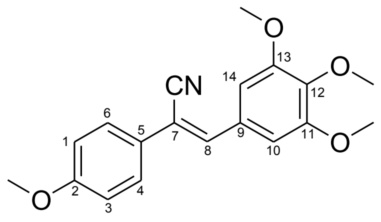

White needle crystal; yield 60%. M.p.110-112°C. ^{1}H NMR (300 MHz, $CDCl_3$) δ 7.62 (d, *J* = 8.7 Hz, 2H, C4, C6-H), 7.36 (s, 1H, C8-H), 7.18 (s, 2H, C10, C14-H), 6.98 (d, *J* = 8.7 Hz, 2H, C1, C3-H), 3.95 (s, 6H, C11, C13-OCH_3), 3.94 (s, 3H, C2-OCH_3), 3.88 (s, 3H, C12-OCH_3). ^{13}C NMR (75 MHz, $CDCl_3$) δ 160.44 (s, C2), 153.32 (s, C11, C13), 140.07 (s, C8, C12), 129.33 (s, C9), 127.24 (s, C4, C6), 127.11 (s, C5), 118.45 (s, C7-CN), 114.50 (s, C1, C3), 110.15 (s, C7), 106.70 (s, C10, C14), 60.96 (s, C12-OCH_3), 56.30 (s, C11, C13-OCH_3), 55.43 (s, C2-OCH_3). HRMS (ESI) m/z calcd for $C_{19}H_{20}NO_4$ ($[M+H]^+$) 326.13868, found 326.13852.

2.28. (Z)-3-(4-(dimethylamino)phenyl)-2-(4-(trifluoromethyl)phenyl)acrylonitrile (1f2a)

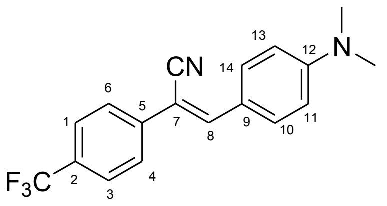

Yellow powder; yield 43%. M.p.128-130°C. ^{1}H NMR (300 MHz, $CDCl_3$) δ 7.90 (d, *J* = 9.0 Hz, 2H, C10, C14-H), 7.75 (d, *J* = 8.5 Hz, 2H, C1, C3-H), 7.67 (d, *J* = 8.5 Hz, 2H, C4, C6-H), 7.49 (s, 1H, C8-H), 6.77 (d, *J* = 9.0 Hz, 2H, C11, C13-H), 3.10 (s, 6H, C12-N$(CH_3)_2$). ^{13}C NMR (75 MHz, $CDCl_3$) δ 152.17 (s, C12), 145.92 (s, C8), 144.21 (s, C5), 131.90 (s, C2),131.78 (s, C10, C14), 129.50 (s, C4, C6), 125.56 (s, C1, C3), 121.12 (s, C2-CF_3), 118.98 (s, C9), 111.65 (s, C7-CN), 111.36 (s, C11, C13), 102.75 (s, C7), 39.96 (s, C12-N$(CH_3)_2$). HRMS (ESI) m/z calcd for $C_{18}H_{16}F_3N_2$ ($[M+H]^+$) 317.12601, found 317.12610.

2.29. (Z)-3-(4-(dimethylamino)phenyl)-2-(3,4,5-trimethoxyphenyl)acrylonitrile (1g2a)

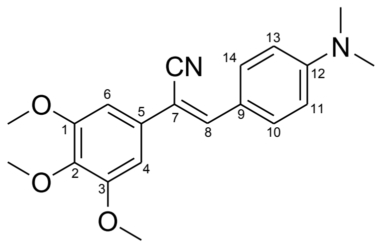

Yellow powder; yield 51%. M.p.58-60°C. ^{1}H NMR (300 MHz, $CDCl_3$) δ 7.85 (d, *J* = 9.0 Hz, 2H, C10, C14-H), 7.33 (s, 1H, C8-H), 6.83 (s, 2H, C11, C13-H), 6.73 (d, *J* = 9.0 Hz, 2H, C4, C6-H), 3.93 (s, 6H, C1, C3-H), 3.88 (s, 3H, C2-H), 3.07 (s, 6H, C12-N$(CH_3)_2$). ^{13}C NMR (75 MHz, $CDCl_3$) δ 153.55 (s, C1, C3), 151.72 (s, C12), 142.11 (s, C8), 138.47 (s, C2), 131.37 (s, C10, C14), 131.21 (s, C5), 121.56 (s, C9), 119.43 (s, C7-CN), 111.67 (s, C11, C13), 104.61 (s, C7), 103.17 (s, C4, C6), 60.94 (s, C2-OCH_3), 56.36 (s, C1, C3-OCH_3), 40.00 (s, C12-N$(CH_3)_2$). HRMS (ESI) m/z calcd for $C_{20}H_{23}N_2O_3$ ($[M+H]^+$) 339.17032, found 339.17010.

2.30. (Z)-3-(4-(diethylamino)phenyl)-2-(3,4,5-trimethoxyphenyl)acrylonitrile (1g2b)

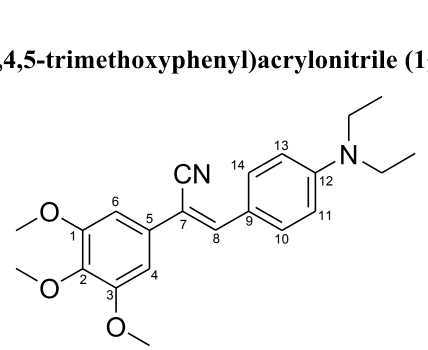

Yellow powder; yield 48%. M.p.68-70°C. ^{1}H NMR (300 MHz, $CDCl_3$) δ 7.83 (d, *J* = 9.0 Hz, 2H, C10, C14-H), 7.30 (s, 1H, C8-H), 6.82 (s, 2H, C11, C13-H), 6.69 (d, *J* = 9.0 Hz, 2H, C4, C6-H), 3.93 (s, 6H, C1, C3-OCH_3), 3.87 (s, 3H, C2-OCH_3), 3.43

(q, *J* = 7.1 Hz, 4H, C12-N($CH_2CH_3)_2$), 1.22 (t, *J* = 7.1 Hz, 6H, C12-N($CH_2CH_3)_2$).^{13}C NMR (75 MHz, $CDCl_3$) δ 153.73 (s, C12), 153.49 (s, C1, C3), 141.93 (s, C8), 138.20 (s, C2), 132.26(s, C5), 131.52 (s, C10, C14), 121.90 (s, C9), 121.22 (s, C7-CN), 111.71 (s, C11, C13), 105.93 (s, C7), 102.87 (s, C4, C6), 61.00 (s, C2-OCH_3), 56.30 (s, C1-OCH_3), 56.19 (s, C3-OCH_3), 45.75–44.66 (m, C12-N($CH_2CH_3)_2$), 12.49 (s, C12-N($CH_2CH_3)_2$). HRMS (ESI) m/z calcd for $C_{22}H_{27}N_2O_3$ ($[M+H]^+$) 367.20162, found 367.20102.

2.31.(2Z)-3-(4-((E)-3-(4-methoxyphenyl)-3-oxoprop-1-enyl)phenyl)-2-(4-(trifluoromethoxy)phenyl)acrylonitrile (8a)

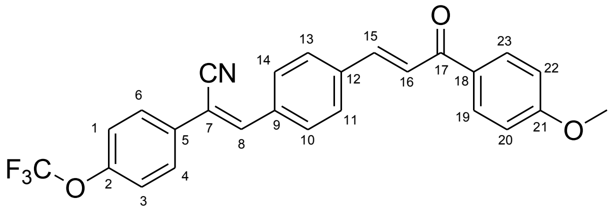

Light yellow powder; yield 46%. M.p.148-150°C. ^{1}H NMR (300 MHz, $CDCl_3$) *δ* 8.08 (d, *J* = 8.6 Hz, 2H, C19, C23-H), 7.97 (d, *J* = 8.3 Hz, 2H, C4, C6-H), 7.83 (d, *J* = 14.2 Hz, 1H, C8-H), 7.81 – 7.68 (m, 4H, C10, C14, C11, C13-H), 7.67 (s, 1H, C15-H), 7.54 (s, 1H, C16-H), 7.34 (d, *J* = 8.5 Hz, 2H, C20, C22-H), 7.02 (d, *J* = 8.5 Hz, 2H, C1, C3-H), 3.93 (s, 3H, C21-OCH_3). ^{13}C NMR (75 MHz, DMSO-d6) *δ* 187.21 (s, C17), 163.32 (s, C21), 148.79 (s, C15), 143.07 (s, C2), 141.68 (s, C8), 137.05 (s, C12), 135.00 (s, C5), 132.97 (s, C9), 130.92 (s, C19, C23), 130.34 (s, C18), 129.59 (s, C10, C14), 129.13 (s, C11, C13), 127.93 (s, C4, C6), 123.68 (s, C1, C3), 123.00 (s, C2-OCF_3), 121.55 (s, C16), 117.54 (s, C7-CN)114.02 (s, C20, C22), 109.49 (s, C7), 55.54 (s, C21-OCH_3). HRMS (ESI) m/z calcd for $C_{26}H_{18}F_3NO_3$ ($[M+H]^+$) 449.42100, found 450.19421.

2.32. (Z)-3-(4-methoxyphenyl)-2-(4-(trifluoromethoxy)phenyl)acrylonitrile (1a2j)

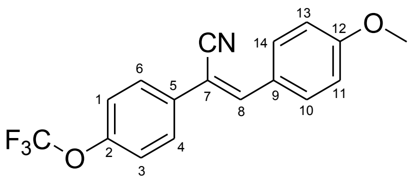

White flaky crystal; yield 67%. M.p.68-70°C. ^{1}H NMR (300 MHz, $CDCl_3$) δ 7.91 (d, *J* = 8.9 Hz, 2H, C10, C14-H), 7.75 – 7.66 (m, 2H, C4, C6-H), 7.46 (s, 1H, C8-H), 7.35 – 7.29 (m, 2H, C11, C13-H), 7.01 (d, *J* = 8.9 Hz, 2H, C1, C3-H), 3.90 (s, 3H, C12-OCH_3). ^{13}C NMR (75 MHz, $CDCl_3$) δ 161.77 (s, C12), 149.40 (s, C2), 142.62 (s, C8), 133.63 (s, C10, C14), 131.31 (s, C5), 127.26 (s, C9), 126.22 (s, C4, C6), 122.16 (s, C2-OCF_3),121.35 (s, C1, C3), 118.16 (s, C1, C3), 114.51 (s, C7-CN), 107.27 (s, C7), 55.44 (s, C12-OCH_3). HRMS (ESI) m/z calcd for $C_{17}H_{13}F_3NO_2$ ($[M+H]^+$) 320.08929, found 320.08936.

2.33. (Z)-3-(3-methoxyphenyl)-2-(4-(trifluoromethoxy)phenyl)acrylonitrile (1a2k)

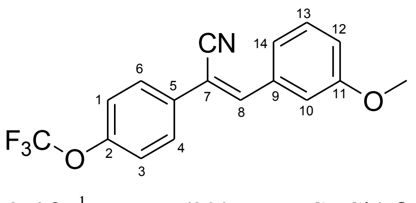

White needle crystal; yield 61%. M.p.82-85°C. ^{1}H NMR (300 MHz, $CDCl_3$) δ 7.77 – 7.70 (m, 2H, C4, C6-H), 7.52 (d, *J* = 2.1 Hz, 1H, C8-H), 7.51 (s, 1H, C13-H), 7.42 (t, *J* = 4.7 Hz, 2H, C10, C14-H), 7.35 – 7.30 (m, 2H, C1, C3-H), 7.04 (d, *J* = 7.5 Hz, 1H, C12-H), 3.90 (s, 3H, C11-OCH_3).

2.34. (Z)-3-(3,4,5-trimethoxyphenyl)-2-(4-(trifluoromethoxy)phenyl)acrylonitrile (1a2l)

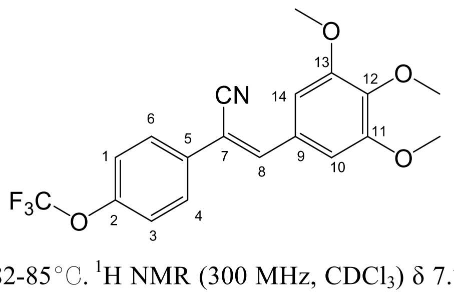

White needle crystal; yield 61%. M.p.82-85°C. ^{1}H NMR (300 MHz, $CDCl_3$) δ 7.76 – 7.68 (m, 2H, C4, C6-H), 7.45 (s, 1H, C8-H), 7.32 (d, *J* = 8.8 Hz, 2H, C11, C13-H), 7.22 (s, 2H, C1, C3-H), 3.95 (s, 6H, C11, C13-OCH_3), 3.95 (s, 3H, C11-OCH_3).

2.35. (Z)-3-(4-propoxyphenyl)-2-(4-(trifluoromethoxy)phenyl)acrylonitrile (1a2m)

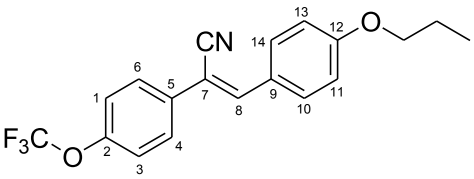

White powder; yield 46%. M.p.88-90°C. ^{1}H NMR (500 MHz, $CDCl_3$) δ 7.88 (d, *J* = 8.7 Hz, 2H, C10, C14-H), 7.67 (d, *J* = 8.7 Hz, 2H, C4, C6-H), 7.43 (s, 1H, C8-H), 7.26 (d, *J* = 5.2 Hz, 2H, C11, C13-H), 6.98 (d, *J* = 8.7 Hz, 2H, C1, C3-H), 3.99 (t, *J* = 6.5 Hz, 2H, C12-O$\underline{CH_2}CH_2CH_3$), 1.84 (d, *J* = 7.1 Hz, 2H, C12-O$CH_2\underline{CH_2}CH_3$), 1.06 (t, *J* = 7.4 Hz, 3H, C12-O$CH_2CH_2\underline{CH_3}$).

2.36. (Z)-3-(4-butoxyphenyl)-2-(4-(trifluoromethoxy)phenyl)acrylonitrile (1a2n)

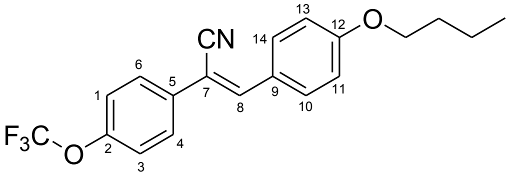

White powder; yield 50%. M.p.62-64°C. ^{1}H NMR (500 MHz, $CDCl_3$) δ 7.88 (d, *J* = 8.7 Hz, 2H, C10, C14-H), 7.67 (d, *J* = 8.7 Hz, 2H, C4, C6-H), 7.43 (s, 1H, C8-H), 7.26 (d, *J* = 5.0 Hz, 2H, C11, C13-H), 6.97 (d, *J* = 8.7 Hz, 2H, C1, C3-H), 4.04 (t, *J* = 6.4 Hz, 2H, C12-O$\underline{CH_2}(CH_2)_2CH_3$), 1.85–1.76 (m, 2H, C12-O$CH_2\underline{CH_2}CH_2CH_3$), 1.55–1.49 (m, 2H, C12-O$(CH_2)_2\underline{CH_2}CH_3$), 0.99 (t, *J* = 7.4 Hz, 3H, C12-O$(CH_2)_3\underline{CH_3}$).

2.37. (Z)-3-(4-(pentyloxy)phenyl)-2-(4-(trifluoromethoxy)phenyl)acrylonitrile (1a2o)

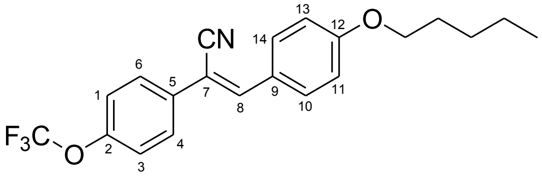

White flaky crystal; yield 61%. M.p.70-72°C. ^{1}H NMR (500 MHz, $CDCl_3$) δ 7.87 (d, *J* = 8.7 Hz, 2H, C10, C14-H), 7.67 (d, *J* = 8.7 Hz, 2H, C4, C6-H), 7.43 (s, 1H, C8-H), 7.26 (d, *J* = 5.0 Hz, 2H, C11, C13-H), 6.97 (d, *J* = 8.7 Hz, 2H, C1, C3-H), 4.03 (t, *J* = 6.5 Hz, 2H, C12-O$\underline{CH_2}(CH_2)_3CH_3$), 1.85 – 1.78 (m, 2H, C12-O$CH_2\underline{CH_2}(CH_2)_2CH_3$), 1.49 – 1.37 (m, 4H, C12-O$CH_2CH_2\underline{(CH_2)_2}CH_3$), 0.95 (t, *J* = 7.1 Hz, 3H, C12-O$CH_2(CH_2)_3\underline{CH_3}$).

2.38. (Z)-3-(4-(hexyloxy)phenyl)-2-(4-(trifluoromethoxy)phenyl)acrylonitrile (1a2p)

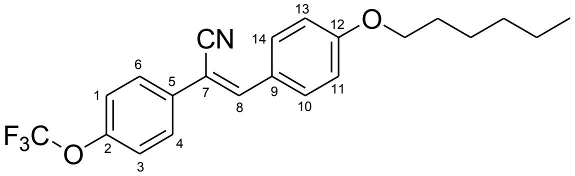

White powder; yield 47%. M.p.68-70°C. ^{1}H NMR (500 MHz, $CDCl_3$) δ 7.87 (d, *J* = 8.7 Hz, 2H, C10, C14-H), 7.67 (d, *J* = 8.7 Hz, 2H, C4, C6-H), 7.43 (s, 1H, C8-H), 7.26 (d, *J* = 4.8 Hz, 2H, C11, C13-H), 6.97 (d, *J* = 8.7 Hz, 2H, C1, C3-H), 4.02 (t, *J* = 6.5 Hz, 2H, C12-O$\underline{CH_2}(CH_2)_4CH_3$), 1.85 – 1.77 (m, 2H, C12-O$CH_2\underline{CH_2}(CH_2)_3CH_3$), 1.48 (s, 2H, C12-O$(CH_2)_2\underline{CH_2}(CH_2)_2CH_3$), 1.35 (d, *J* = 3.3 Hz, 4H, C12-O$(CH_2)_3\underline{(CH_2)_2}CH_3$), 0.92 (s, 3H, C12-O$(CH_2)_5\underline{CH_3}$).

2.39. (Z)-3-m-tolyl-2-(4-(trifluoromethoxy)phenyl)acrylonitrile (1a2q)

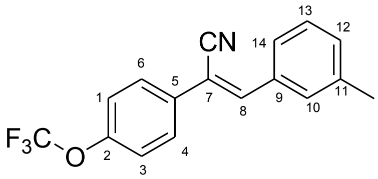

White needle crystal; yield 50%. M.p.70-72°C. ^{1}H NMR (300 MHz, $CDCl_3$) δ 7.73 (s, 2H, C4, C6-H), 7.71 (s, 1H, C8-H), 7.70 – 7.68 (m, 1H, C14-H), 7.51 (s, 1H, C10-H), 7.39 (s, 1H, C13-H), 7.33 (d, *J* = 0.8 Hz, 1H, C12-H), 7.30 (d, *J* = 1.1 Hz, 2H, C1, C3-H), 2.44 (s, 3H, C11-$\underline{CH_3}$).

2.40. (Z)-3-mesityl-2-(4-(trifluoromethoxy)phenyl)acrylonitrile (1a2r)

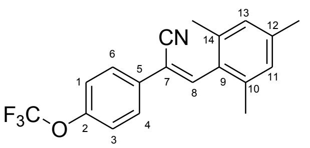

White powder; yield 59%. M.p.78-80°C. ^{1}H NMR (300 MHz, $CDCl_3$) δ 7.75 (d, *J* = 9.0 Hz, 2H, C4, C6-H), 7.70 (s, 1H, C8-H), 7.37 – 7.31 (m, 2H, C1, C3-H), 6.97 (s, 2H, C11, C13-H), 2.34 (s, 3H, C12-$\underline{CH_3}$), 2.32 (s, 6H, C10, C14-$\underline{CH_3}$).

2.41. (Z)-3-(4-morpholinophenyl)-2-(4-(trifluoromethoxy)phenyl)acrylonitrile (1a2s)

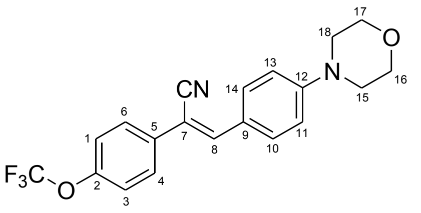

Yellow powder; yield 73%. M.p.80-82°C. ^{1}H NMR (300 MHz, $CDCl_3$) δ 7.89 (d, *J* = 8.9 Hz, 2H, C10, C14-H), 7.68 (d, *J* = 8.8 Hz, 2H, C4, C6-H), 7.42 (s, 1H, C8-H), 7.30 (s, 2H, C11, C13-H), 6.95 (d, *J* = 8.9 Hz, 2H, C1, C3-H), 3.95 – 3.86 (m, 4H, C16, C17-H), 3.39 – 3.29 (m, 4H, C15, C18-H).

2.42. (Z)-3-(4-(pyrrolidin-1-yl)phenyl)-2-(4-(trifluoromethoxy)phenyl)acrylonitrile (1a2t)

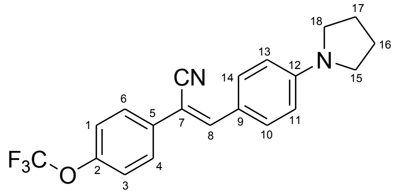

Yellow powder; yield 59%. M.p.118-120°C. ^{1}H NMR (300 MHz, $CDCl_3$) δ 7.87 (d, *J* = 8.8 Hz, 2H, C10, C14-H), 7.66 (d, *J* = 8.9 Hz, 2H, C4, C6-H), 7.38 (s, 1H, C8-H), 7.27 (d, *J* = 8.8 Hz, 2H, C11, C13-H), 6.61 (d, *J* = 8.8 Hz, 2H, C1, C3-H), 3.51 (s, 2H, C15-H), 3.40 (s, 2H, C18-H), 2.07 (t, *J* = 6.5 Hz, 4H, C16, C17-H). HRMS (ESI) m/z calcd for $C_{20}H_{18}F_3N_2O$ ($[M+H]^+$) 359.13657, found 359.13647.

2.43. (Z)-3-(4-(piperidin-1-yl)phenyl)-2-(4-(trifluoromethoxy)phenyl)acrylonitrile (1a2u)

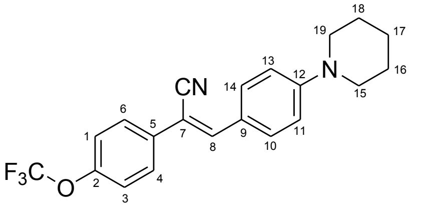

Yellow powder; yield 73%. M.p.98-100°C. ^{1}H NMR (300 MHz, $CDCl_3$) δ 7.86 (d, *J* = 9.0 Hz, 2H, C10, C14-H), 7.67 (dd, *J* = 6.8, 2.1 Hz, 2H, C4, C6-H), 7.39 (s, 1H, C8-H), 7.29 (s, 2H, C11, C13-H), 6.93 (d, *J* = 9.0 Hz, 2H, C1, C3-H), 3.37 (d, *J* = 5.7 Hz, 4H, C15, C19-H), 1.69 (s, 6H, C16, C17, C18-H).

2.44. (Z)-3-(furan-2-yl)-2-(4-(trifluoromethoxy)phenyl)acrylonitrile (1a3a)

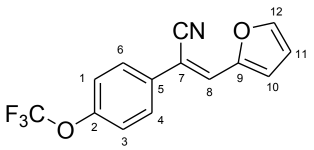

Light yellow powder; yield 21%. M.p.68-70°C. ^{1}H NMR (500 MHz, DMSO-d6) δ 8.01 (d, *J* = 1.4 Hz, 1H, C12-H), 7.92 (s, 1H, C8-H), 7.87 – 7.82 (m, 2H, C4, C6-H), 7.47 (d, *J* = 8.2 Hz, 2H, C1, C3-H), 7.18 (d, *J* = 3.5 Hz, 1H, C10-H), 6.77 (dd, *J* = 3.5, 1.8 Hz, 1H, C11-H).

2.45. (Z)-3-(thiophen-2-yl)-2-(4-(trifluoromethoxy)phenyl)acrylonitrile (1a3b)

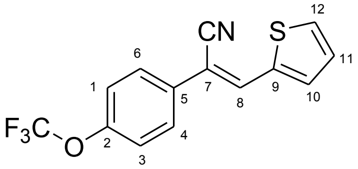

Light yellow powder; yield 33%. M.p.100-102°C. ^{1}H NMR (300 MHz, $CDCl_3$) δ 7.71 (s, 2H, C4, C6-H), 7.67 (d, *J* = 3.9 Hz, 2H, C8, C12-H), 7.60 (d, *J* = 5.0 Hz, 1H, C10-H), 7.32 (s, 2H, C1, C3-H), 7.19 (s, 1H, C11-H).

2.46. (Z)-3-(naphthalen-1-yl)-2-(4-(trifluoromethoxy)phenyl)acrylonitrile (1a3c)

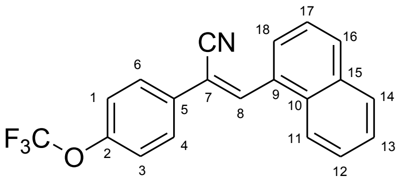

White powder; yield 46%. M.p.65-67°C. ^{1}H NMR (500 MHz, $CDCl_3$) δ 8.28 (s, 1H, C18-H), 8.09 (d, *J* = 7.1 Hz, 1H, C8-H), 7.97 (d, *J* = 7.3 Hz, 2H, C11, C14-H), 7.94 – 7.92 (m, 1H, C17-H), 7.81 (d, *J* = 8.7 Hz, 2H, C11, C16-H), 7.58 (d, *J* = 5.1 Hz, 3H, C4, C6, C13-H), 7.35 (d, *J* = 8.3 Hz, 2H, C1, C3-H). ^{13}C NMR (75 MHz, $CDCl_3$) δ 149.98 (s, C8), 142.97 (s, C2), 141.04 (s, C15), 133.60 (s, C10), 132.91 (s, C9), 131.94 – 130.75 (m, C16), 130.75 – 130.54 (m, C5), 128.95 (d, *J* = 16.7 Hz, C18), 127.67 (d, *J* = 17.0 Hz, C14), 127.12 (s, C11, C13), 126.94 – 126.63 (m, C17), 126.54 (s, C12), 125.74 (t, *J* = 51.7 Hz, C4, C6), 123.24 (s, C1, C3), 121.48 (s, C2-OCF_3), 117.34 (s, C7-CN), 113.98 (s, C7). HRMS (ESI) m/z calcd for $C_{20}H_{13}F_3NO$ ($[M+H]^+$) 340.09438, found 340.09445.

2.47. (Z)-3-(4-methoxyphenyl)-2-(3-(trifluoromethoxy)phenyl)acrylonitrile (1b2j)

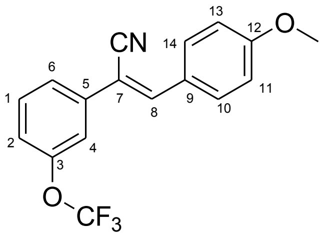

Light yellow powder; yield 40%. M.p.200-202°C. ^{1}H NMR (300 MHz, $CDCl_3$) δ 7.93 (d, *J* = 8.8 Hz, 2H, C10, C14-H), 7.61 (s, 1H, C8-H), 7.50 (t, *J* = 3.9 Hz, 3H, C1, C11, C13-H), 7.25 (d, *J* = 8.2 Hz, 1H, C6-H), 7.02 (d, *J* = 8.9 Hz, 2H, C4, C2-H), 3.90 (s, 3H, C12-OCH_3). ^{13}C NMR (75 MHz, $CDCl_3$) δ 161.82 (s, C12), 149.76 (s, C3), 143.02 (s, C8), 136.99 (s, C5), 131.37 (s, C10, C14), 130.30 (s, C1), 125.96 (s, C9), 124.08 (s, C6), 120.74 (s, C2), 118.68 (s, C3-OCF_3), 118.20 (s, C7-CN), 117.89 (s, C4), 114.43 (s, C11, C13), 107.08 (s, C7), 55.33 (s, C12-OCH_3).

2.48. (Z)-3-(4-propoxyphenyl)-2-(3-(trifluoromethoxy)phenyl)acrylonitrile (1b2k)

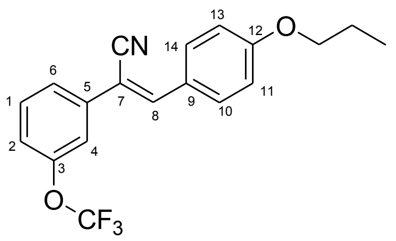

White powder; yield 25%. M.p.68-70°C. ^{1}H NMR (300 MHz, $CDCl_3$) δ 7.92 (d, *J* = 8.9 Hz, 2H, C10, C14-H), 7.62 (d, *J* = 8.0 Hz, 1H, C8-H), 7.50 (d, *J* = 5.7 Hz, 2H, C11, C13-H), 7.46 (s, 1H, C1-H), 7.25 (d, *J* = 9.1 Hz, 1H, C6-H), 7.02 (s, 1H, C4-H), 6.99 (s, 1H, C2-H), 4.02 (t, *J* = 6.5 Hz, 2H, C12-O$CH_2CH_2CH_3$), 1.87 (dd, *J* = 14.1, 6.9 Hz, 2H, C12-O$CH_2CH_2CH_3$), 1.08 (t, *J* = 7.4 Hz, 3H, C12-O$CH_2CH_2CH_3$).

2.49. (Z)-3-(4-(dimethylamino)phenyl)-2-(4-methoxyphenyl)acrylonitrile (1e2b)

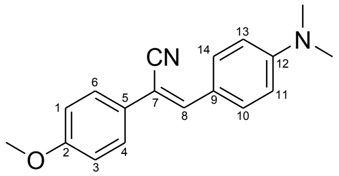

Yellow powder; yield 60%. M.p.130-132°C. ^{1}H NMR (300 MHz, $CDCl_3$) δ 7.82 (d, *J* = 8.8 Hz, 2H, C10, C14-H), 7.57 (d, *J* = 8.7 Hz, 2H, C4, C6-H), 7.29 (s, 1H, C8-H), 6.95 (d, *J* = 8.7 Hz, 2H, C1, C3-H), 6.70 (d, *J* = 8.8 Hz, 2H, C11, C13-H), 3.85 (s, 3H, C2-OCH_3), 3.44 (dd, *J* = 13.9, 6.9 Hz, 6H, C12-N(CH_3)$_2$). ^{13}C NMR (75 MHz, $CDCl_3$) δ 159.67 (s, C2), 151.51 (s, C12), 140.78 (s, C8), 130.94 (s, C10, C14), 128.23 (s, C4, C6), 126.77 (s, C5), 121.99 (s, C9), 119.50 (s, C7-CN), 114.36 (s, C1, C3), 111.72 (s, C11, C13), 104.59 (s, C7), 55.42 (s, C2-OCH_3), 40.04 (s, C12-N(CH_3)$_2$). HRMS (ESI) m/z calcd for $C_{18}H_{19}N_2O$ ([M+H]$^+$) 279.14919, found 279.14902.

2.50. (Z)-3-(4-(diethylamino)phenyl)-2-(4-methoxyphenyl)acrylonitrile (1e2c)

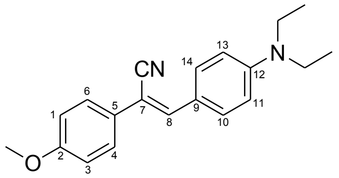

Yellow powder; yield 60%. M.p.130-132°C. ^{1}H NMR (300 MHz, $CDCl_3$) δ 7.84 (d, *J* = 8.8 Hz, 2H, C10, C14-H), 7.58 (d, *J* = 8.8 Hz, 2H, C4, C6-H), 7.31 (s, 1H, C8-H), 6.95 (d, *J* = 8.8 Hz, 2H, C1, C3-H), 6.74 (s, 2H, C11, C13-H), 3.86 (d, *J* = 8.8 Hz, 4H, C12-N(CH_2CH_3)$_2$), 3.07 (s, 3H, C2-OCH_3), 1.27 (t, *J* = 7.0 Hz, 6H, C12-N(CH_2CH_3)$_2$). ^{13}C NMR (75 MHz, $CDCl_3$) δ 159.58 (s, C2), 149.17 (s, C12), 140.81 (s, C8), 131.29 (s, C10, C14), 128.36 (s, C4, C6), 126.70 (s, C5), 121.19 (s, C9), 119.70 (s, C7-CN), 114.36 (s, C1, C3), 111.22 (s, C11, C13), 103.72 (s, C7), 55.43 (s, C2-OCH_3), 44.50 (s, C12-N(CH_2CH_3)$_2$), 12.66 (s, C12-N(CH_2CH_3)$_2$).

2.51. (Z)-3-(4-(diethylamino)phenyl)-2-(4-(trifluoromethyl)phenyl)acrylonitrile (1f2b)

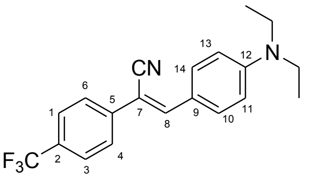

Yellow powder; yield 63%. M.p.108-110°C. ^{1}H NMR (300 MHz, $CDCl_3$) δ 7.89 (d, *J* = 8.9 Hz, 2H, C10, C14-H), 7.75 (d, *J* = 8.2 Hz, 2H, C1, C3-H), 7.66 (d, *J* = 8.5 Hz, 2H, C4, C6-H), 7.47 (s, 1H, C8-H), 6.73 (d, *J* = 7.7 Hz, 2H, C11, C13-H), 3.50 – 3.41 (m, 4H, C12-N(CH_2CH_3)$_2$), 1.25 (t, *J* = 7.8 Hz, 6H, C12-N(CH_2CH_3)$_2$).

2.52. (Z)-2-(4-(trifluoromethyl)phenyl)-3-phenylacrylonitrile (1f2c)

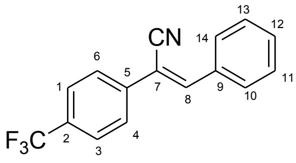

White powder; yield 40%. M.p.132-134°C. ^{1}H NMR (300 MHz, $CDCl_3$) *δ* 8.02 – 7.90 (m, 2H, C1, C3-H), 7.83 (d, *J* = 8.3 Hz, 2H, C4, C6-H), 7.74 (d, *J* = 8.1 Hz, 2H, C10, C14-H), 7.64 (s, 1H, C8-H), 7.62 – 7.47 (m, 3H, C11, C12, C13-H).

2.53. (Z)-2-(4-(trifluoromethyl)phenyl)-3-(4-fluorophenyl)acrylonitrile (1f2d)

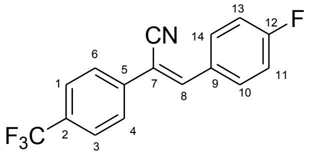

White powder; yield 12%. M.p.110-112°C. ^{1}H NMR (300 MHz, $CDCl_3$) δ 7.96 (dd, J = 8.9, 5.2 Hz, 2H, C1, C3-H), 7.81 (d, J = 8.6 Hz, 2H, C4, C6-H), 7.73 (d, J = 8.3 Hz, 2H, C10, C14-H), 7.59 (s, 1H, C8-H), 7.21 (t, J = 8.6 Hz, 2H, C11, C13-H).

2.54. (Z)-3-(4-chlorophenyl)-2-(4-(trifluoromethyl)phenyl)acrylonitrile (1f2e)

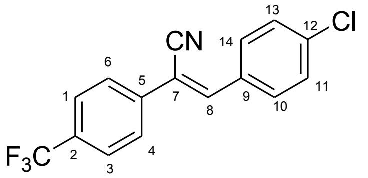

Light pink solid; yield 26%. M.p.78-80°C. ^{1}H NMR (300 MHz, $CDCl_3$) δ 7.96 (dd, J = 8.9, 5.2 Hz, 2H, C1, C3-H), 7.81 (d, J = 8.6 Hz, 2H, C4, C6-H), 7.73 (d, J = 8.3 Hz, 2H, C10, C14-H), 7.59 (s, 1H, C8-H), 7.21 (t, J = 8.6 Hz, 2H, C11, C13-H).

2.55. (Z)-3-(4-bromophenyl)-2-(4-(trifluoromethyl)phenyl)acrylonitrile (1f2f)

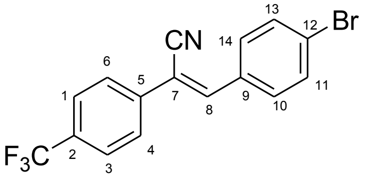

White flaky crystal; yield 27%. M.p.66-70°C. ^{1}H NMR (300 MHz, $CDCl_3$) δ 7.79 (d, J = 7.8 Hz, 4H, C1, C3, C4, C6-H), 7.72 (d, J = 8.3 Hz, 2H, C11, C13-H), 7.63 (d, J = 8.4 Hz, 2H, C10, C14-H), 7.54 (s, 1H, C8-H).

2.56. (Z)-2-(4-(trifluoromethyl)phenyl)-3-(2-fluorophenyl)acrylonitrile (1f2g)

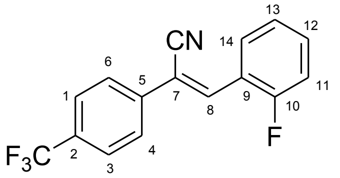

White flaky crystal; yield 27%. M.p.98-100°C. ^{1}H NMR (300 MHz, $CDCl_3$) δ 8.21 – 8.15 (m, 1H, C8-H), 8.06 (s, 1H, C14-H), 7.81 (d, J = 8.2 Hz, 2H, C1, C3-H), 7.70 (d, J = 8.4 Hz, 2H, C4, C6-H), 7.49 – 7.42 (m, 1H, C11-H), 7.09 (t, J = 7.6 Hz, 1H, C13-H), 6.94 (dd, J = 18.2, 7.9 Hz, 1H, C12-H).

2.57. (Z)-2-(4-(trifluoromethyl)phenyl)-3-(3-fluorophenyl)acrylonitrile (1f2h)

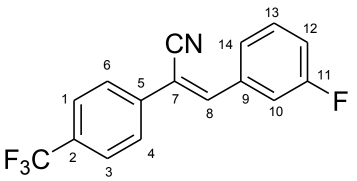

White flaky crystal; yield 54%. M.p.158-160°C. ^{1}H NMR (300 MHz, $CDCl_3$) δ 7.80 (d, J = 8.3 Hz, 2H, C1, C3-H), 7.76 – 7.70 (m, 2H, C4, C6-H), 7.70 – 7.60 (m, 2H, C10, C14-H), 7.57 (s, 1H, C8-H), 7.48 (td, J = 8.0, 5.9 Hz, 1H, C13-H), 7.23 – 7.14 (m, 1H, C12-H).

2.58. (Z)-2-(4-(trifluoromethyl)phenyl)-3-(2-fluorophenyl)acrylonitrile (1f2i)

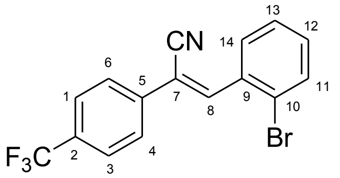

White powder; yield 43%. M.p.110-112°C. ^{1}H NMR (300 MHz, $CDCl_3$) δ 8.09 (dd, J = 7.8, 1.5 Hz, 1H, C8-H), 7.93 (s, 1H, C11-H), 7.88 – 7.80 (m, 2H, C1, C3-H), 7.75 (s, 1H, C14-H), 7.74 – 7.67 (m, 2H, C4, C6-H), 7.51 – 7.44 (m, 1H, C13-H), 7.34 (td, J = 7.7, 1.6 Hz, 1H, C12-H). ^{13}C NMR (75 MHz, $CDCl_3$) δ 143.05 (s, C8), 137.29 (s, C5), 133.51 (s, C2), 133.25 (s, C11),

131.96 (s, C9), 129.68 (s, C14), 127.97 (s, C12), 127.30 (s, C13), 126.63 (s, C10), 126.29 (s, C4, C6), 126.18 (s, C1, C3), 125.27 (s, C2-CF_3), 116.63 (s, C7), 113.64 (s, C7-CN).

2.59. (Z)-3-(2-chloro-6-fluorophenyl)-2-(4-(trifluoromethyl)phenyl)acrylonitrile (1f2j)

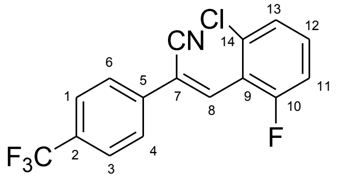

White powder; yield 36%. M.p.110-112°C. ^{1}H NMR (300 MHz, $CDCl_3$) δ 7.83 (d, J = 8.1 Hz, 2H, C1, C3-H), 7.72 (d, J = 8.2 Hz, 2H, C4, C6-H), 7.61 (s, 1H, C8-H), 7.33 (t, J = 8.2 Hz, 1H, C13-H), 7.09 (d, J = 8.1 Hz, 1H, C11-H), 6.93 (d, J = 8.4 Hz, 1H, C12-H).

2.60. (Z)-2,3-bis(4-(trifluoromethyl)phenyl)acrylonitrile (1f2k)

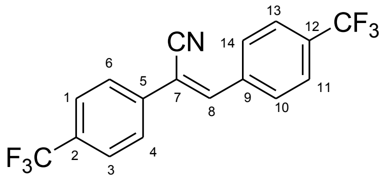

Light pink solid; yield 61%. M.p.64-68°C. ^{1}H NMR (300 MHz, $CDCl_3$) δ 8.02 (d, J = 8.2 Hz, 2H, C4, C6-H), 7.84 (d, J = 8.2 Hz, 2H, C10, C14-H), 7.76 (t, J = 7.0 Hz, 4H, C1, C3, C11, C13-H), 7.67 (s, 1H, C8-H). ^{13}C NMR (75 MHz, $CDCl_3$) δ 142.08 (s, C8), 137.27 (s, C5), 136.47 (s, C2), 129.62 (s, C9), 127.15 (s, C12), 126.94 (s, C10, C14), 126.25 (s, C11, C13), 126.20 (s, C4, C6), 126.12 (s, C1, C3), 126.02 (s, C2, C12-CF_3), 116.81 (s, C7-CN), 113.23 (s, C7). HRMS (ESI) m/z calcd for $C_{17}H_{10}F_6N$ ($[M+H]^+$) 342.07119, found 342.07150.

2.61. (Z)-3-(3-(trifluoromethyl)phenyl)-2-(4-(trifluoromethyl)phenyl)acrylonitrile (1f2l)

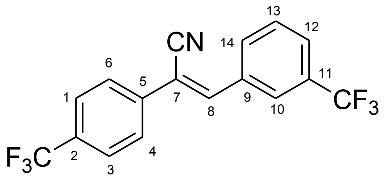

Light pink solid; yield 23%. M.p.62-64°C. ^{1}H NMR (300 MHz, $CDCl_3$) δ 8.22 (d, J = 7.8 Hz, 1H, C8-H), 8.04 (s, 1H, C10-H), 7.82 (d, J = 8.2 Hz, 2H, C1, C3-H), 7.74 (d, J = 7.9 Hz, 3H, C4, C6, C14-H), 7.71 – 7.61 (m, 2H, C12, C13-H).

2.62. (Z)-3-(2-(trifluoromethyl)phenyl)-2-(4-(trifluoromethyl)phenyl)acrylonitrile (1f2m)

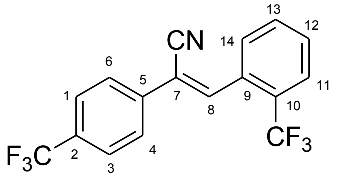

White power; yield 32%. M.p.76-78°C. ^{1}H NMR (300 MHz, $CDCl_3$) δ 8.06 (d, J = 7.8 Hz, 1H, C8-H), 7.98 (d, J = 2.1 Hz, 1H, C11-H), 7.81 (dd, J = 11.5, 3.3 Hz, 3H, C1, C3, C14-H), 7.72 (dd, J = 12.6, 8.1 Hz, 3H, C4, C6, C13-H), 7.59 (t, J = 7.6 Hz, 1H, C12-H).

2.63. (Z)-2-(4-(trifluoromethyl)phenyl)-3-p-tolylacrylonitrile (1f2n)

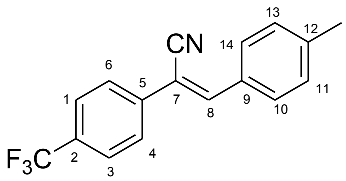

Light yellow powder; yield 56%. M.p.136-138°C. ^{1}H NMR (300 MHz, $CDCl_3$) δ 7.82 (dd, *J* = 13.4, 8.2 Hz, 4H, C1, C3, C4, C6-H), 7.73 (t, *J* = 8.9 Hz, 2H, C10, C14-H), 7.60 (s, 1H, C8-H), 7.31 (d, *J* = 8.1 Hz, 2H, C11, C13-H), 2.44 (s, 3H, C12-CH_3). ^{13}C NMR (75 MHz, $CDCl_3$) δ 144.18 (s, C8), 141.98 (s, C12), 138.23 (s, C5), 131.14 (s, C2), 129.83 (s, C11, C13), 129.60 (s , C10, C14), 126.23(s, C9) 126.06 (s, C4, C6), 126.01 (s, C1, C3), 122.06 (s, C2-CF_3), 117.66 (s, C7-CN), 109.11 (s, C7), 21.58 (s, C12-CH_3). HRMS (ESI) m/z calcd for $C_{17}H_{13}F_3N$ ($[M+H]^+$) 288.09946, found 288.09973.

2.64. (Z)-2-(4-(trifluoromethyl)phenyl)-3-m-tolylacrylonitrile (1f2o)

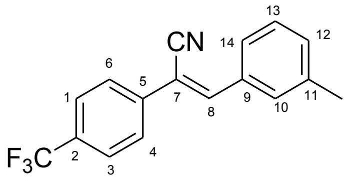

Light pink powder; yield 47%. M.p.72-74°C. ^{1}H NMR (300 MHz, $CDCl_3$) δ 7.83 (s, 1H, C8-H), 7.80 (s, 2H, C1, C3-H), 7.74 (s, 3H, C4, C6, C14-H), 7.61 (s, 1H, C10-H), 7.41 (s, 1H, C13-H), 7.32 (d, *J* = 9.1 Hz, 1H, C12-H), 2.45 (s, 3H, C11-CH_3). ^{13}C NMR (75 MHz, $CDCl_3$) δ 144.38 (s, C8), 138.87 (s, C11), 138.11 (s, C9) 133.22 (s, C5), 132.06 (s, C2), 130.19 (s, C12), 129.00 (s, C10), 126.64 (s, C13), 126.32 (s, C14), 126.09 (s, C4, C6),126.04 (s, C1, C3) 125.46 (s, C2-CF_3), 117.49 (s, C7-CN), 110.09 (s, C7), 21.34 (s, C11-CH_3).

2.65. (Z)-3-(4-ethylphenyl)-2-(4-(trifluoromethyl)phenyl)acrylonitrile (1f2p)

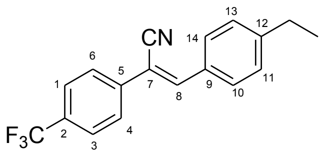

White flaky crystal; yield 41%. M.p.124-126°C. ^{1}H NMR (300 MHz, $CDCl_3$) δ 7.87 (d, *J* = 8.2 Hz, 2H, C10, C14-H), 7.80 (d, *J* = 8.2 Hz, 2H, C1, C3-H), 7.71 (d, *J* = 8.5 Hz, 2H, C4, C6-H), 7.60 (s, 1H, C8-H), 7.34 (d, *J* = 8.2 Hz, 2H, C11, C13-H), 2.73 (q, *J* = 7.5 Hz, 2H, C12-CH_2CH_3), 1.27 (d, *J* = 7.6 Hz, 3H, C12-CH_2CH_3).

2.66. (Z)-2-(4-(trifluoromethyl)phenyl)-3-(4-isopropylphenyl)acrylonitrile (1f2q)

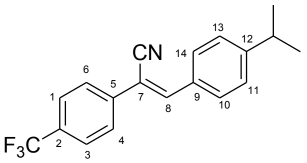

White flaky crystal; yield 60%. M.p.110-112°C. ^{1}H NMR (300 MHz, $CDCl_3$) δ 7.89 (d, *J* = 8.2 Hz, 2H, C10, C14-H), 7.81 (d, *J* = 8.1 Hz, 2H, C1, C3-H), 7.72 (d, *J* = 8.4 Hz, 2H, C4, C6-H), 7.61 (s, 1H, C8-H), 7.38 (d, *J* = 8.3 Hz, 2H, C11, C13-H), 3.05 – 2.94 (m, 1H, C12-$CH(CH_3)_2$), 1.31 (d, *J* = 6.9 Hz, 6H, C12-$CH(CH_3)_2$).

2.67. (Z)-2-(4-(trifluoromethyl)phenyl)-3-mesitylacrylonitrile (1f2r)

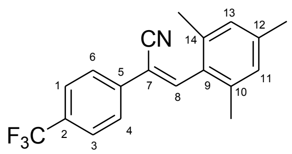

White powder; yield 45%. M.p.144-146°C. ^{1}H NMR (300 MHz, $CDCl_3$) δ 7.85 (s, 1H, C8-H), 7.82 (s, 2H, C1, C3-H), 7.75 (d, *J* = 8.6 Hz, 2H, C4, C6-H), 6.98 (s, 2H, C11, C13-H), 2.33 (d, *J* = 4.3 Hz, 9H, C10, C12, C14-H).

2.68. (Z)-2-(4-(trifluoromethyl)phenyl)-3-(4-methoxyphenyl)acrylonitrile (1f2s)

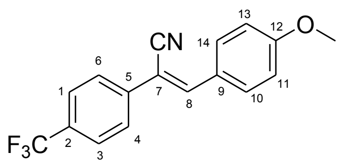

Light yellow powder; yield 53%. M.p.120-122°C. ^{1}H NMR (300 MHz, $CDCl_3$) δ 7.95 (d, *J* = 8.8 Hz, 2H, C10, C14-H), 7.79 (d, *J* = 8.3 Hz, 2H, C1,C3-H), 7.71 (d, *J* = 8.4 Hz, 2H, C4, C6-H), 7.56 (s, 1H, C8-H), 7.02 (d, *J* = 8.8 Hz, 2H, C11, C13-H), 3.91 (s, 3H, C12-OCH_3). ^{13}C NMR (75 MHz, $CDCl_3$) δ 162.04 (s, C12), 143.70 (s, C8), 138.41 (m, C5, C2), 131.59 (s, C10, C14), 131.58 (s, C9), 130.75 (s, C4, C6), 128.48 (s, C1, C3), 126.04 (s, C2-CF_3), 118.00 (s, C11, C13), 114.58 (s, C7-CN), 107.20 (s, C7), 55.47 (s, C12-OCH_3). HRMS (ESI) m/z calcd for $C_{17}H_{13}F_3NO$ ($[M+H]^+$) 304.09438, found 304.09406.

2.69. (Z)-2-(4-(trifluoromethyl)phenyl)-3-(3-methoxyphenyl)acrylonitrile (1f2t)

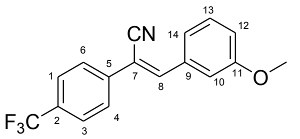

White powder; yield 45%. M.p.100-102°C. ^{1}H NMR (300 MHz, $CDCl_3$) δ 7.82 (d, *J* = 8.3 Hz, 2H, C1, C3-H), 7.73 (d, *J* = 8.3 Hz, 2H, C4, C6-H), 7.61 (s, 1H, C8-H), 7.56 (s, 1H, C13-H), 7.44 (dt, *J* = 15.7, 7.7 Hz, 2H, C10, C14-H), 7.06 (d, *J* = 7.7 Hz, 1H, C12-H), 3.91 (s, 3H, C11-OCH_3). ^{13}C NMR (75 MHz, $CDCl_3$) δ 160.01 (s, C11), 144.13 (s, C8), 137.97 (s, C5), 134.45 (s, C2), 130.07 (s, C9), 128.38 (s, C13), 126.56 – 126.44 (m, C4, C6), 126.44 – 125.39 (m, C1, C3), 122.49 (s, C2-CF_3), 117.67 (s, C14), 117.47 (s, C12), 114.98 (s, C7-CN), 113.79 (s, C10), 110.50 (s, C7), 55.43 (s, C11-OCH_3).

2.70. (Z)-2-(4-(trifluoromethyl)phenyl)-3-(3,4,5-trimethoxyphenyl)acrylonitrile (1f2u)

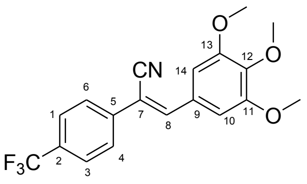

Light yellow powder; yield 31%. M.p. > 240°C. ^{1}H NMR (300 MHz, $CDCl_3$) δ 7.81 (d, *J* = 8.5 Hz, 2H, C1, C3-H), 7.73 (d, *J* = 8.2 Hz, 2H, C4, C6-H), 7.55 (s, 1H, C8-H), 7.25 (s, 2H, C10, C14-H), 3.96 (s, 9H, C11, C12, C13-OCH_3).

2.71. (Z)-2-(4-(trifluoromethyl)phenyl)-3-(4-phenoxyphenyl)acrylonitrile (1f2v)

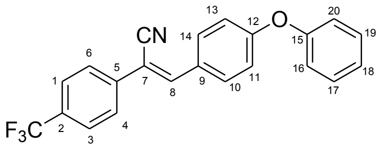

White flaky crystal; yield 43%. M.p.128-130°C. ^{1}H NMR (300 MHz, $CDCl_3$) δ 7.94 (d, *J* = 8.9 Hz, 2H, C10, C14-H), 7.79 (d, *J* = 8.6 Hz, 2H, C1, C3-H), 7.71 (d, *J* = 8.3 Hz, 2H, C17, C19-H), 7.56 (s, 1H, C8-H), 7.47 (d, *J* = 6.7 Hz, 2H, C4, C6-H), 7.44 (s, 2H, C11, C13-H), 7.40 (d, *J* = 3.3 Hz, 1H, C18-H), 7.09 (d, *J* = 8.8 Hz, 2H, C16, C20-H).

2.72. (2Z, 2'Z)-3,3'-(1,4-phenylene)bis(2-(4-(trifluoromethyl)phenyl)acrylonitrile) (1f2w)

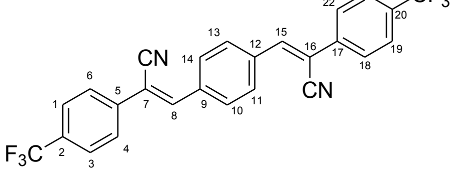

Light yellow powder; yield 41%. M.p. > 240°C. ^{1}H NMR (300 MHz, $CDCl_3$) δ 8.07 (s, 4H, C1, C3, C19, C21-H), 7.86 (d, *J* = 8.3 Hz, 4H, C10, C14, C11, C13-H), 7.76 (d, *J* = 8.3 Hz, 4H, C4, C6, C18, C22-H), 7.66 (s, 2H, C8, C15-H). HRMS (ESI) m/z calcd for $C_{26}H_{15}F_6N_2$ ($[M+H]^+$) 469.11339, found 469.11319.

2.73. (Z)-2-(4-(trifluoromethyl)phenyl)-3-(furan-2-yl)acrylonitrile (1f3a)

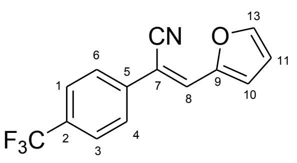

Brown powder; yield 40%. M.p.110-112°C. ^{1}H NMR (300 MHz, $CDCl_3$) δ 7.78 (d, *J* = 8.2 Hz, 2H, C1, C3-H), 7.72 (s, 1H, C13-H), 7.72 – 7.63 (m, 2H, C4, C6-H), 7.49 (s, 1H, C8-H), 7.29 (s, 1H, C10-H), 6.69 – 6.61 (m, 1H, C11-H).

2.74. (Z)-2-(4-(trifluoromethyl)phenyl)-3-(thiophen-2-yl)acrylonitrile (1f3b)

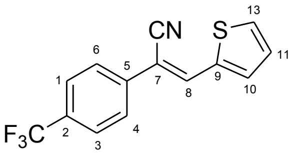

Light yellow powder; yield 38%. M.p.168-170°C. ^{1}H NMR (300 MHz, $CDCl_3$) δ 7.78 (d, *J* = 8.2 Hz, 2H, C1, C3-H), 7.72 (s, 1H, C13-H), 7.72 – 7.63 (m, 2H, C4, C6-H), 7.49 (s, 1H, C8-H), 7.29 (s, 1H, C10-H), 6.69 – 6.61 (m, 1H, C11-H). ^{13}C NMR (75 MHz, $CDCl_3$) δ 137.53 (s, C5), 135.94 (s, C8), 133.36 (s, C10), 131.12 (s, C9), 130.60 (s, C12), 128.07 (s, C11), 126.12 (s, C4, C6), 126.07 (s, C13), 125.96 (m, C1, C3), 122.03 (s, C2-CF_3), 117.60 (s, C7-CN), 106.83 (s, C7).

2.75. (Z)-2-(4-(trifluoromethyl)phenyl)-3-(pyridin-2-yl)acrylonitrile (1f3c)

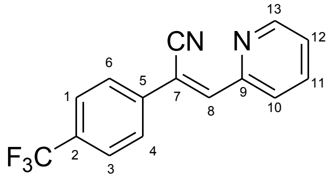

White powder; yield 43%. M.p.128-130°C. ^{1}H NMR (300 MHz, $CDCl_3$) δ 8.81 (d, *J* = 4.6 Hz, 1H, C13-H), 7.97 (d, *J* = 7.9 Hz, 1H, C10-H), 7.91 (s, 1H, C11-H), 7.88 (s, 1H, C3-H), 7.86 (d, *J* = 1.8 Hz, 1H, C1-H), 7.77 (s, 1H, C12-H), 7.74 (s, 2H, C4, C6-H), 7.39 (d, *J* = 1.0 Hz, 1H, C8-H). ^{13}C NMR (75 MHz, $CDCl_3$) δ 151.67 (s, C9), 150.15 (s, C11), 142.89 (s, C8), 137.54 (s, C13), 136.85 (s, C5), 126.82 (s, C2), 126.73 (s, C4, C6), 126.14 (s, C1, C3), 126.09 (s, C2-CF_3), 124.73 (s, C14), 124.46 (s, C12), 116.86 (s, C7-CN), 113.61 (s, C7). HRMS (ESI) m/z calcd for $C_{15}H_{10}F_3N_2$ ($[M+H]^+$) 275.07906, found 275.07913.

2.76. (Z)-2-(4-(trifluoromethyl)phenyl)-3-(naphthalen-1-yl)acrylonitrile (1f3d)

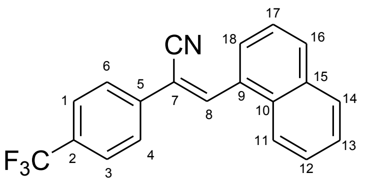

Light yellow powder; yield 39%. M.p.112-114°C. ^{1}H NMR (300 MHz, $CDCl_3$) δ 8.41 (s, 1H, C18-H), 8.15 (d, *J* = 7.3 Hz, 1H, C8-H), 8.03 (s, 1H, C17-H), 8.00 (s, 1H, C16-H), 7.99 – 7.88 (m, 3H, C11, C12, C14-H), 7.78 (d, *J* = 8.2 Hz, 2H, C1, C3-H), 7.73 – 7.52 (m, 3H, C4, C6, C13-H).

2.77. (Z)-3-(1-phenyl-1H-1,2,3-triazol-4-yl)-2-(4-(trifluoromethoxy)phenyl)acrylonitrile (7a)

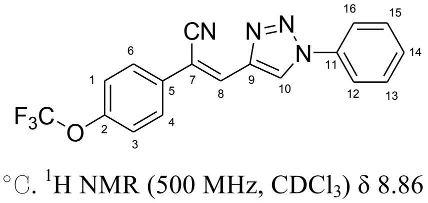

White powder; yield 31%. M.p.198-200°C. ^{1}H NMR (500 MHz, $CDCl_3$) δ 8.86 (s, 1H, C10-H), 7.84 (s, 1H, C8-H), 7.75 (d, *J* = 7.6 Hz, 2H, C12, C16-H), 7.67 (d, *J* = 8.5 Hz, 2H, C13, C15-H), 7.52 (t, *J* = 7.5 Hz, 2H, C4, C6-H), 7.26 (d, *J* = 8.1 Hz, 2H, C1, C3-H), 7.19 (s, 1H, C14-H).

2.78. (Z)-3-(1-(3-methoxyphenyl)-1H-1,2,3-triazol-4-yl)-2-(4-(trifluoromethoxy)phenyl) acrylonitrile (7b)

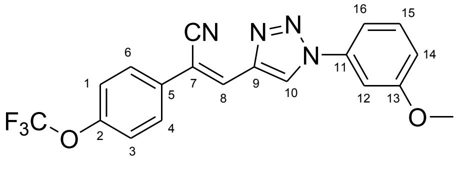

Light yellow powder; yield 40%. M.p.140-142°C. ^{1}H NMR (500 MHz, $CDCl_3$) δ 8.83 (s, 1H, C10-H), 7.82 (s, 1H, C8-H), 7.67 (d, *J* = 8.6 Hz, 2H, C4, C6-H), 7.42 – 7.37 (m, 1H, C16-H), 7.33 (s, 1H, C12-H), 7.28 – 7.23 (m, 2H, C1, C3-H), 7.19 (s, 1H, C15-H), 6.98 (s, 1H, C14-H), 3.84 (s, 3H, C13-O$\underline{CH_3}$). ^{13}C NMR (75 MHz, DMSO-d6) δ 160.19 (s, C13), 148.71 (s, C2), 145.92 (s, C9), 142.30 (s, C11), 137.10 (s, C8), 132.22 (s, C5), 131.64 (s, C15), 130.88 (s, C4, C6), 127.84 (s, C10), 124.12 (s, C1, C3), 121.59 (s, C2-O$\underline{C}F_3$), 116.96 (s, C7-CN), 114.98 (s, C16), 112.71 (s, C14), 109.72 (s, C12), 106.46 (s, C7), 55.64 (s, C13-O$\underline{C}H_3$). HRMS (ESI) m/z calcd for $C_{19}H_{14}F_3N_4O_2$ ($[M+H]^+$) 387.10634, found 387.10669.

2.79. (Z)-3-(1-(3,4,5-trimethoxyphenyl)-1H-1,2,3-triazol-4-yl)-2-(4-(trifluoromethoxy)phenyl)acrylonitrile (7c)

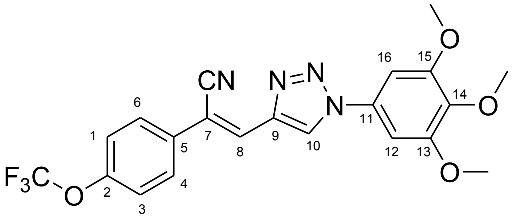

White flaky crystal; yield 49%. M.p.170-172°C. ^{1}H NMR (300 MHz, $CDCl_3$) δ 8.87 (s, 1H, C10-H), 7.92 (s, 1H, C8-H), 7.76 (d, *J* = 8.8 Hz, 2H, C4, C6-H), 7.35 (d, *J* = 8.3 Hz, 2H, C12, C16-H), 7.02 (s, 2H, C1, C3-H), 3.96 (d, *J* = 15.3 Hz, 9H, C13, C14, C15-O$\underline{CH_3}$).

2.80. (2Z)-3-(4-((E)-3-oxo-3-phenylprop-1-enyl)phenyl)-2-(4-(trifluoromethoxy)phenyl)acrylonitrile (8b)

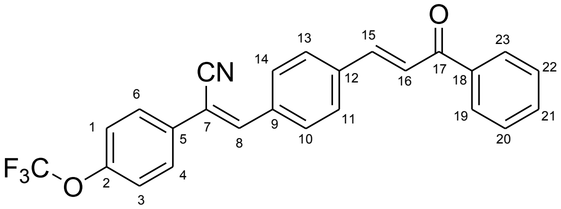

Light yellow powder; yield 51%. M.p.155-157°C. ^{1}H NMR (300 MHz, $CDCl_3$) δ 8.09 (s, 2H, C19, C23-H), 8.03 (s, 1H, C8-H), 7.98 (d, *J* = 8.2 Hz, 2H, C15, C16-H), 7.78 (s, 2H, C4, C6-H), 7.76 (d, *J* = 2.8 Hz, 2H, C10, C14-H), 7.55 (d, *J* = 3.3 Hz, 2H, C11, C13-H,), 7.34 (d, *J* = 8.2 Hz, 2H, C20, C22-H), 7.22 (s, 2H, C1, C3-H), 7.03 (d, *J* = 8.9 Hz, 1H, C21-H).

2.81.(2Z)-3-(4-((E)-3-(4-fluorophenyl)-3-oxoprop-1-enyl)phenyl)-2-(4-(trifluoromethoxy)phenyl)acrylonitrile (8c)

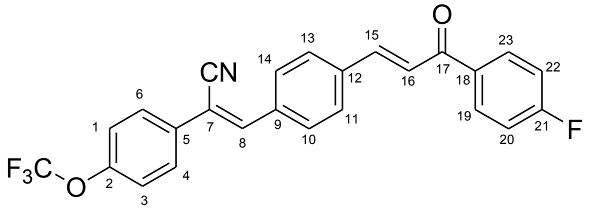

Light yellow powder; yield 41%. M.p.140-142°C. ^{1}H NMR (300 MHz, $CDCl_3$) *δ* 8.01 (d, *J* = 7.0 Hz, 2H, C19, C23-H), 7.64 (s, 1H, C8-H), 7.55 (s, 2H, C15, C16-H), 7.52 (d, *J* = 8.2 Hz, 2H, C4, C6-H), 7.18 (s, 2H, C20, C22-H), 7.12 (d, *J* = 6.0 Hz, 4H, C10, C14, C11, C13-H), 6.97 (d, *J* = 4.9 Hz, 2H, C1, C3-H).

2.82. (2Z)-3-(4-((E)-3-(4-bromophenyl)-3-oxoprop-1-enyl)phenyl)-2-(4-(trifluoromethoxy)phenyl)acrylonitrile (8d)

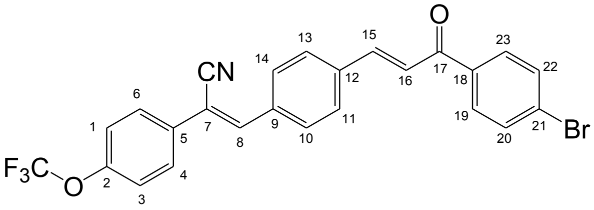

Light yellow powder; yield 41%. M.p.145-147°C. ^{1}H NMR (300 MHz, $CDCl_3$) δ 7.89 (s, 2H, C19, C23-H), 7.86 (s, 2H, C20, C22-H), 7.78 (d, *J* = 7.6 Hz, 2H, C15, C16-H), 7.68 (s, 2H, C4, C6-H), 7.66 (s, 1H, C8-H), 7.17 (s, 4H, C10, C14, C11, C13-H), 6.95 (d, *J* = 5.4 Hz, 2H, C1, C3-H).

2.83. (Z)-3-(1-benzyl-1H-indol-3-yl)-2-(4-(trifluoromethoxy)phenyl)acrylonitrile (9)

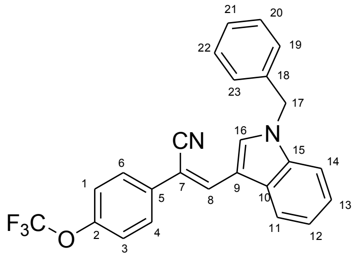

Yellow powder; yield 21%. M.p.118-120°C. ^{1}H NMR (300 MHz, $CDCl_3$) δ 8.47 (s, 1H, C16-H), 7.88 (s, 1H, C8-H), 7.81 (dd, *J* = 6.0, 3.1 Hz, 1H, C11-H), 7.71 (d, *J* = 8.7 Hz, 2H, C4, C6-H), 7.36 (s, 2H, C12, C13-H), 7.33 (s, 4H, C19, C20, C22, C23-H), 7.30 (d, *J* = 3.2 Hz, 2H, C14, C21-H), 7.22 (s, 2H, C1, C3-H), 5.45 (s, 2H, C17-H).

REFERENCES

[1] Ma, J.; Li, J.; Tian Y.S. Synthesis and bioactivity evaluation of 2,3-diaryl acrylonitrile derivatives as potential anticancer agents. *Bioorg. Med. Chem. Lett*. **2017**, *27*, 81-85.

[2] Xin, Y.B.; Li, J.J.; Zhang, H.J.; Ma, J.; Liu, X.; Gong, G.H.; Tian, Y.S. Synthesis and characterisation of (Z)-styrylbenzene derivatives as potential selective anticancer agents. *J Enzyme Inhib Med Chem*. **2018**, *33*, 1554-1564.

[3] Dai, Z.C.; Chen, Y.F.; Zhang, M.; Li, S.K.; Yang, T.T.; Shen, L.; Wang, J.X.; Qian, S.S.; Zhu, H.L.; Ye, Y.H. Synthesis and antifungal activity of 1,2,3-triazole phenylhydrazone derivatives. *Org. Biomol. Chem*. **2015**, *13*, 477-486.

[4] Gonzaga, D.; Senger, M.R.; da Silva Fde, C.; Ferreira, V.F.; Silva Jr, F.P. 1-Phenyl-1*H*- and 2-phenyl-2*H*-1,2,3-triazol derivatives: design, synthesis and inhibitory effect on alpha-glycosidases. *Eur. J. Med. Chem*. **2014**, *74*, 461-476.

[5] Carr, M.; Greene, L.M.; Knox, A.J.; Lloyd, D.G.; Zisterer, D.M.; Meegan, M.J. Lead identification of conformationally restricted β-lactam type combretastatin analogues: synthesis, antiproliferative activity and tubulin targeting effects. *Eur. J. Med. Chem.* **2010**, *45*, 5752-5766.

[6] Mi, C.; Wang, Z.; Li, M.Y.; Zhang, Z.H.; Ma, J.; Jin, X. Zinc finger protein 91 positively regulates the production of IL-1β in macrophages by activation of MAPKs and non-canonical caspase-8 inflammasome. *Br. J. Pharmacol.* **2018**, *175*, 4338-4352.

[7] Li, J.J.; Ma, J.; Xin, Y.B.; Quan, Z.S.; Tian Y.S. Synthesis and pharmacological evaluation of 2,3-diphenyl acrylonitrile bearing halogen as selective anticancer agents. *Chem. Biol. Drug Des*. **2018**, *92*, 1419-1428.

[8] Zhang, H.J.; Li, Y.F.; Cao, Q.; Tian, Y.S.; Quan, Z.S. Pharmacological evaluation of 9,10-dihydrochromeno[8,7-*e*][1,3] oxazin-2(8*H*)-one derivatives as potent anti-inflammatory agent. *Pharmacol. Rep*. **2017**, *69*, 419-425.

[9] Mustafa, M.; Abdelhamid, D.; Abdelhafez, E.; Ibrahim, M.; Gamal-Eldeen, A.; Aly, O.M. Synthesis, antiproliferative, anti-tubulin activity, and docking study of new 1,2,4-triazoles as potential combretastatin analogues. *Eur. J. Med. Chem*. **2017**, *141*, 293-305.

[10] Shen,Q.K.; Deng, H.; Wang, S.B.; Tian, Y.S.; Quan, Z.S. Synthesis, and evaluation of *in vitro* and *in vivo* anticancer activity of 14-substituted oridonin analogs: a novel and potent cell cycle arrest and apoptosis inducer through the p53-MDM2 pathway. *Eur. J. Med. Chem*. **2019**, *173*, 15-31.

SUPPLEMENTARY SCHEMES, TABLES AND FIGURES

2.1. Supplementary schemes

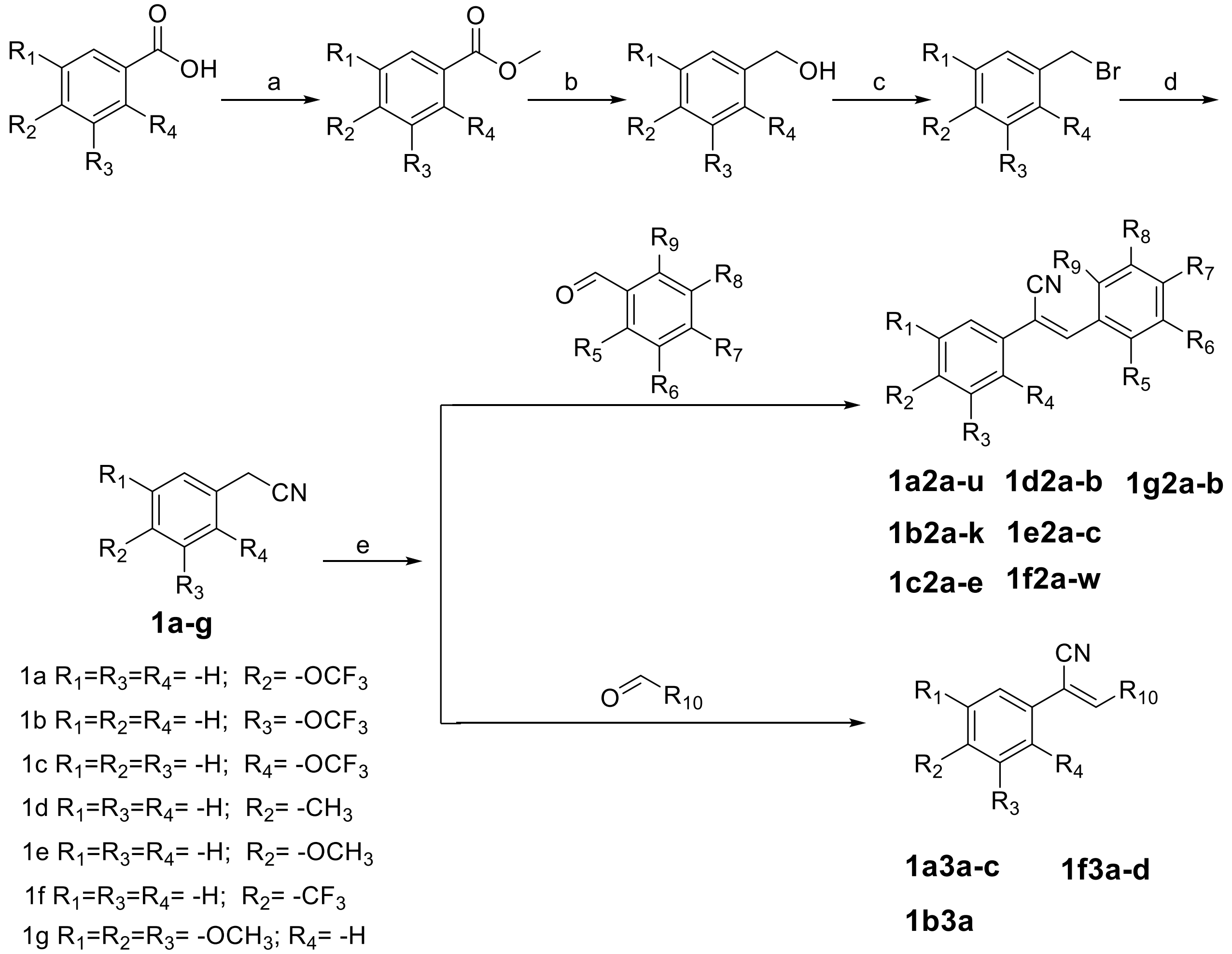

Scheme S1. Reagents and conditions: (a) H_2SO_4, CH_3OH, reflux, 4 h; (b) $LiAlH_4$, THF, 0°C-rt, 4-6 h; (c) CH_2Cl_2, PBr_3, 0°C-rt, 3-6 h; (d) CH_3CN, TMSCN, TBAF, reflux, 4-6 h; (e) CH_3OH, CH_3ONa, aromatic aldehydes, 4-6 h.

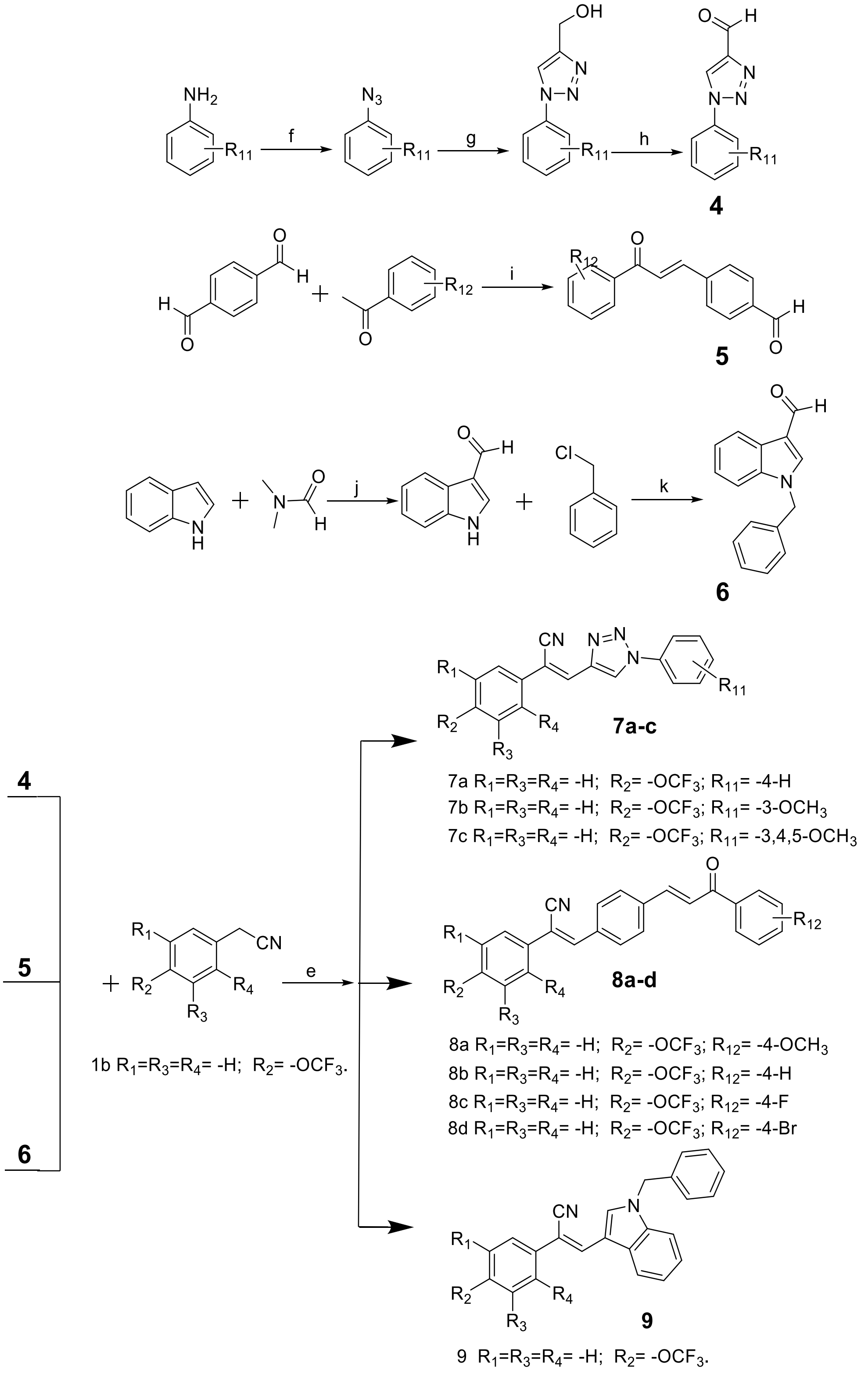

Scheme S2. Reagents and conditions: (e) CH_3OH, CH_3ONa, aromatic aldehydes, 4-6 h; (f) $NaNO_2$, HCl 10%, 0-5°C; NaN_3, RT, 2–4 h; (g) propargyl alcohol, $CuSO_4$ pentahydrate, sodium ascorbate, H_2O: *t*-butanol=1:1, 3 h, rt; (h) IBX/DMSO, 4 h, rt; (i) KOH, dry ethanol, stirred at RT, 12 h; (j) $POCl_3$, DMF, 0°C-RT, 2 h; (k) NaH, THF, 0°C-RT, 24 h.

4.2. Supplementary tables

Table S1. ***In vitro*** **anti-proliferative activity of the synthesized compounds, CA-4, CA-4P, colchicine, resveratrol and taxol against thirteen cell lines[a] (IC_{50} (μM[b]))**

		IC_{50} (μM)												
	**Agent**	**A549**	**AGS**	**BEL-7402**	**HCT-116**	**HeLa**	**HepG-2**	**L-02**	**MGC-803**	**MCF-7**	**MCF-10A**	**Raji**	**SGC-7901**	**SU-DHL-10**
1	**1a2a**	>100	>100	>100	>100	>100	>100	>100	>100	>100	>100	>100	>100	>100
2	**1a2b**	>100	13.2±3.3	15.0±2.7	23.5±4.6	35.9±.2.5	>100	>100	37.4±5.8	>100	>100	49.0±7.4	>100	38.8±3.7
3	**1a2c**	65.8±8.9	18.6±3.0	>100	20.2±1.7	72.3±2.4	20.7±1.4	>100	35.2±2.8	55.2±2.8	>100	55.1±3.2	56.4±9.7	>100
4	**1a2d**	>100	19.6±2.9	>100	25.4±6.8	87.3±4.1	24.3±3.4	>100	>100	>100	>100	64.9±1.5	>100	73.5±17.3
5	**1a2e**	>100	30.8±7.9	>100	>100	>100	>100	>100	>100	>100	>100	>100	>100	>100
6	**1a2f**	>100	14.6±5.9	>100	43.5±3.8	>100	>100	>100	56.7±11.0	59.9±2.3	>100	42.7±3.4	77.9±12.6	15.1±11.6
7	**1a2g**	71.6±24.7	8.7±2.1	>100	57.4±7.8	>100	>100	>100	>100	>100	>100	66.6±13.3	89.9±5.6	27.3±5.3
8	**1a2h**	>100	69.6±15.7	24.1±2.8	8.7±1.7	>100	>100	>100	16.8±0.7	>100	>100	67.4±31.7	19.7±1.5	>100
9	**1a2i**	>100	>100	>100	>100	>100	>100	>100	>100	>100	>100	45.1±15.6	>100	>100
10	**1a2j**	>100	21.1±5.2	>100	42.5±12.3	>100	72.6±25.3	>100	>100	>100	94.1±3.5	>100	>100	>100
11	**1a2k**	>100	>100	>100	>100	>100	>100	>100	>100	>100	>100	>100	>100	>100
12	**1a2l**	>100	>100	>100	>100	>100	>100	>100	>100	>100	>100	>100	>100	>100
13	**1a2m**	>100	87.2±5.9	>100	>100	>100	>100	>100	>100	>100	>100	>100	>100	>100
14	**1a2n**	>100	>100	>100	>100	>100	>100	>100	>100	>100	>100	>100	>100	>100
15	**1a2o**	>100	>100	>100	>100	>100	>100	>100	>100	>100	>100	>100	>100	>100
16	**1a2p**	>100	>100	>100	>100	>100	>100	>100	>100	>100	>100	>100	>100	>100
17	**1a2q**	>100	>100	>100	>100	>100	>100	>100	>100	>100	>100	>100	>100	>100
18	**1a2r**	>100	>100	>100	>100	>100	>100	>100	>100	>100	>100	>100	>100	>100
19	**1a2s**	>100	>100	>100	>100	>100	>100	>100	>100	>100	>100	>100	>100	>100
20	**1a2t**	>100	89.8±1.0	>100	30.8±2.9	>100	>100	>100	41.5±2.1	>100	>100	>100	57.2±14.3	>100
21	**1a2u**	>100	>100	>100	>100	>100	>100	>100	>100	>100	>100	>100	>100	>100
22	**1a3a**	>100	>100	>100	>100	>100	>100	>100	>100	>100	>100	>100	>100	>100
23	**1a3b**	>100	89.8±1.0	>100	30.8±2.9	>100	>100	>100	41.5±2.1	>100	>100	>100	57.2±14.3	>100
24	**1a3c**	>100	>100	>100	>100	>100	>100	>100	>100	>100	>100	>100	>100	>100
25	**1b2a**	>100	>100	>100	>100	>100	>100	>100	>100	>100	>100	>100	>100	>100
26	**1b2b**	>100	8.2±2.6	3.0±1.0	1.1±0.3	>100	15.9±5.2	16.1±1.0	1.2±0.6	>100	11.3±2.3	17.0±4.3	5.4±0.2	60.4±0.7
27	**1b2c**	>100	>100	>100	>100	>100	>100	>100	>100	>100	>100	>100	>100	>100
28	**1b2d**	>100	13.9±4.2	14.6±4.6	15.1±2.9	66.8±9.8	34.9±3.2	50.4±0.8	10.1±0.9	>100	34.1±3.6	16.3±6.1	21.9±3.3	35.6±0.9
29	**1b2e**	>100	4.5±2.0	39.4±6.1	25.0±0.5	61.7±3.3	38.2±0.1	54.2±12.3	11.1±0.3	>100	>100	43.1±4.5	8.1±2.9	29.3±2.0
30	**1b2f**	>100	43.8±21.8	44.8±10.6	>100	>100	72.9±9.9	>100	49.3±0.3	>100	>100	>100	61.7±0.4	>100
31	**1b2g**	>100	>100	>100	>100	>100	>100	>100	>100	>100	>100	>100	>100	>100
32	**1b2h**	>100	>100	1.0±0.8	0.3±0.2	>100	42.3±18.2	52.0±8.4	3.7±1.3	>100	>100	20.1±2.3	7.1±2.4	>100
33	**1b2i**	>100	23.9±2.7	13.0±1.1	12.3±1.6	>100	23.5±4.3	28.5±0.8	10.8±2.9	>100	79.6±5.7	23.7±2.1	46.0±4.9	53.6±7.5
34	**1b2j**	>100	>100	>100	>100	>100	>100	>100	>100	>100	>100	>100	72.1±4.5	>100

35	**1b2k**	>100	>100	>100	>100	>100	>100	>100	>100	>100	>100	>100	>100	>100
36	**1b3a**	32.6±1.1	15.4±0.5	>100	19.3±0.1	38.8±3.5	21.5±7.6	35.9±1.6	20.7±1.5	25.5±0.4	22.7±3.5	56.6±5.8	20.9±5.1	59.7±0.8
37	**1c2a**	>100	>100	>100	>100	>100	>100	>100	>100	>100	>100	>100	>100	>100
38	**1c2b**	>100	31.4±8.3	>100	>100	>100	70.5±25.4	>100	29.0±8.3	>100	>100	>100	>100	>100
39	**1c2c**	>100	16.7±2.3	>100	17.2±5.2	>100	27.9±2.4	42.2±11.5	19.6±6.7	>100	>100	42.4±1.7	>100	>100
40	**1c2d**	50.7±7.4	6.3±0.4	17.4±4.2	>100	>100	26.7±10.1	>100	13.1±3.6	>100	>100	60.5±19.1	50.1±5.9	>100
41	**1c2e**	>100	7.6±0.6	10.8±2.3	8.4±2.4	75.8±12.4	24.9±7.0	36.1±4.8	2.5±1.1	>100	>100	41.3±5.5	7.8±0.8	>100
42	**1d2a**	>100	23.0±1.0	>100	>100	>100	>100	>100	18.8±1.7	>100	>100	7.9±0.5	>100	19.9±0.7
43	**1d2b**	>100	24.1±0.2	65.4±2.5	3.1±0.5	>100	>100	>100	8.6±0.04	52.3±2.2	>100	11.8±0.4	>100	42.6±11.4
44	**1e2a**	>100	18.4±1.9	46.8±5.1	7.2±0.6	>100	>100	47.5±12.4	8.3±1.2	36.9±3.6	28.1±3.6	11.6±4.9	38.2±9.0	27.2±5.3
45	**1e2b**	>100	>100	>100	>100	>100	>100	>100	15.1±1.2	>100	>100	12.7±3.8	22.6±0.0	>100
46	**1e2c**	>100	>100	>100	>100	>100	>100	>100	>100	>100	>100	19.3±0.5	>100	62.3±9.6
47	**1f2a**	>100	>100	>100	>100	>100	>100	>100	>100	>100	>100	>100	>100	>100
48	**1f2b**	>100	>100	>100	33.0±5.8	>100	>100	>100	>100	>100	>100	>100	>100	>100
49	**1f2c**	>100	>100	>100	>100	>100	>100	>100	>100	>100	>100	>100	>100	>100
50	**1f2d**	>100	>100	>100	>100	>100	>100	>100	51.9±2.1	>100	>100	>100	>100	>100
51	**1f2e**	>100	>100	>100	>100	>100	>100	>100	>100	>100	>100	>100	>100	>100
52	**1f2f**	>100	>100	>100	>100	>100	>100	>100	>100	>100	>100	>100	>100	>100
53	**1f2g**	>100	>100	>100	>100	>100	>100	>100	>100	>100	>100	>100	>100	>100
54	**1f2h**	>100	>100	>100	>100	>100	>100	>100	>100	>100	>100	>100	>100	58.4±9.0
55	**1f2i**	>100	42.0±11.7	>100	>100	>100	>100	>100	>100	>100	>100	>100	>100	>100
56	**1f2j**	>100	>100	>100	>100	>100	>100	>100	>100	>100	>100	>100	>100	>100
57	**1f2k**	47.8±4.0	53.2±13.2	>100	>100	>100	>100	>100	>100	>100	>100	77.4±13.6	>100	>100
58	**1f2l**	>100	>100	>100	>100	>100	>100	>100	>100	>100	>100	72.1±7.2	>100	50.1±20.9
59	**1f2m**	>100	>100	>100	>100	>100	>100	>100	>100	>100	>100	>100	>100	>100
60	**1f2n**	37.9±1.0	46.4±15.0	>100	57.3±3.2	>100	>100	>100	>100	>100	>100	>100	>100	>100
61	**1f2o**	>100	41.2±1.1	>100	>100	>100	>100	>100	>100	>100	>100	>100	>100	>100
62	**1f2p**	>100	>100	>100	>100	>100	>100	>100	>100	>100	>100	>100	>100	>100
63	**1f2q**	>100	>100	>100	>100	>100	>100	>100	>100	>100	>100	34.9±4.5	>100	38.4±3.6
64	**1f2r**	>100	>100	>100	>100	>100	>100	>100	>100	>100	>100	>100	>100	>100
65	**1f2s**	>100	>100	>100	47.8±0.7	>100	>100	>100	>100	>100	>100	>100	>100	>100
66	**1f2t**	>100	77.5±3.9	>100	33.2±0.2	>100	>100	>100	>100	>100	>100	>100	>100	>100
67	**1f2u**	>100	>100	>100	>100	>100	>100	>100	>100	>100	92.5±10.2	>100	>100	>100
68	**1f2v**	>100	>100	>100	>100	>100	>100	>100	>100	>100	>100	>100	>100	>100
69	**1f2w**	>100	>100	>100	>100	>100	>100	>100	>100	>100	>100	0.048±0.0	>100	0.022±0.0
70	**1f3a**	>100	>100	>100	>100	>100	>100	>100	>100	>100	>100	>100	>100	>100
71	**1f3b**	>100	65.0±17.9	>100	>100	>100	>100	>100	>100	>100	>100	>100	>100	>100
72	**1f3c**	>100	>100	>100	87.6±9.1	>100	>100	>100	>100	>100	>100	>100	>100	>100
73	**1f3d**	>100	>100	>100	>100	>100	>100	>100	>100	>100	>100	>100	>100	>100
74	**1g2a**	>100	0.08±0.05	0.007±0.0	0.005±0.0	>100	>100	>100	14.0±8.4	0.27±0.04	0.02±0.00	0.03±0.00	>100	0.03±0.01

75	**1g2b**	>100	7.5±0.4	0.03±0.01	1.0±0.1	>100	>100	>100	0.12±0.03	0.45±0.06	0.05±0.00	0.37±0.06	>100	2.4±0.1
76	**7a**	>100	>100	>100	>100	>100	>100	>100	>100	>100	>100	>100	>100	>100
77	**7b**	>100	>100	>100	>100	>100	>100	>100	>100	>100	>100	>100	>100	>100
78	**7c**	>100	>100	>100	>100	>100	>100	>100	>100	>100	>100	>100	>100	>100
79	**8a**	>100	>100	13.2±2.7	56.3±8.4	>100	>100	>100	>100	17.6±6.5	11.3±2.3	1.2±0.5	>100	1.3±0.1
80	**8b**	>100	>100	>100	>100	>100	>100	>100	>100	>100	>100	>100	>100	>100
81	**8c**	>100	25.3±7.5	77.4±3.0	22.9±6.2	>100	>100	>100	87.5±0.6	67.7±5.4	15.8±2.5	1.0±0.2	22.2±8.6	1.0±0.2
82	**8d**	>100	31.8±6.1	>100	35.3±6.5	>100	>100	>100	>100	53.1±7.1	37.3±4.3	3.0±1.0	>100	7.1±2.6
83	**9**	>100	>100	>100	>100	>100	>100	>100	>100	>100	>100	>100	>100	>100
	CA-4	>100	3.4±0.0	2.0±0.03	0.16±0.00	>100	>100	1.1±0.03	0.06±0.02	0.2±0.1	3.2±1.2	0.18±0.00	0.30±0.00	1.1±0.1
	CA-4P	>100	4.7±0.2	5.4±2.0	0.5±0.2	>100	>100	2.6±0.6	0.03±0.02	0.4±0.2	14.5±1.3	0.42±0.01	0.1±0.03	0.88±0.07
	Taxol	0.4±0.02	0.02±0.01	0.09±0.02	0.03±0.01	12.9±0.4	0.9±0.03	>100	0.06±0.01	0.08±0.01	0.18±0.02	0.26±0.02	>100	0.18±0.03
	Colchicine	6.5±1.5	0.1±0.03	0.02±0.00	0.01±0.00	1.8±0.1	1.7±0.4	0.4±0.02	0.13±0.01	0.6±0.3	1.4±0.3	0.3±0.1	0.1±0.04	0.29±0.00
	Resveratrol	>100	>100	42.5±5.4	16.7±3.6	>100	>100	12.8±0.2	>100	72.4±9.9	>100	>100	>100	>100

[a]Cytotoxicity as IC_{50} for each cell line, refers to the concentration of compound which reduced by 50% the optical density of treated cells with respect to untreated cells using the MTT assay.
[b]Data represent the mean values of three independent determinations.

Table S2. Selectivity index values of compounds relative to the effects on normal cell L-02 or MCF-10A cells.

		SI										
	**Agent**	**IC_{50} (L-02/ A549)**	**IC_{50} (L-02/ AGS)**	**IC_{50} (L-02/ BEL-7402)**	**IC_{50} (L-02/ HCT-116)**	**IC_{50} (L-02/ HeLa)**	**IC_{50} (L-02/ HepG-2)**	**IC_{50} (L-02/ MGC-803)**	**IC_{50}(MCF-10/ MCF-7)**	**IC_{50} (L-02/ Raji)**	**IC_{50} (L-02/ SGC-7901)**	**IC_{50} (L-02/ SU-DHL-10)**
2	**1a2b**	-	-	-	-	-	-	-	-	-	-	-
3	**1a2c**	> 1.5	> 5.3	-	> 4.9	> 1.3	> 4.8	> 2.8	> 1.8	> 1.8	> 1.7	-
4	**1a2d**	-	> 5.0	-	> 3.9	> 1.1	> 4.1	-	-	> 1.5	-	> 1.3
5	**1a2e**	-	> 3.2	-	-	-	-	-	-	-	-	-
6	**1a2f**	-	> 6.8	-	> 2.2	-	-	> 1.7	> 1.6	> 2.3	> 1.2	> 6.6
7	**1a2g**	> 1.3	> 11.4	-	> 1.7	-	-	-	-	> 1.5	> 1.1	> 3.6
8	**1a2h**	-	> 1.4	> 4.1	> 11.4	-	-	> 5.9	-	> 1.4	> 5.0	-
9	**1a2i**	-	-	-	-	-	-	-	-	> 2.2	-	-
10	**1a2j**	-	> 4.7	-	> 2.3	-	> 1.3	-	-	-	-	-
12	**1a2l**	-	-	-	-	-	-	-	-	-	-	-
13	**1a2m**	-	> 1.4	-	-	-	-	-	-	-	-	-
20	**1a2t**	-	> 1.1	-	> 3.2	-	-	> 2.4	-	-	> 1.7	-
23	**1a3b**	> 1.1	> 2.3	-	> 1.8	-	> 1.6	> 1.7	-	-	> 1.7	-
26	**1b2b**	-	> 1.9	> 5.2	> 14.5	-	> 1.0	> 13.1	-	-	> 2.9	-
28	**1b2d**	-	> 3.5	> 3.4	> 15.1	-	> 1.4	> 4.9	-	> 3.0	> 2.3	> 1.4
29	**1b2e**	-	> 11.9	> 1.3	> 2.1	-	> 1.4	> 4.8	-	> 1.2	> 6.6	> 1.8

30	**1b2f**	-	> 2.2	> 2.2	-	-	> 1.9	> 2.0	-	-	> 1.6	-
32	**1b2h**	-	-	> 50.5	> 153.1	-	> 1.3	> 13.9	-	> 2.5	> 7.2	-
33	**1b2i**	-	> 1.1	> 2.1	> 2.3	-	> 1.2	> 2.6	-	> 1.1	-	-
34	**1b2j**	-	-	-	-	-	-	-	-	-	> 1.3	-
36	**1b3a**	-	-	> 5.2	-	-	-	-	-	-	-	-
38	**1c2b**	-	> 3.1	-	-	-	> 1.4	> 3.4	-	-	-	-
39	**1c2c**	-	> 2.5	-	> 2.4	-	> 1.5	> 2.1	-	-	-	-
40	**1c2d**	> 1.9	> 15.6	> 5.7	-	-	> 3.7	> 7.6	-	> 1.6	> 1.9	-
41	**1c2e**	-	> 4.7	> 3.3	> 4.2	-	> 1.4	> 14.2	-	-	> 4.5	-
42	**1d2a**	-	> 4.3	-	-	-	-	> 5.3	-	> 12.5	-	> 5.0
43	**1d2b**	-	> 4.1	> 1.5	> 32.0	-	-	> 11.5	> 1.9	> 8.4	-	> 2.3
44	**1e2a**	-	> 2.8	> 1.0	> 6.5	-	-	> 5.7	-	> 4.0	> 1.2	> 1.7
45	**1e2b**	-	-	-	-	-	-	> 6.6	-	> 7.8	> 4.4	-
46	**1e2c**	-	-	-	-	-	-	-	-	> 5.1	-	> 1.6
48	**1f2b**	-	-	-	> 3.0	-	-	-	-	-	-	-
50	**1f2d**	-	-	-	-	-	-	> 1.9	-	-	-	-
54	**1f2h**	-	-	-	-	-	-	-	-	-	-	> 1.7
55	**1f2i**	-	> 2.3	-	-	-	-	-	-	-	-	-
57	**1f2k**	> 2.0	> 1.8	-	-	-	-	-	-	-	-	-
58	**1f2l**	-	-	-	-	-	-	-	-	> 1.3	-	> 1.9
59	**1f2m**	> 2.6	> 2.1	-	> 1.7	-	-	-	-	-	-	-
60	**1f2n**	-	> 2.4	-	-	-	-	-	-	-	-	-
63	**1f2q**	-	-	-	-	-	-	-	-	> 2.8	-	> 38
65	**1f2s**	-	-	-	> 2.0	-	-	-	-	-	-	-
66	**1f2t**	-	> 1.2	-	> 3.0	-	-	-	-	-	-	-
69	**1f2w**	-	-	-	-	-	-	-	-	> 2083	-	> 4545
71	**1f3b**	-	> 1.5	-	-	-	-	-	-	-	-	-
72	**1f3c**	-	-	-	> 1.1	-	-	-	-	-	-	-
74	**1g2a**	-	> 1250	> 12820	> 16949	-	-	> 7.1	-	> 2941	-	> 2941
75	**1g2b**	-	> 13.1	> 3333	> 96.1	-	-	> 819	-	> 270	-	> 34.0
80	**8b**	-	-	-	-	-	-	-	-	-	-	-
81	**8c**	-	> 3.9	> 1.2	> 4.3	-	-	> 1.1	-	> 99.0	> 4.5	> 96.1
82	**8d**	-	-	-	> 1.7	-	-	-	-	> 79.3	-	> 14.0
	CA-4	-	-	-	> 6.8	-	-	> 18.3	> 14.6	> 5.8	> 3.6	-
	CA-4P	-	-	-	> 4.5	-	-	> 89	> 30.3	> 6.2	> 22.2	> 3.0
	Taxol	> 227	> 5000	> 1111	> 3333	> 7.7	> 106	> 16666	> 2.2	> 384	-	> 555
	Colchicine	-	-	> 16.7	> 29.3	-	-	> 3.6	> 2.3	> 1.3	> 4.2	> 1.6
	Resveratrol	-	-	-	-	-	-	-	-	-	-	-

-: not suitable for calculation.

SI: selectivity index, $IC_{50\ (L\text{-}02\ or\ MCF\text{-}10A)}$ / $IC_{50\ (cancer\ cell)}$

Table S3. ADMET prediction parameters of some compounds.

Compound	Absorption[a]	Solubility[b]	BBB[c]	CYP2D6[d]	Hepatotoxicity[e]	PPB[f]
**1a2a**	0	-6.394	1.127	FALSE	FALSE	TRUE
**1a2f**	2	-7.451		FALSE	TRUE	TRUE
**1a2o**	1	-6.55	1.124	FALSE	TRUE	TRUE
**1a2p**	1	-6.948	1.339	FALSE	TRUE	TRUE
**1a2r**	1	-7.069	1.266	FALSE	TRUE	TRUE
**1a3c**	1	-7.741	1.407	FALSE	TRUE	TRUE
**1b2a**	0	-6.419	1.127	FALSE	FALSE	TRUE
**1b2g**	2	-7.483		FALSE	TRUE	TRUE
**1b2i**	0	-6.411	0.886	FALSE	TRUE	TRUE
**1b2h**	1	-6.582	1.124	FALSE	TRUE	TRUE
**1c2a**	0	-6.443	1.127	FALSE	FALSE	TRUE
**1c2d**	1	-6.931	1.277	FALSE	FALSE	TRUE
**1d2a**	0	-5.044	0.76	FALSE	TRUE	TRUE
**1d2b**	0	-5.5	0.976	FALSE	TRUE	TRUE
**1e2a**	0	-4.529	0.463	FALSE	TRUE	TRUE
**1e2b**	0	-4.971	0.679	FALSE	TRUE	TRUE
**1f2a**	0	-5.63	0.901	TRUE	TRUE	TRUE
**1f2b**	0	-6.045	1.117	FALSE	TRUE	TRUE
**1f2c**	0	-5.45	0.904	TRUE	FALSE	TRUE
**1f2s**	0	-5.419	0.758	FALSE	FALSE	TRUE
**1f3b**	0	-5.181	0.819	FALSE	TRUE	TRUE
**1g2a**	0	-4.284	0.903	FALSE	TRUE	TRUE
**1g2b**	0	-4.887	0.386	FALSE	TRUE	TRUE
**7a**	1	-6.704		FALSE	TRUE	TRUE
**8c**	3	-7.902		FALSE	TRUE	TRUE
**9**	3	-8.696		FALSE	TRUE	TRUE
**CA-4**	0	-4.289	0.036	FALSE	TRUE	TRUE
Taxol	3	-3.406		FALSE	TRUE	TRUE
Colchicine	0	-3.812	-0.838	FALSE	TRUE	FALSE
Resveratrol	0	-3.406	-0.187	FALSE	TRUE	TRUE

[a] prediction of intestinal absorption level of humans (acceptable range: 0 means good, 1 means medium, 2 means low).

[b] prediction of water solubility at room temperature (acceptable range: -4.1 < log(SW) < - 2.0 represents good water solubility; -6.0 < log(SW) < - 4.1 represents poor water solubility; -8.0 < log (SW) < -6.0 represents extremely poor water solubility, but there is the possibility).

[c] prediction of blood brain barrier (BBB) penetration level (acceptable range: 0.3 < AlogP98 < 1.0 and -1 < AlogP98 < -0.3 represent medium level; AlogP98 < 0.3 and -0.3 < AlogP98 < 0 represent low level; The blank represents no definition).

[d] prediction of human cytochrome P450 2D6 (CYP2D6) inhibition ability (acceptable level: False means no inhibition ability; True means it has inhibitory effect).

[e] prediction of hepatotoxicity (acceptable levels: False means non-hepatotoxicity, True means hepatotoxicity).

[f] prediction of plasma protein binding (PPB) possibility (acceptable levels: True means good, False means bad).

4.3. Supplementary Figures

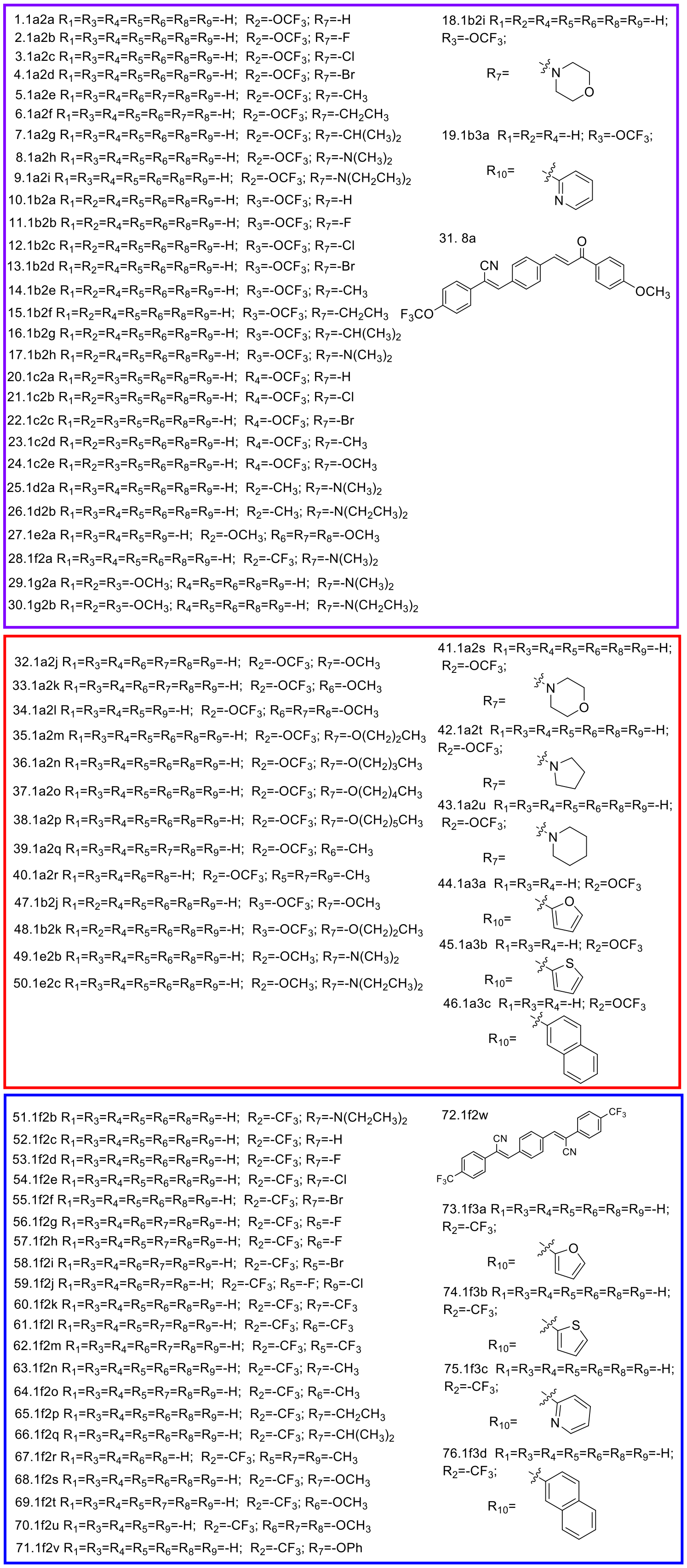

Figure S1. The compounds and their substituents were synthesized according to Scheme 1 and Scheme 2.

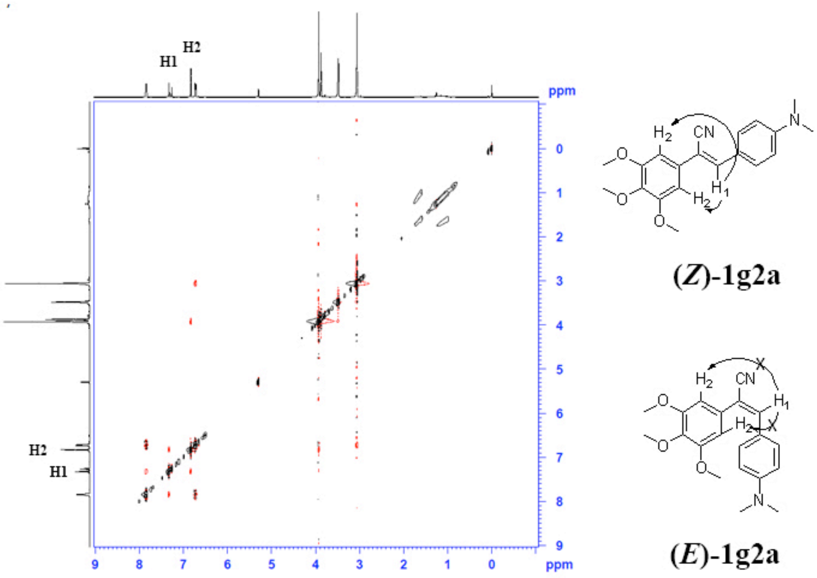

Figure S2. NOE (nuclear overhauser effect) result of compound **1g2a**.

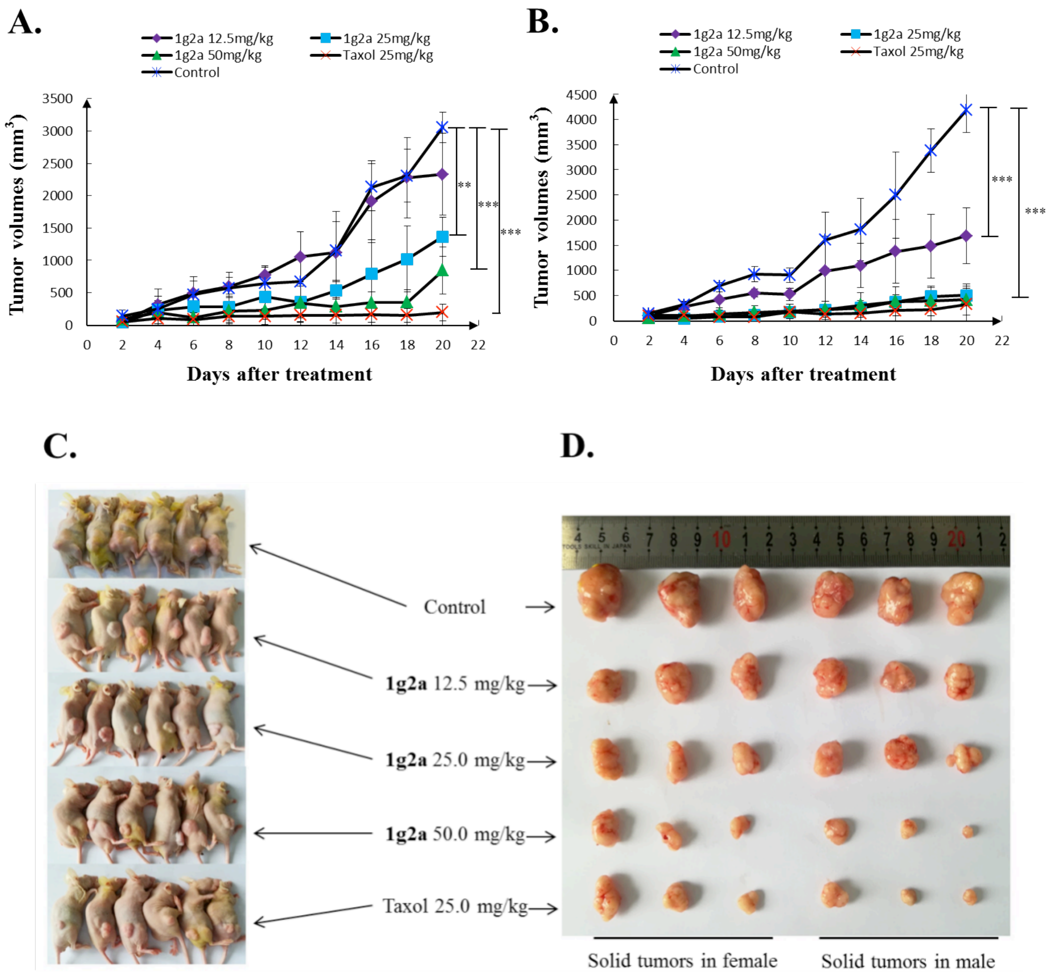

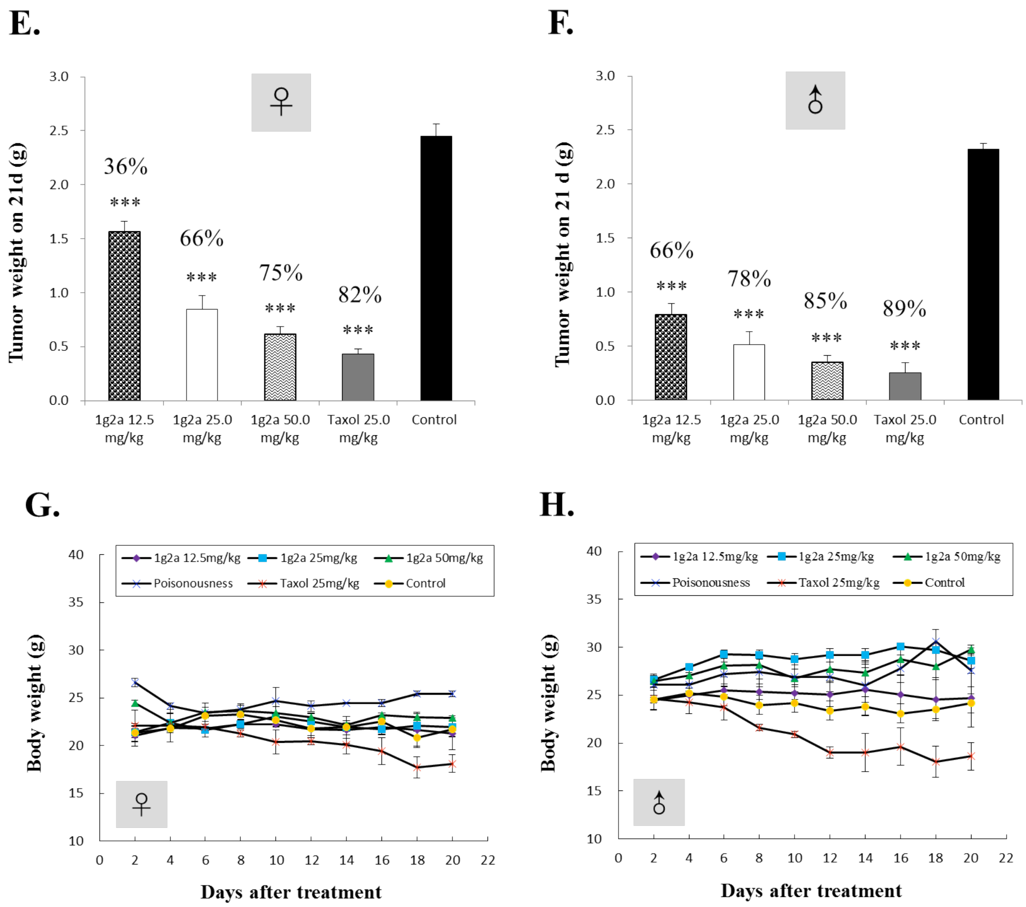

Figure S3. Compound **1g2a** inhibits colon cancer xenograft growth *in vivo*. (A and B) Relationship curves of tumor volumes at various times after administration in (A) female mice and (B) male mice. $**p < 0.01$ and $***p < 0.001$ compared to control group; (C) Representative photographs of experimental mice; (D) Image of the internal tumor tissues after anatomy. (E and F) Histograms display the changes of tumor weight from female and male mice, respectively, $***p < 0.001$ compared to control group; (G and H) Body weight changes of female (G) and male (H) mice during observation period. Data expressed as the mean ± SD (n=3). Statistical significance compared with vehicle by *t*-test.
